# Supplementary material for: Agreement between the laboratory-based and non-laboratory-based WHO cardiovascular risk charts: a cross-sectional analysis of a national health survey in Peru
Source: BMJ Open. 2022 Nov 7;12(11):e063289. doi: 10.1136/bmjopen-2022-063289 (PMC9644334; doi:10.1136/bmjopen-2022-063289)

Agreement between the laboratory- and non-laboratory-based WHO cardiovascular risk charts: a cross-sectional analysis of a national health survey in Peru

Correspondence author:

Rodrigo M Carrillo-Larco, MD

Department of Epidemiology and Biostatistics

School of Public Health

Imperial College London

rcarrill@ic.ac.uk

Table of Contents

Table of Contents 2

Supplementary Figure 1. Formula to compute the survey sample size........ 3

Supplementary Figure 2. Flowchart of data cleaning and inclusion criteria........ 3

Supplementary Table 1. Summary statistics of the first and second systolic blood pressure (SBP) records in the overall sample........ 4

Supplementary Table 2. Sensitivity Analysis: Lin's concordance coefficient correlation showing agreement between laboratory and non-laboratory-based risk models according to the predictors in the 2019 WHO CVD risk models and urban/rural location........ 4

Supplementary Table 3. Kappa statistics showing agreement between laboratory and non-laboratory-based risk scores in the overall sample........ 5

Supplementary Table 4. Kappa statistics showing agreement between laboratory and non-laboratory-based risk scores by sex........ 5

Supplementary Table 5. Kappa statistics showing agreement between laboratory and non-laboratory-based risk scores by age groups........ 5

Supplementary Table 6. Kappa statistics showing agreement between laboratory and non-laboratory-based risk scores by body mass index categories........ 6

Supplementary Table 7. Kappa statistics showing agreement between laboratory and non-laboratory-based risk scores by smoking status........ 6

Supplementary Table 8. Kappa statistics showing agreement between laboratory and non-laboratory-based risk scores by diabetes status........ 6

Supplementary Table 9. Kappa statistics showing agreement between laboratory and non-laboratory-based risk scores by urban/rural location........ 7

Supplementary Figure 3. Sensitivity Analysis: Bland Altman plots showing agreement between laboratory and non-laboratory-based risk scores according to sex......... 7

Supplementary Figure 4. Sensitivity Analysis: Bland Altman plots showing agreement between laboratory and non-laboratory-based risk scores according to age groups......... 8

Supplementary Figure 5. Sensitivity Analysis: Bland Altman plots showing agreement between laboratory and non-laboratory-based risk scores according to body mass index categories......... 9

Supplementary Figure 6. Sensitivity Analysis: Bland Altman plots showing agreement between laboratory and non-laboratory-based risk scores according to smoking status......... 10

Supplementary Figure 7. Sensitivity Analysis: Bland Altman plots showing agreement between laboratory and non-laboratory-based risk scores according to self-reported diabetes status........ 11

Guzman-Vilca WC, *et al. BMJ Open* 2022; 12:e063289. doi: 10.1136/bmjopen-2022-063289

Supplementary Figure 1. Formula to compute the survey sample size

$$n_h = \frac{N_h Z^2 P_h Q_h}{(N_h - 1)d^2 + Z^2 P_h Q_h} * TNR$$

Where:
N_h: number of people within an age group in the “h” conglomerate
N_h: number of people in the sample within an age group in the “h” conglomerate
d: error margin assumed in the estimation of P_h
Z: 95% confidence level
TNR (*Tasa de No Respuesta* in Spanish): Expected refusal rate
P_h: prevalence of overweight in adults in the “h” conglomerate

Supplementary Figure 2. Flowchart of data cleaning and inclusion criteria

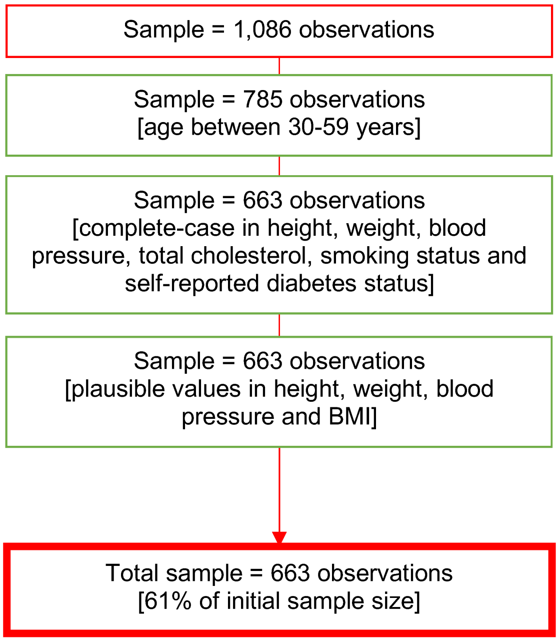

Guzman-Vilca WC, *et al. BMJ Open* 2022; 12:e063289. doi: 10.1136/bmjopen-2022-063289

Supplementary Table 1. Summary statistics of the first and second systolic blood pressure (SBP) records in the overall sample

	First SBP record	Second SBP record
**Minimum value**	78	75
**1st quartile**	101	99
**Median**	109	108
**Mean**	110.33	109.54
**3rd quartile**	118	118
**Maximum value**	203	198

Supplementary Table 2. Sensitivity Analysis: Lin's concordance coefficient correlation showing agreement between laboratory and non-laboratory-based risk models according to the predictors in the 2019 WHO CVD risk models and urban/rural location

Variables	Categories	Lin's concordance coefficient correlation (95% CI)
**Sex**	**Men**	0.87 (0.84 - 0.89)
**Sex**	**Women**	0.85 (0.83 - 0.87)
**Age (years)**	**30-39**	0.87 (0.84 - 0.9)
**Age (years)**	**40-49**	0.74 (0.68 - 0.79)
**Age (years)**	**50-59**	0.83 (0.78 - 0.87)
**Body mass index category**	**Normal**	0.9 (0.87 - 0.92)
**Body mass index category**	**Overweight**	0.87 (0.85 - 0.9)
**Body mass index category**	**Obese**	0.86 (0.82 - 0.89)
**Smoking status**	**Smoker**	0.82 (0.73 - 0.88)
**Smoking status**	**Non-smoker**	0.86 (0.84 - 0.88)
**Diabetes status**	**With self−reported diabetes**	0.74 (0.63 - 0.82)
**Diabetes status**	**Not with self−reported diabetes**	0.91 (0.9 - 0.92)
**Urban or rural**	**Urban**	0.88 (0.86 - 0.9)
**Urban or rural**	**Rural**	0.86 (0.82 - 0.88)

Guzman-Vilca WC, *et al. BMJ Open* 2022; 12:e063289. doi: 10.1136/bmjopen-2022-063289

Supplementary Table 3. Kappa statistics showing agreement between laboratory and non-laboratory-based risk scores in the overall sample

	Non-laboratory-based-risk category			
**Laboratory-based-risk category**	**0-5**	**5-9**	**10-19**	**kappa**
**0-5**	618	4	0	0.62
**5-9**	17	19	1	
**10-19**	0	3	1	

Supplementary Table 4. Kappa statistics showing agreement between laboratory and non-laboratory-based risk scores by sex

		Non-laboratory-based-risk category			
**Laboratory-based-risk category**	**Sex**	**0-5**	**5-9**	**10-19**	**kappa**
**0-5**	Men	251	4	0	0.7
**5-9**	Men	7	15	1	
**10-19**	Men	0	1	1	
**0-5**	Women	367	0	0	0.44
**5-9**	Women	10	4	0	
**10-19**	Women	0	2	0	

Supplementary Table 5. Kappa statistics showing agreement between laboratory and non-laboratory-based risk scores by age groups

		Non-laboratory-based-risk category			
**Laboratory-based-risk category**	**Age group**	**0-5**	**5-9**	**10-19**	**kappa**
**0-5**	30-39	236	0	0	0
**5-9**	30-39	1	0	0	
**10-19**	30-39	0	0	0	
**0-5**	40-49	216	1	0	0.45
**5-9**	40-49	4	2	1	
**10-19**	40-49	0	1	0	
**0-5**	50-59	166	3	0	0.65
**5-9**	50-59	12	17	0	
**10-19**	50-59	0	2	1	

Supplementary Table 6. Kappa statistics showing agreement between laboratory and non-laboratory-based risk scores by body mass index categories

		Non-laboratory-based-risk category			
**Laboratory-based-risk category**	**Body mass index category**	**0-5**	**5-9**	**10-19**	**kappa**
**0-5**	Normal	161	1	0	0.66
**5-9**	Normal	1	2	0	
**10-19**	Normal	0	0	0	
**0-5**	Obese	202	3	0	0.65
**5-9**	Obese	7	11	1	
**10-19**	Obese	0	2	1	
**0-5**	Overweight	255	0	0	0.55
**5-9**	Overweight	9	6	0	
**10-19**	Overweight	0	1	0	

Supplementary Table 7. Kappa statistics showing agreement between laboratory and non-laboratory-based risk scores by smoking status

		Non-laboratory-based-risk category			
**Laboratory-based-risk category**	**Smoking status**	**0-5**	**5-9**	**10-19**	**kappa**
**0-5**	Non-smoker	568	3	0	0.53
**5-9**	Non-smoker	12	9	0	
**10-19**	Non-smoker	0	1	0	
**0-5**	Smoker	50	1	0	0.67
**5-9**	Smoker	5	10	1	
**10-19**	Smoker	0	2	1	

Supplementary Table 8. Kappa statistics showing agreement between laboratory and non-laboratory-based risk scores by diabetes status

		Non-laboratory-based-risk category			
**Laboratory-based-risk category**	**Diabetes status**	**0-5**	**5-9**	**10-19**	**kappa**
**0-5**	Not with self−reported diabetes	592	4	0	0.71
**5-9**	Not with self−reported diabetes	7	15	1	
**10-19**	Not with self−reported diabetes	0	0	0	
**0-5**	With self−reported diabetes	26	0	0	0.36
**5-9**	With self−reported diabetes	10	4	0	
**10-19**	With self−reported diabetes	0	3	1	

Supplementary Table 9. Kappa statistics showing agreement between laboratory and non-laboratory-based risk scores by urban/rural location

		Non-laboratory-based-risk category			
**Laboratory-based-risk category**	**Urban/Rural**	**0-5**	**5-9**	**10-19**	**kappa**
**0-5**	Rural	224	1	0	0.53
**5-9**	Rural	4	3	0	
**10-19**	Rural	0	1	0	
**0-5**	Urban	394	3	0	0.64
**5-9**	Urban	13	16	1	
**10-19**	Urban	0	2	1	

Supplementary Figure 3. Sensitivity Analysis: Bland Altman plots showing agreement between laboratory and non-laboratory-based risk scores according to sex.

Guzman-Vilca WC, *et al. BMJ Open* 2022; 12:e063289. doi: 10.1136/bmjopen-2022-063289

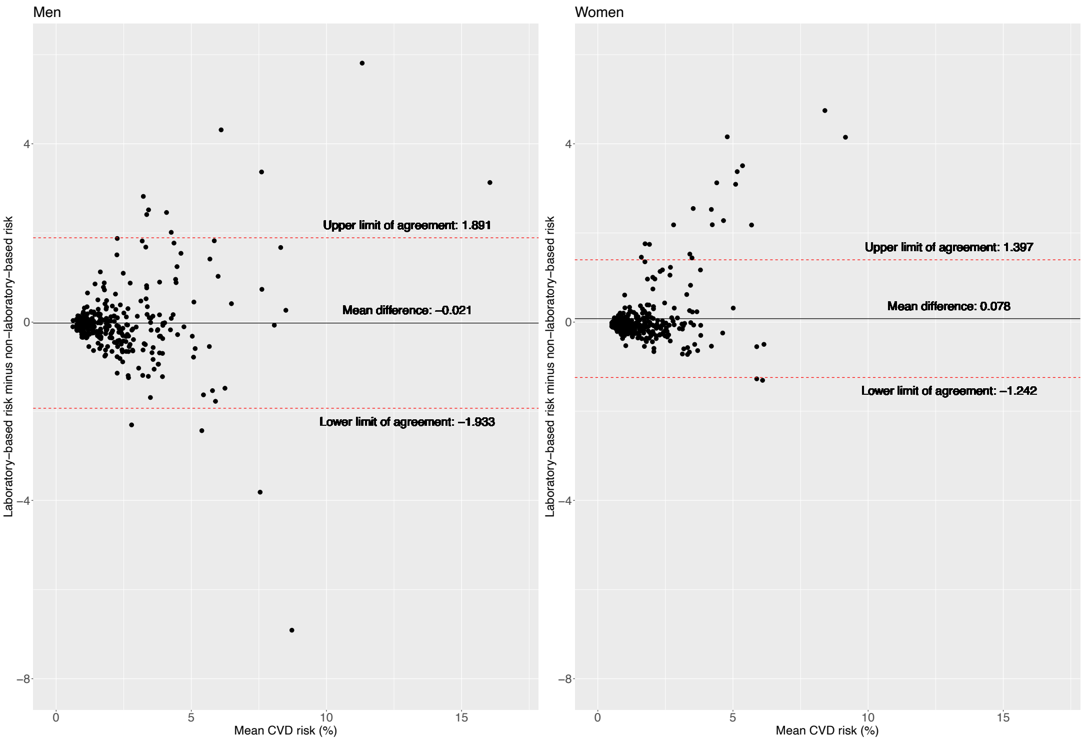

Supplementary Figure 4. Sensitivity Analysis: Bland Altman plots showing agreement between laboratory and non-laboratory-based risk scores according to age groups.

Guzman-Vilca WC, *et al. BMJ Open* 2022; 12:e063289. doi: 10.1136/bmjopen-2022-063289

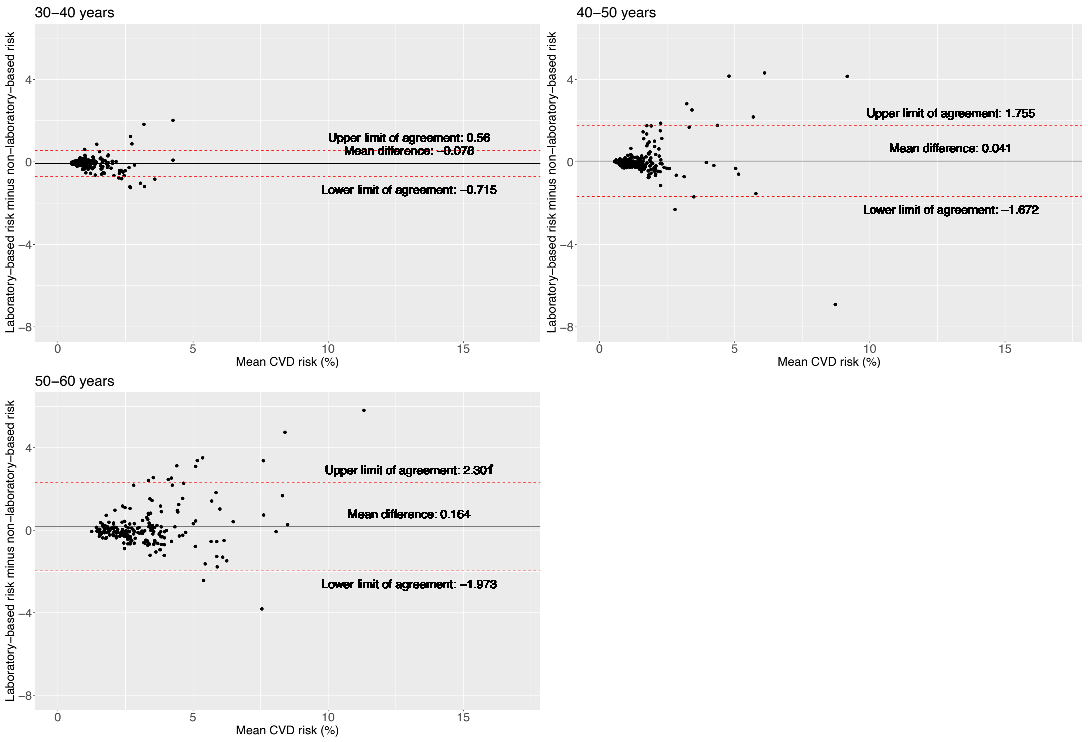

Supplementary Figure 5. Sensitivity Analysis: Bland Altman plots showing agreement between laboratory and non-laboratory-based risk scores according to body mass index categories.

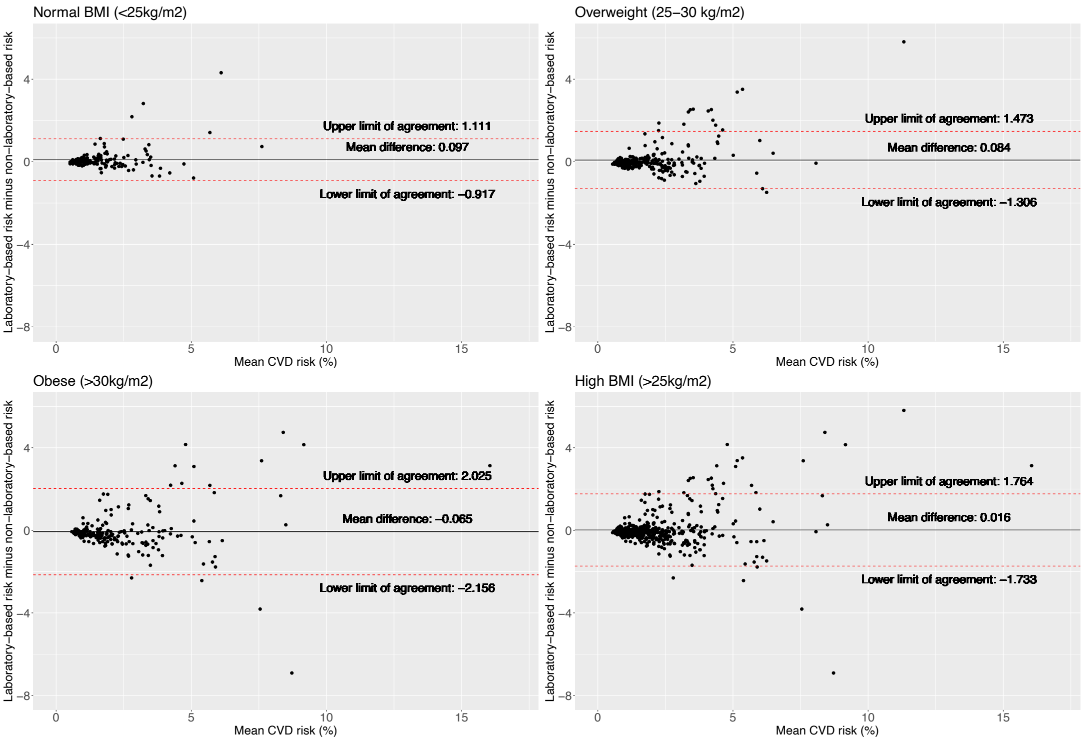

Supplementary Figure 6. Sensitivity Analysis: Bland Altman plots showing agreement between laboratory and non-laboratory-based risk scores according to smoking status.

Guzman-Vilca WC, *et al. BMJ Open* 2022; 12:e063289. doi: 10.1136/bmjopen-2022-063289

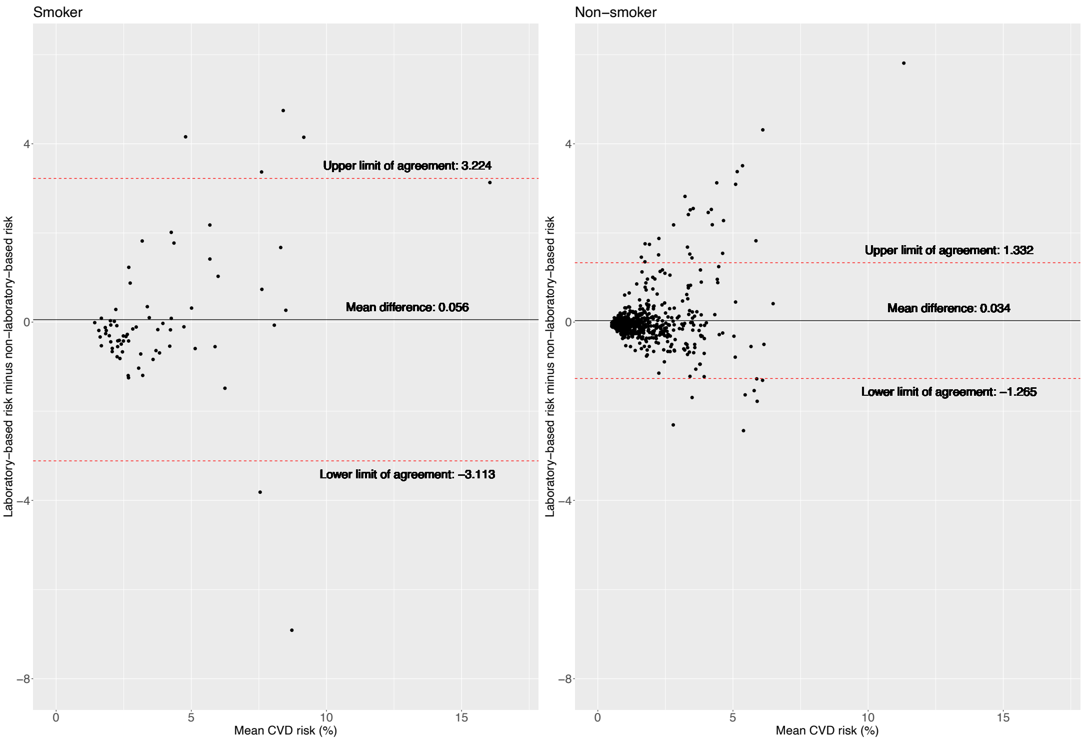

Supplementary Figure 7. Sensitivity Analysis: Bland Altman plots showing agreement between laboratory and non-laboratory-based risk scores according to self-reported diabetes status.

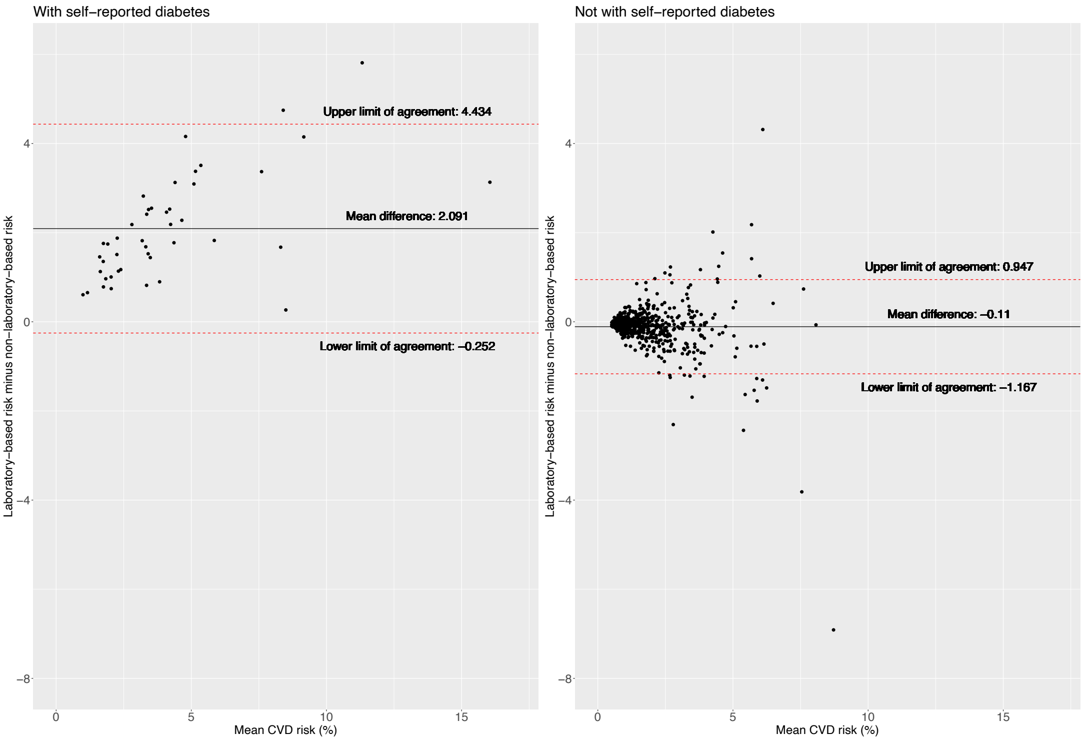

Supplement: Supplementary data [file bmjopen-2022-063289supp001.pdf]
