# Supplementary material for: Agreement between the laboratory-based and non-laboratory-based WHO cardiovascular risk charts: a cross-sectional analysis of a national health survey in Peru
Source: BMJ Open. 2022 Nov 7;12(11):e063289. doi: 10.1136/bmjopen-2022-063289 (PMC9644334; doi:10.1136/bmjopen-2022-063289)
Supplement: Author's manuscript [file bmjopen-2022-063289.draft_revisions.pdf]

BMJ Open is committed to open peer review. As part of this commitment we make the peer review history of every article we publish publicly available.

When an article is published we post the peer reviewers' comments and the authors' responses online. We also post the versions of the paper that were used during peer review. These are the versions that the peer review comments apply to.

The versions of the paper that follow are the versions that were submitted during the peer review process. They are not the versions of record or the final published versions. They should not be cited or distributed as the published version of this manuscript.

BMJ Open is an open access journal and the full, final, typeset and author-corrected version of record of the manuscript is available on our site with no access controls, subscription charges or pay-per-view fees (http://bmjopen.bmj.com).

If you have any questions on BMJ Open's open peer review process please email info.bmjopen@bmj.com

BMJ Open

Agreement between the laboratory- and non-laboratory-based 2019 WHO cardiovascular risk charts in Peru

Journal:	*BMJ Open*
Manuscript ID	bmjopen-2022-063289
Article Type:	Original research
Date Submitted by the Author:	28-Mar-2022
Complete List of Authors:	Guzman-Vilca, Wilmer Cristobal; Universidad Peruana Cayetano Heredia, School of Medicine "Alberto Hurtado"; Universidad Peruana Cayetano Heredia, CRONICAS Centre of Excellence in Chronic Diseases<br>Quispe-Villegas, Gustavo A.; Universidad Peruana Cayetano Heredia, School of Medicine "Alberto Hurtado"<br>Váscones Román, Fritz Fidel; Universidad Peruana Cayetano Heredia, School of Medicine "Alberto Hurtado"<br>Bernabe-Ortiz, Antonio; Universidad Peruana Cayetano Heredia, CRONICAS Centre of Excellence in Chronic Diseases; Universidad Cientifica del Sur, School of Medicine<br>Carrillo-Larco, Rodrigo; Universidad Peruana Cayetano Heredia, CRONICAS Centre of Excellence in Chronic Diseases; Imperial College London, Department of Epidemiology and Biostatistics
Keywords:	Cardiac Epidemiology < CARDIOLOGY, Adult cardiology < CARDIOLOGY, EPIDEMIOLOGY

SCHOLARONE™
Manuscripts

I, the Submitting Author has the right to grant and does grant on behalf of all authors of the Work (as defined in the below author licence), an exclusive licence and/or a non-exclusive licence for contributions from authors who are: i) UK Crown employees; ii) where BMJ has agreed a CC-BY licence shall apply, and/or iii) in accordance with the terms applicable for US Federal Government officers or employees acting as part of their official duties; on a worldwide, perpetual, irrevocable, royalty-free basis to BMJ Publishing Group Ltd ("BMJ") its licensees and where the relevant Journal is co-owned by BMJ to the co-owners of the Journal, to publish the Work in this journal and any other BMJ products and to exploit all rights, as set out in our licence.

The Submitting Author accepts and understands that any supply made under these terms is made by BMJ to the Submitting Author unless you are acting as an employee on behalf of your employer or a postgraduate student of an affiliated institution which is paying any applicable article publishing charge ("APC") for Open Access articles. Where the Submitting Author wishes to make the Work available on an Open Access basis (and intends to pay the relevant APC), the terms of reuse of such Open Access shall be governed by a Creative Commons licence – details of these licences and which Creative Commons licence will apply to this Work are set out in our licence referred to above.

Other than as permitted in any relevant BMJ Author's Self Archiving Policies, I confirm this Work has not been accepted for publication elsewhere, is not being considered for publication elsewhere and does not duplicate material already published. I confirm all authors consent to publication of this Work and authorise the granting of this licence.

For peer review only

Agreement between the laboratory- and non-laboratory-based 2019 WHO cardiovascular risk charts in Peru

Wilmer Cristobal Guzman-Vilca [1,2,3]

Gustavo A. Quispe-Villegas [1,3]

Fritz Fidel Vascones-Roman [1.2,3]

Antonio Bernabe-Ortiz [2,5]

Rodrigo M. Carrillo-Larco [2,4]

1. School of Medicine “Alberto Hurtado”, Universidad Peruana Cayetano Heredia, Lima, Peru.
2. CRONICAS Centre of Excellence in Chronic Diseases, Universidad Peruana Cayetano Heredia, Lima, Peru.
3. Sociedad Científica de Estudiantes de Medicina Cayetano Heredia (SOCEMCH), Universidad Peruana Cayetano Heredia, Lima, Peru.
4. Department of Epidemiology and Biostatistics, School of Public Health, Imperial College London, London, UK.
5. Universidad Cientifica del Sur, Lima, Peru.

Correspondence author:

Rodrigo M Carrillo-Larco, MD

Department of Epidemiology and Biostatistics

School of Public Health

Imperial College London

rcarrill@ic.ac.uk

For peer review only

ABSTRACT

Objective: To determine the agreement between the cardiovascular disease (CVD) risk predictions computed with the World Health Organization (WHO) non-laboratory-based model and laboratory-based model in a nationally-representative sample of Peruvian adults.

Design: Cross-sectional analysis of a national health survey.

Methods: Absolute CVD risk was computed with the 2019 WHO laboratory- and non-laboratory-based models. The risk predictions from both models were compared with Bland Altman plots, Lin's concordance coefficient correlation (LCCC) and kappa statistics, stratified by sex, age, body mass index categories, smoking and diabetes status.

Results: 663 people aged 30-59 years were included in the analysis. Overall, there were no substantial differences between the mean CVD risk computed with the laboratory-based model 2.0% (95% CI: 1.8%; 2.2%) and the non-laboratory-based model 2.0% (95% CI: 1.8%; 2.1%). In the Bland Altman plots, the limits of agreement were the widest among people with diabetes (-0.21; 4.37) compared with people without diabetes (-1.17; 0.95). The lowest agreement as per the LCCC was also seen in people with diabetes (0.74 (95% CI: 0.63; 0.82)), the same was observed with the kappa statistic (kappa=0.36). In general, agreement between the scores was appropriate in terms of clinical significance.

Conclusions: The absolute cardiovascular predicted risk was similar between the laboratory-based and non-laboratory-based 2019 WHO cardiovascular risk models. The agreement between these models was less clear in people with cardiovascular risk factors: obesity, smokers and people with diabetes.

Key words: cardiovascular diseases; risk assessment; health metrics

For peer review only

STRENGTHS AND LIMITATIONS OF THIS STUDY

- This analysis provided the first evidence of the agreement between the 2019 WHO CVD risk laboratory-based and non-laboratory-based models.
- We leveraged on the most recent nationally representative survey that included blood biomarkers in Peru.
- Our study population was young, mostly women, and with overall low absolute cardiovascular predicted risk. No one in the study population had an absolute cardiovascular risk ≥20%.
- We assumed all participants were free of CVD to use the 2019 WHO CVD risk score, as information regarding history of cardiovascular events was not reported.

INTRODUCTION

Cardiovascular diseases (CVD) are the main cause of death globally.[1] In 2019, CVD caused ~18.5 million deaths in adults, representing 36% of all global deaths.[2,3] Furthermore, CVD impose a huge burden in low- and middle-income countries (LMICs),[4] where deaths from CVD occur at younger ages compared to high-income countries (HICs).[1] However, CVD can be prevented and managed through a combination of population- and individual-level interventions;[5] for the latter, the identification of individuals at high cardiovascular risk is a cornerstone in the prevention of CVD. In this line, the World Health Organization (WHO) and the Pan American Health Organization (PAHO),[6] alongside several clinical guidelines,[7–9] recommend CVD risk stratification with CVD risk prediction models to inform evidence-based treatment.

CVD risk prediction models identify people who would benefit the most of preventive interventions (e.g., statin therapy).[10] Although there are several CVD risk prediction models,[11] these were mostly developed in HICs limiting their application in LMICs where they would need recalibration to deliver accurate predictions to guide treatment allocation. To overcome this limitation, the WHO convened a global effort to derive, calibrate and validate new CVD risk prediction models for all world regions.[12] Two WHO CVD risk prediction models were developed: a laboratory-based model and a non-laboratory-based model. Because laboratory biomarkers (e.g., total cholesterol)

For peer review only

may not be available in primary health care centres in LMICs,[13] the non-laboratory-based model arises as a handy tool for clinicians in LMICs. Nonetheless, evidence regarding the agreement between the WHO laboratory-based and non-laboratory-based models in countries from Latin America is missing.[12] Whether the WHO non-laboratory-based model delivers predictions similar to those of the WHO laboratory-based model remains unknown in Latin America. However, clinicians need this evidence to inform their choice (non-laboratory-based vs laboratory-based model), and to interpret the results of the non-laboratory-based model under the assumption that the laboratory-based model is the gold standard.[12] To provide this evidence for practitioners in Peru, we determined the agreement between the risk predictions computed with the non-laboratory-based model and laboratory-based model in a nationally-representative sample of Peruvian adults.

METHODS

Data sources

This is a cross-sectional study of a national survey conducted by the National Centre for Food and Nutrition (CENAN, for its acronym in Spanish) of Peru. CENAN's survey was conducted between 2017-2018 on a nationally-representative sample of Peruvian adults aged between 18-59 years.[14] Of note, this is the most recent nationally-representative survey conducted in Peru that included blood biomarkers (e.g., lipid profile).

Study population

We analysed a complete-case sample regarding all the laboratory- and office-based 2019 ten-year WHO CVD risk score variables (see Variables section). We studied men and women aged between 30-59 years. The younger age limit was decided because CVD risk models are not recommended in younger individuals; the older age limit was decided because of the survey design. A flowchart of data cleaning is shown in Supplementary figure 1. We did not apply other selection criteria.

Although the 2019 ten-year WHO CVD risk models were developed for people aged 40-80 years,[12] for people aged <40 years we assumed they had 40 years for the absolute CVD risk

For peer review only

computation. This is consistent with the Package of essential noncommunicable (PEN) disease interventions for primary health care in low-resource settings,[15] and was also done in a recent global work.[16]

Variables

We calculated the ten-year CVD risk at the individual-level following the laboratory- and non-laboratory-based 2019 ten-year WHO CVD risk model.[12] We used the *whocvdrisk c*ommand in STATA, which was developed by the authors of the 2019 ten-year WHO CVD risk charts.[17] For the laboratory-based model, scores were calculated based on: age (years), current smoking status (yes/no), systolic blood pressure (SBP, mmHg), history of self-reported diabetes diagnosis (yes/no), and total cholesterol (mmol/L).[12] For the non-laboratory-based model, we used age (years), current smoking status (yes/no), systolic BP (mmHg) and body mass index (BMI, kg/m^2).[12]

The CENAN's survey collected anthropometrics and three BP measurements which were taken by trained fieldworkers following a standard protocol.[14] We computed BMI using measured weight (kg) divided by the square of height (meters); for descriptive purposes, we classified BMI in three levels: normal weight (BMI <25 kg/m^2), overweight (BMI ≥25-29.9 kg/m^2), and obesity (BMI ≥30 kg/m^2). BMI records outside the range 10-80 kg/m^2 were discarded. As the third BP measurement was only available in few participants (<2% of the initial sample), we used the second SBP measurement only (i.e., the first and third SBP records were discarded in the main analysis). Of note, there were no substantial differences between the first and second SBP records (Supplementary table 1); nonetheless, we performed a sensitivity analysis using the mean SBP of first and second SBP records. We discarded any SBP records outside the range 70-270 mmHg.

For people who self-reported being under antihypertensive treatment, we used the pre-treatment SBP; this is consistent with the PEN protocol and with a previous global work.[15,16]. Pre-treatment SBP was computed as: pre-treatment systolic blood pressure = (current systolic blood pressure-6.3)/0.9.[18] Conversely, for those not taking antihypertensive treatment, we used the recorded SBP as was.

For peer review only

We defined current smoker with one question coded as no versus yes: *do you currently smoke any tobacco product such as cigarettes, cigars or pipes?* Self-reported information about a prior history of diabetes was assessed by a question also coded as no versus yes: *have you ever been told by a physician or another healthcare worker that you have high blood sugar or diabetes?*

Total cholesterol was obtained via enzymatic colorimetric method.[14] Because CENAN's survey data had total cholesterol in mg/dl, these values were divided by 38.67 to obtain total cholesterol in mmol/L.

For peer review only

Statistical analysis

We determined the agreement between the absolute CVD risk predicted with the WHO laboratory- and non-laboratory-based models following three methods: Bland Altman plots, Lin's concordance coefficient correlation (LCCC), and kappa statistic. We considered the absolute CVD risk as a continuous variable, and the agreement between both models was examined using Bland Altman plots and the LCCC. Furthermore, we considered the CVD risk as a categorical variable, and divided into three groups: <5%, 5-9%, 10-19%; because there were no observations in the high-risk category (CVD risk ≥20%), agreement was not examined in this group. For these categories, we evaluated the agreement using the kappa statistic. For the Bland Altman plots, LCCC, and the kappa statistic, results were stratified by sex, 10-year age groups, BMI categories, smoking status, self-reported diabetes diagnosis and urban/rural location.

In the Bland Altman plots, the risk difference between the laboratory- and non-laboratory-based absolute cardiovascular predicted risk was plotted on the vertical axis, and the mean of both scores on the horizontal axis.[19] As the true risk of CVDs at the individual-level is uncertain, the mean of both scores is the best available estimate.[19,20] The 95% of the limit of agreement was represented by the mean difference of both scores ± two standard deviations; this limit provides an interval in which 95% of the differences between both scores would be expected to lie.[19] The LCCC between the laboratory- and non-laboratory-based absolute cardiovascular predicted risks was also evaluated. The agreement based on the LCCC ranges between -1 and 1, with 1

suggesting a perfect agreement. The categorical agreement was evaluated using the Kappa statistic. Kappa <0 indicated less than chance agreement, and values 0.01–0.20, 0.21–0.40, 0.41–0.60, 0.61–0.80, and 0.81–0.99 represented slight, fair, moderate, substantial, and almost perfect agreement, respectively.[21]

The datasets we used and the analysis code in R (version 4.0.3) and STATA (version 17.0, College Station, Texas 77845, USA) are available as Supplementary Files 1-2. Population characteristics along with their 95% confidence interval (95% CI) were summarized accounting for the complex survey design of the CENAN's survey.[14]

Ethics

This study used de-identified nationally-representative survey data that can be requested from the CENAN. We did not seek approval by an Ethics Committee.

Patient and public involvement

No patient involved.

Role of the funding source

The funder had no role in the study design, analysis, interpretation or decision to publish. The authors are collectively responsible for the accuracy of the data. The arguments and opinions in this work are those of the authors alone, and do not represent the position of the institutions to which they belong.

RESULTS

Our pooled dataset included 663 participants (Supplementary figure 1). The mean age was 44.0 years (95% CI: 43.2; 44.7) and the proportion of men was 41.5%. The mean SBP was 108.9 mmHg (95% CI: 107.5; 110.3 mmHg) and the mean BMI was 28.8 kg/m^2 (95% CI: 28.3; 29.2 kg/m^2). The proportion of people with overweight was 41.0% (95% CI: 36.6; 45.6 %), whereas 35.9% (95% CI: (31.6; 40.5%) of the population were obese. The mean total cholesterol was 4.9

For peer review only

mmol/L (95% CI: 4.8; 5.0 mmol/L), 11.7% (95% CI: 8.8; 15.3%) of the population were smokers and 7.1% (95% CI: 5.0%; 9.8%) had diabetes (Table 1).

Table 1. Weighted distribution of the predictors in the 2019 WHO CVD risk models, overall and by sex.

	Total	Men	Women
**Sample size**	663	280	383
**Age (mean and 95% CI, years)**	44 (43.2-44.7)	44.2 (43-45.4)	43.8 (42.9-44.7)
**Proportion of people aged 30-39 years (95% CI, %)**	35.8 (31.6-40.3)	37.2 (31-43.9)	34.8 (29.4-40.7)
**Proportion of people aged 40-49 years (95% CI, %)**	34 (29.8-38.4)	29.5 (24-35.7)	37.1 (31.5-43.1)
**Proportion of people aged 50-59 years (95% CI, %)**	30.2 (26.3-34.5)	33.3 (27.1-40)	28 (23.5-33.1)
**Systolic blood pressure (mean and 95% CI, mmHg)**	108.9 (107.5-110.3)	116.1 (113.8-118.4)	103.8 (102.3-105.3)
**Diastolic blood pressure (mean and 95% CI, mmHg)**	71.9 (71-72.8)	74.6 (73.1-76.1)	70 (69-71)
**Body mass index (mean and 95% CI, kg/m2)**	28.8 (28.3-29.2)	28.3 (27.6-29)	29.1 (28.5-29.7)
**Proportion of people with normal weight (95% CI, %)**	23.1 (19.5-27.2)	24.5 (19.5-30.4)	22.1 (17.2-27.9)
**Proportion of people with overweight (95% CI, %)**	41 (36.6-45.6)	45.3 (38.9-52)	37.9 (31.9-44.3)
**Proportion of people with obesity (95% CI, %)**	35.9 (31.6-40.5)	30.1 (24.1-36.9)	40 (34-46.2)
**Total cholesterol (mean and 95% CI, mmol/L)**	4.9 (4.8-5)	4.8 (4.6-5)	4.9 (4.8-5.1)
**Proportion of smokers (95% CI, %)**	11.7 (8.8-15.3)	21.1 (15.8-27.6)	5 (3-8.3)
**Proportion of people with diabetes (95% CI, %)**	7.1 (5-9.8)	7.8 (4.7-12.6)	6.5 (4.3-9.9)
**Laboratory-based CVD risk score (mean and 95% CI, %)**	2 (1.8-2.2)	2.6 (2.3-2.9)	1.6 (1.4-1.7)
**Non-laboratory-based CVD risk score (mean and 95% CI, %)**	2 (1.8-2.1)	2.7 (2.4-3)	1.5 (1.4-1.6)

review only

Absolute cardiovascular risk according to the 2019 WHO cardiovascular risk models

Overall, there were no substantial differences between the mean absolute cardiovascular risk computed with the laboratory-based and non-laboratory-based models (Table 1). The mean absolute cardiovascular risk was 2.0% (95% CI: 1.8%; 2.2%) according to the laboratory-based model, and 2.0% (95% CI: 1.8%; 2.1%) according to the non-laboratory-based model. In both models, the mean absolute cardiovascular risk was higher in men than women. The sensitivity analysis (using the mean of two SBP records) yielded the same findings in the overall sample: 2.0% (95% CI: 1.8; 2.2%) in the laboratory-based-model and 2.0 (95% CI: 1.8; 2.1%) in the non-laboratory-based model.

Mean difference between risk predictions

Overall, the mean difference between the laboratory-based and the non-laboratory-based models was 0.03% (95% CI: -0.03%; 0.10%). According to sex, the mean difference between models was -0.02% (95% CI: -0.14%; 0.09%) in men, and 0.08% (95% CI: 0.01%; 0.15%) in women. According to age, the mean difference between models was -0.07% (95% CI: -0.12%; 0.04%) in people aged 30-39 years, 0.04% (95% CI: -0.08%; 0.15%) in people aged 40-49 years, and 0.17% (95% CI: 0.02%; 0.32%) in people aged 50-59 years. Stratified by BMI categories, the mean difference was 0.10% (95% CI: 0.02%; 0.18%) in people with normal weight, 0.08% (95% CI: 0.00%; 0.17%) in people with overweight, and -0.07% (95% CI: -0.21%; 0.07%) in people with obesity. The mean difference was 0.05% (95% CI: -0.34%; 0.44%) in smokers and 0.03% (95% CI: -0.02%; 0.09%) in non-smokers. According to self-reported diabetes status, mean difference was 2.08% (95% CI: 1.73%; 2.44%) in people with self-reported diabetes, and -0.11% (95% CI: -0.15%; 0.07%) in people without self-reported diabetes. The sensitivity analysis provided similar results across all variables; for example, the largest mean difference was also observed in people with self-reported diabetes (2.09% (95% CI: 1.73; 2.45%) (Supplementary figure 6).

Bland-Altman plots and limits of agreement.

The limit of agreement was slightly narrower for women (-1.23; 1.39) compared to men (-1.93; 1.89) (Figure 1). The limit of agreement widened with older ages and higher BMI levels; for

For peer review only - http://bmjopen.bmj.com/site/about/guidelines.xhtml

example, the limit of agreement was narrower in people aged 30-39 years (-0.72; 0.56) compared to those aged 50-59 years (-1.96; 2.29) (Figure 2), and for people with normal weight (-0.90; 1.09) compared to those with obesity (-2.15; 2.02) (Figure 3). According to smoking and self-reported diabetes status, the limit of agreement was wider in those who had the condition. For example, the limit of agreement in smokers (-3.13; 3.24) was wider compared to non-smokers (-1.25; 1.32) (Figure 4). Similarly, in people with self-reported diabetes (-0.21; 4.37) the limit of agreement was wider compared to people without self-reported diabetes (-1.17; 0.95) (Figure 5). Notably, the sensitivity analysis resulted in similar limits of agreement in all variables (Supplementary figures 2-6).

Agreement by Lin's concordance coefficient correlation

The overall agreement between scores as per the LCCC was 0.87 (95% CI: 0.85; 0.89), and it was virtually the same in men (0.87 (95% CI 0.84; 0.89)) and women (0.85 (95% CI 0.82; 0.87)). Across age groups, the highest agreement was seen in the 30-39 age group (0.87 (95% CI 0.84; 0.9)). Across BMI categories, it was the normal BMI category which had the highest agreement (0.90 (95% CI 0.87; 0.92)). Overall, the lowest agreement values were seen across smokers (0.81 (95% CI 0.72; 0.88)), those aged 40-49 years (0.74 (95% CI 0.67; 0.79)), and those with self-reported diabetes (0.74 (95% CI 0.63; 0.82)) (Table 2). Similar results were seen in the sensitivity analysis (Supplementary table 2).

Table 2. Lin's concordance coefficient correlation showing agreement between laboratory and non-laboratory-based risk models according to the predictors in the 2019 WHO CVD risk models and urban/rural location

Variables	Categories	Lin's concordance coefficient correlation (95% CI)
Sex	Men	0.87 (0.84 - 0.89)
	Women	0.85 (0.82 - 0.87)
Age (years)	30-39	0.87 (0.84 - 0.9)
	40-49	0.74 (0.67 - 0.79)
	50-59	0.83 (0.78 - 0.86)
Body mass index category	Normal	0.9 (0.87 - 0.92)

For peer review only - http://bmjopen.bmj.com/site/about/guidelines.xhtml

	**Overweight**	0.87 (0.85 - 0.9)
	**Obese**	0.86 (0.82 - 0.89)
**Smoking status**	**Smoker**	0.81 (0.72 - 0.88)
	**Non-smoker**	0.86 (0.84 - 0.88)
**Diabetes status**	**With self−reported diabetes**	0.74 (0.63 - 0.82)
	**Not with self−reported diabetes**	0.91 (0.9 - 0.92)
**Urban or rural**	**Urban**	0.88 (0.85 - 0.9)
	**Rural**	0.85 (0.82 - 0.88)

Categorical agreement

In the overall population, there was a slightly larger number of people categorized as having an absolute CVD risk of 5-9% and 10-19% with the laboratory-based-model compared with the non-laboratory-based model (Supplementary table 3). For example, the laboratory-based model categorized 37 people in the 5-9% CVD risk category, whereas the non-laboratory-based model categorized 26 people. Overall, the agreement between risk categories was substantial (kappa=0.62), and it was better for men (kappa=0.70) compared to women (kappa=0.44) (Supplementary table 4). Of note, the lowest agreement between risk categories was observed among people with self-reported diabetes (kappa=0.36): out of 14 people with self-reported diabetes in the 5-9% CVD risk category following the laboratory-based-model, 4 were placed in the same category following the non-laboratory-based model, as the rest were placed in the 0-5% CVD risk category. The categorical agreement according to all variables is presented in Supplementary tables 3-9.

DISCUSSION

Main findings

In this work, we evaluated the agreement between the CVD risk estimates predicted with the 2019 WHO ten-year laboratory-based and non-laboratory-based models in a nationally-representative sample of Peruvian adults. The mean absolute predicted CVD risk according to both models in the general population was virtually the same. In addition, we found that the limits of agreement between both models increased with a higher CVD risk; for instance, the limits of agreement were

For peer review only - http://bmjopen.bmj.com/site/about/guidelines.xhtml

wider in smokers and people with self-reported diabetes. We observed good agreement between the laboratory-based and non-laboratory-based models in terms of clinical significance.[20,22] These findings suggest that, in a population with a similar profile to that in this study, practitioners could use either the laboratory-based or non-laboratory-based models. Although the difference is very small, careful interpretation could be needed for people with cardiovascular risk factors: obesity, smokers and people with self-reported diabetes, amongst whom the difference was slightly larger than in their peers without these risk factors.

Public health implications

We provided insights about the applicability of the 2019 WHO non-laboratory-based model in the Peruvian population. This evidence is relevant in terms of clinical practice and public health in Peru and other similar countries (e.g., Andean Latin America), because it informs whether the predictions based on the laboratory-based and non-laboratory-based CVD risk models are equivalent. If so, either of these models could be used without substantial bias, hence supporting the use of the non-laboratory-based model when blood biomarkers are not available.

Our results suggest that the agreement between the laboratory-based and non-laboratory-based models was appropriate among Peruvians with low CVD risk and younger than 60 years. In other words, our results suggest that the laboratory-based and non-laboratory-based models provide similar predictions and may therefore be used interchangeably as needed, though the profile of our study population ought to be considered when extrating or implementing our findings into clinical practice and public health. Of note, the small number of participants in some variables of interest (e.g., only 44 participants had self-reported diabetes) could have explained the broader limits of agreement in our results. Further studies should include a larger number of participants to further confirm whether limits of agreement are wider according to smoking and diabetes status.

Research in context

The study most comparable to ours evaluated the agreement between the Framingham 10-year CVD risk laboratory and non-laboratory models on a population aged 40-75 years in southern

For peer review only

For peer review only - http://bmjopen.bmj.com/site/about/guidelines.xhtml

Iran.[22] They found the mean CVD risk following the non-laboratory-based model (9.4%) was higher than the laboratory-based model (6.7%).[22] Additionally, their limits of agreement between both Framingham models in people <60 years old were wider compared to ours in both men (-1.9-1.9 by our estimates versus -2.5%-8.9% by Rezaei et al.[22]) and women (-1.2-1.4 by our estimates versus -2.3%-4.6% by Rezaei et al.[22]). This could be explained by the fact that Rezaei et al.[22] included an older population, which tend to have a higher absolute CVD risk. As limits of agreement between two models tend to widen with higher CVD risk,[20,22] our limits of agreement would presumably be wider if we had studied a similar population to that of the work by Rezaei et al.[22] The differences between our results could be further explained by the CVD risk score herein used. We used the 2019 WHO CVD risk models,[12] whereas Rezaei et al. used the Framingham risk scores. The Framingham risk score was developed for a more specific population (Caucasians),[23] yet the 2019 WHO CVD risk model was developed for a broader population (i.e., those living in LMICs).[12]

The agreement between the 2019 WHO laboratory- and non-laboratory-based model was also explored in the global work convened by the WHO.[12] They applied the two models to WHO STEPS surveys, and compared the proportion of people categorized at different levels of predicted CVD risk.[12] Overall, they found moderate agreement between both models, and their discrepancy was attributed to poor performance of the non-laboratory-based model in people with diabetes.[12] This finding is consistent with our results because we found the widest limits of agreement, the lowest LCCC, and the lowest categorical agreement in people with self-reported diabetes. When possible, it would seem reasonable to use the laboratory-based model in those whose have diabetes.

Strengths and limitations

To the best of our knowledge, we provided the first evidence of the agreement between the 2019 WHO CVD risk laboratory-based and non-laboratory-based models; furthermore, we leveraged on a nationally-representative survey. Nonetheless, this study has also limitations. First, our study population was young (30-59 years), mostly women (58%), and with overall low absolute cardiovascular predicted risk. This led to the observation that no one in the study population had

For peer review only - http://bmjopen.bmj.com/site/about/guidelines.xhtml

an absolute cardiovascular risk ≥20%. Thus, we could only draw conclusions for people within the low and medium CVD risk range, and with a similar demographic and risk factor profile. We acknowledge that further subgroup analysis could be relevant, for example by diabetes status. However, because of data availability and the reduced number of observations in some groups, this subgroup analyses would be impossible to conduct. Future work in Peru and Latin America should verify our results with a larger, older and more diverse population. Second, as CENAN's survey did not include history of cardiovascular events, we assumed all participants were free of CVD to use the 2019 WHO CVD risk score. This approach could have led to higher absolute cardiovascular risk because people who have had a cardiovascular event (e.g., myocardial infarction) are at higher risk of another cardiovascular event. Third, we only used one blood pressure record (the second SBP measurement); ideally, we should have used the average of multiple records having discarded the very first measurement. This approach was not possible with the available data. We performed a sensitivity analysis using the mean SBP of the first and second records; this revealed virtually the same results as in our main analysis in which only the second SBP record was used.

Conclusions

The absolute cardiovascular predicted risk was similar between the laboratory-based and non-laboratory based 2019 WHO cardiovascular risk models. The agreement between these models was less clear in people with cardiovascular risk factors: obesity, smokers and people with diabetes. While universal health coverage momentum helps to have laboratory tests in (most) primary care facilities to use the laboratory-based model, it seems reasonable to use the non-laboratory-based model for primary prevention of CVD following the risk stratification approach.

For peer review only - http://bmjopen.bmj.com/site/about/guidelines.xhtml

FIGURES

Figure 1. Bland Altman plots showing agreement between laboratory and non-laboratory-based risk scores according to sex.

Figure 2. Bland Altman plots showing agreement between laboratory and non-laboratory-based risk scores according to age groups.

Figure 3. Bland Altman plots showing agreement between laboratory and non-laboratory-based risk scores according to body mass index categories

Figure 4. Bland Altman plots showing agreement between laboratory and non-laboratory-based risk scores according to smoking status.

Figure 5. Bland Altman plots showing agreement between laboratory and non-laboratory-based risk scores according to self-reported diabetes status.

For peer review only

For peer review only - http://bmjopen.bmj.com/site/about/guidelines.xhtml

Conflict of interest: None to declare.

Funding: RMC-L is supported by a Wellcome Trust International Training Fellowship (214185/Z/18/Z).

Contributors: RMC-L conceived the idea with WCG-V. WCG-V conducted the analysis with support from GAQ-V and FFV-R. WCG-V wrote the first draft of the manuscript with support from GAQ-V, FFV-R, AB-O and RMC-L. All authors provided relevant scientific contribution. All authors approved the submitted version.

Data sharing statement: Datasets and analysis code in Supplementary Files 1-2.

Word count: 3,260

For peer review only

REFERENCES

1. World Health Organization. Cardiovascular diseases (CVDs) [Internet]. [cited 2021 Aug 15]. Available from: https://www.who.int/news-room/fact-sheets/detail/cardiovascular-diseases-(cvds)
2. GBD 2019 Diseases and Injuries Collaborators. Global burden of 369 diseases and injuries in 204 countries and territories, 1990-2019: a systematic analysis for the Global Burden of Disease Study 2019. Lancet. 2020 Oct 17;396(10258):1204–22.
3. GBD Results Tool | GHDx [Internet]. Available from: http://ghdx.healthdata.org/gbd-results-tool
4. Prabhakaran D, Anand S, Watkins D, Gaziano T, Wu Y, Mbanya JC, et al. Cardiovascular, respiratory, and related disorders: key messages from Disease Control Priorities, 3rd edition. Lancet. 2018 Mar 24;391(10126):1224–36.
5. Arnett DK, Blumenthal RS, Albert MA, Buroker AB, Goldberger ZD, Hahn EJ, et al. 2019 ACC/AHA Guideline on the Primary Prevention of Cardiovascular Disease: Executive Summary: A Report of the American College of Cardiology/American Heart Association Task Force on Clinical Practice Guidelines. Circulation. 2019 Sep 10;140(11):e563–95.
6. PAHO/WHO | Pan American Health Organization. HEARTS in the Americas - [Internet]. [cited 2021 Aug 16]. Available from: https://www.paho.org/en/hearts-americas
7. Whelton PK, Carey RM, Aronow WS, Casey DE, Collins KJ, Dennison Himmelfarb C, et al. 2017 ACC/AHA/AAPA/ABC/ACPM/AGS/APhA/ASH/ASPC/NMA/PCNA Guideline for the Prevention, Detection, Evaluation, and Management of High Blood Pressure in Adults: Executive Summary: A Report of the American College of Cardiology/American Heart Association Task Force on Clinical Practice Guidelines. Hypertension. 2018 Jun;71(6):1269–324.
8. Williams B, Mancia G, Spiering W, Agabiti Rosei E, Azizi M, Burnier M, et al. 2018 ESC/ESH Guidelines for the management of arterial hypertension: The Task Force for the management of arterial hypertension of the European Society of Cardiology and the European Society of Hypertension: The Task Force for the management of arterial hypertension of the European Society of Cardiology and the European Society of Hypertension. J Hypertens. 2018 Oct;36(10):1953–2041.
9. NICE National Institute for Health and Care Excellence. Hypertension in adults: diagnosis and management | Guidance [Internet]. NICE; 2019 [cited 2021 Aug 16]. Available from: https://www.nice.org.uk/guidance/ng136
10. Karmali KN, Persell SD, Perel P, Lloyd-Jones DM, Berendsen MA, Huffman MD. Risk scoring for the primary prevention of cardiovascular disease. Cochrane Database Syst Rev. 2017 Mar 14;3:CD006887.
11. Damen JAAG, Hooft L, Schuit E, Debray TPA, Collins GS, Tzoulaki I, et al. Prediction models for cardiovascular disease risk in the general population: systematic review. BMJ. 2016 May 16;353:i2416.
12. WHO CVD Risk Chart Working Group. World Health Organization cardiovascular disease risk charts: revised models to estimate risk in 21 global regions. Lancet Glob Health. 2019 Oct;7(10):e1332–45.
13. Wilson ML, Fleming KA, Kuti MA, Looi LM, Lago N, Ru K. Access to pathology and laboratory medicine services: a crucial gap. Lancet. 2018 May 12;391(10133):1927–38.
14. Centro Nacional de Alimentación y Nutrición. ESTADO NUTRICIONAL EN ADULTOS DE 18 A 59 AÑOS, PERÚ: 2017 - 2018 [Internet]. 2021. Available from: https://web.ins.gob.pe/sites/default/files/Archivos/cenan/van/sala_nutricional/sala_3/2021/Informe%20Tecnico-%20Estado%20nutricional%20en%20adultos%20de%2018%20a%2059%20a%C3%B1os%2CVIANEV%202017-2018.pdf
15. Package of essential noncommunicable (PEN) disease interventions for primary health care in low-resource setting. WHO PEN Protocol 1 Prevention of HEart Attacks, Stroke and Kidney Disease through Integrated Management of Diabetes and Hypertension [Internet]. Available from: https://www.who.int/ncds/management/Protocol1_HeartAttack_strokes_kidneyDisease.pdf?ua=1
16. Peiris D, Ghosh A, Manne-Goehler J, Jaacks LM, Theilmann M, Marcus ME, et al. Cardiovascular disease risk profile and management practices in 45 low-income and middle-income countries: A cross-sectional study of nationally representative individual-level survey

For peer review only - http://bmjopen.bmj.com/site/about/guidelines.xhtml

data. PLoS Med. 2021 Mar;18(3):e1003485.
17. University of Cambridge. Cardiovascular Epidemiology Unit. Programs [Internet]. Cardiovascular Epidemiology Unit. [cited 2021 Aug 15]. Available from: https://www.phpc.cam.ac.uk/ceu/population-resources-and-tools/programs/
18. Law MR, Morris JK, Wald NJ. Use of blood pressure lowering drugs in the prevention of cardiovascular disease: meta-analysis of 147 randomised trials in the context of expectations from prospective epidemiological studies. BMJ. 2009 May 19;338:b1665.
19. Bland JM, Altman DG. Statistical methods for assessing agreement between two methods of clinical measurement. Lancet. 1986 Feb 8;1(8476):307–10.
20. Jones CA, Ross L, Surani N, Dharamshi N, Karmali K. Framingham ten-year general cardiovascular disease risk: agreement between BMI-based and cholesterol-based estimates in a South Asian convenience sample. PLoS One. 2015;10(3):e0119183.
21. Viera AJ, Garrett JM. Understanding interobserver agreement: the kappa statistic. Fam Med. 2005 May;37(5):360–3.
22. Rezaei F, Seif M, Gandomkar A, Fattahi MR, Hasanzadeh J. Agreement between laboratory-based and non-laboratory-based Framingham risk score in Southern Iran. Sci Rep. 2021 May 24;11(1):10767.
23. D'Agostino RB, Vasan RS, Pencina MJ, Wolf PA, Cobain M, Massaro JM, et al. General cardiovascular risk profile for use in primary care: the Framingham Heart Study. Circulation. 2008 Feb 12;117(6):743–53.

For peer review only

For peer review only - http://bmjopen.bmj.com/site/about/guidelines.xhtml

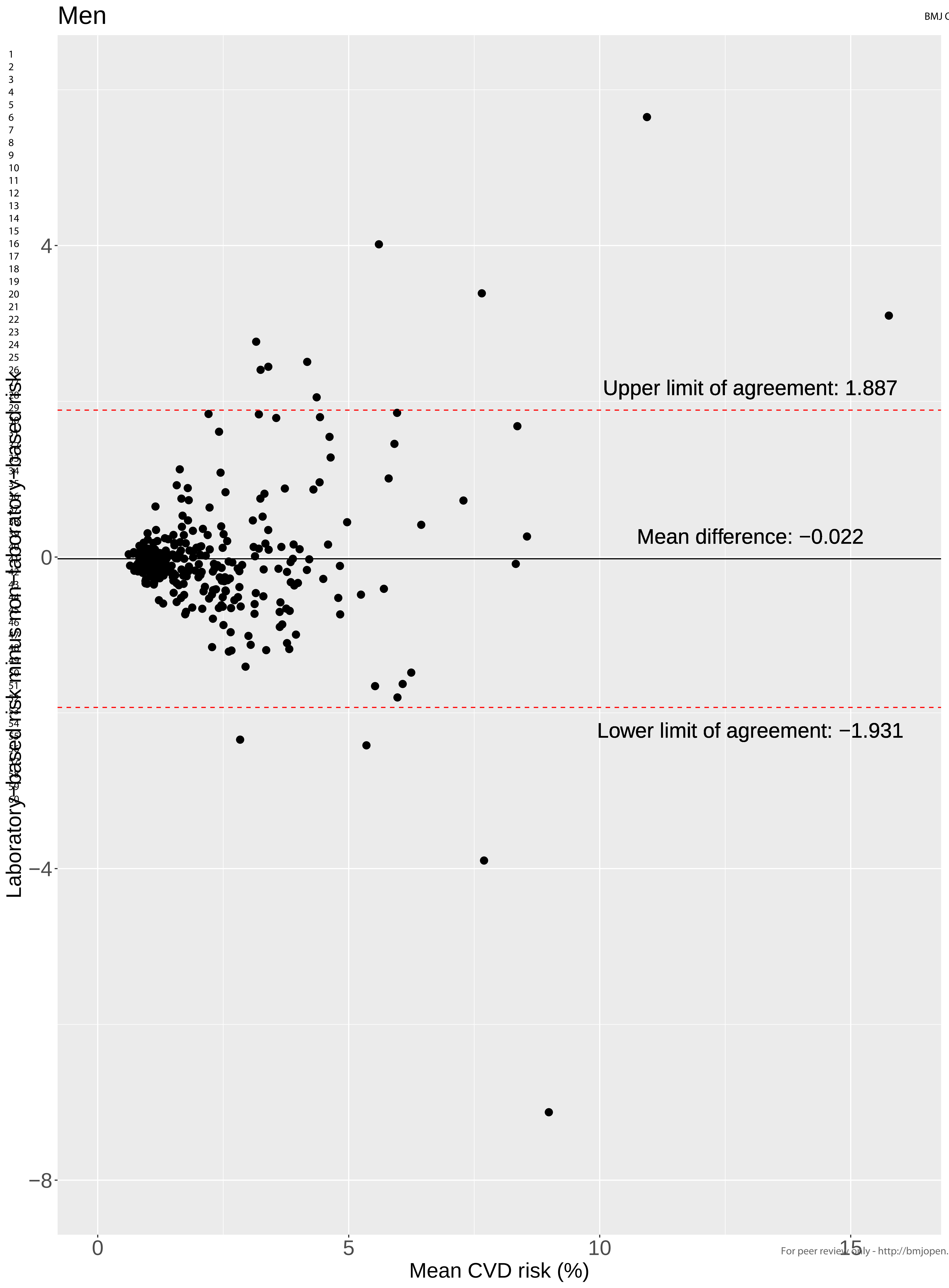
Men
Upper limit of agreement: 1.887
Mean difference: −0.022
Lower limit of agreement: −1.931
Laboratory−based risk minus non−laboratory−based risk
Mean CVD risk (%)
4
0
−4
−8
0
5
10
15

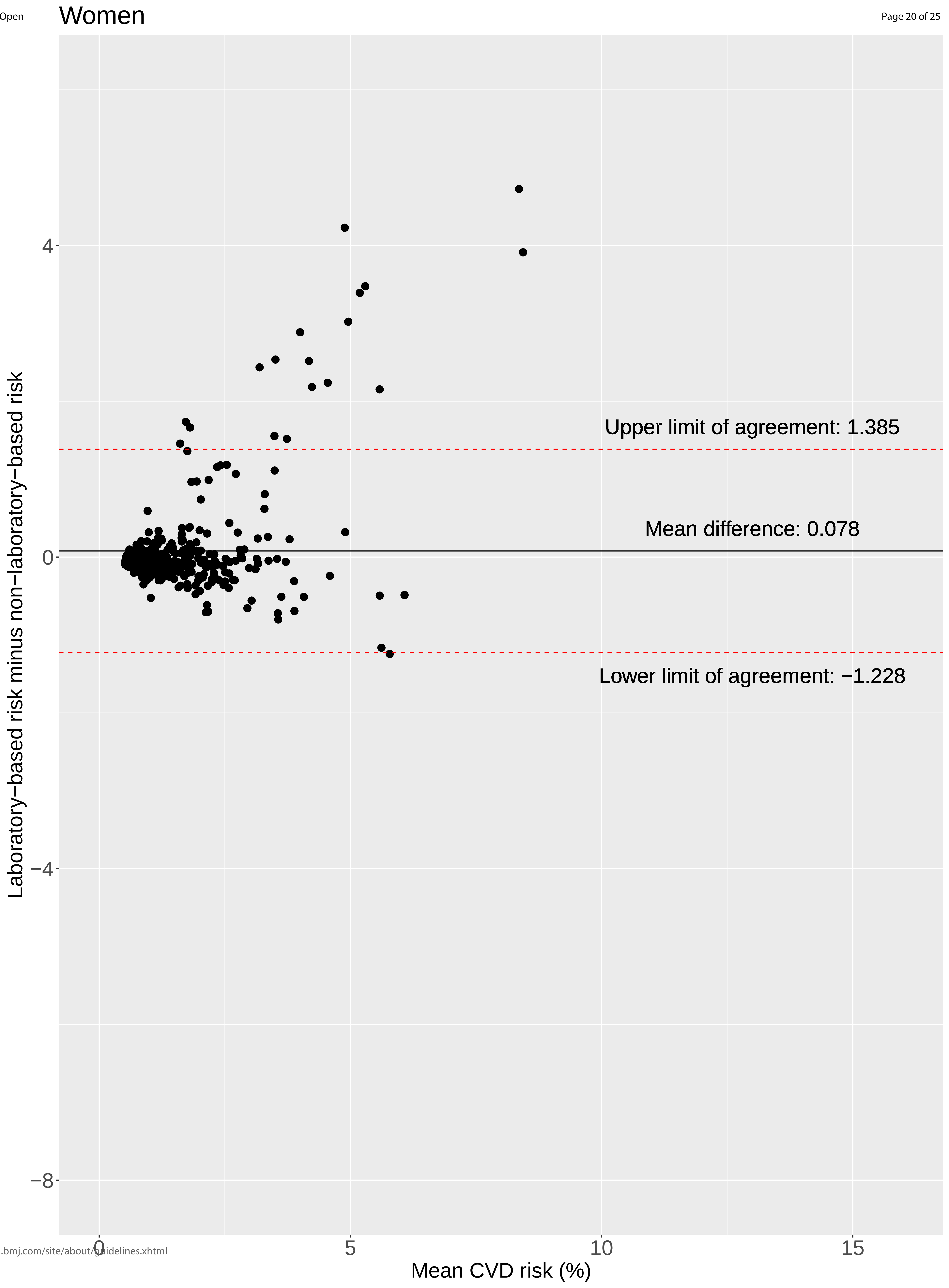
Women
Upper limit of agreement: 1.385
Mean difference: 0.078
Lower limit of agreement: −1.228
Laboratory−based risk minus non−laboratory−based risk
Mean CVD risk (%)
4
0
−4
−8
0
5
10
15

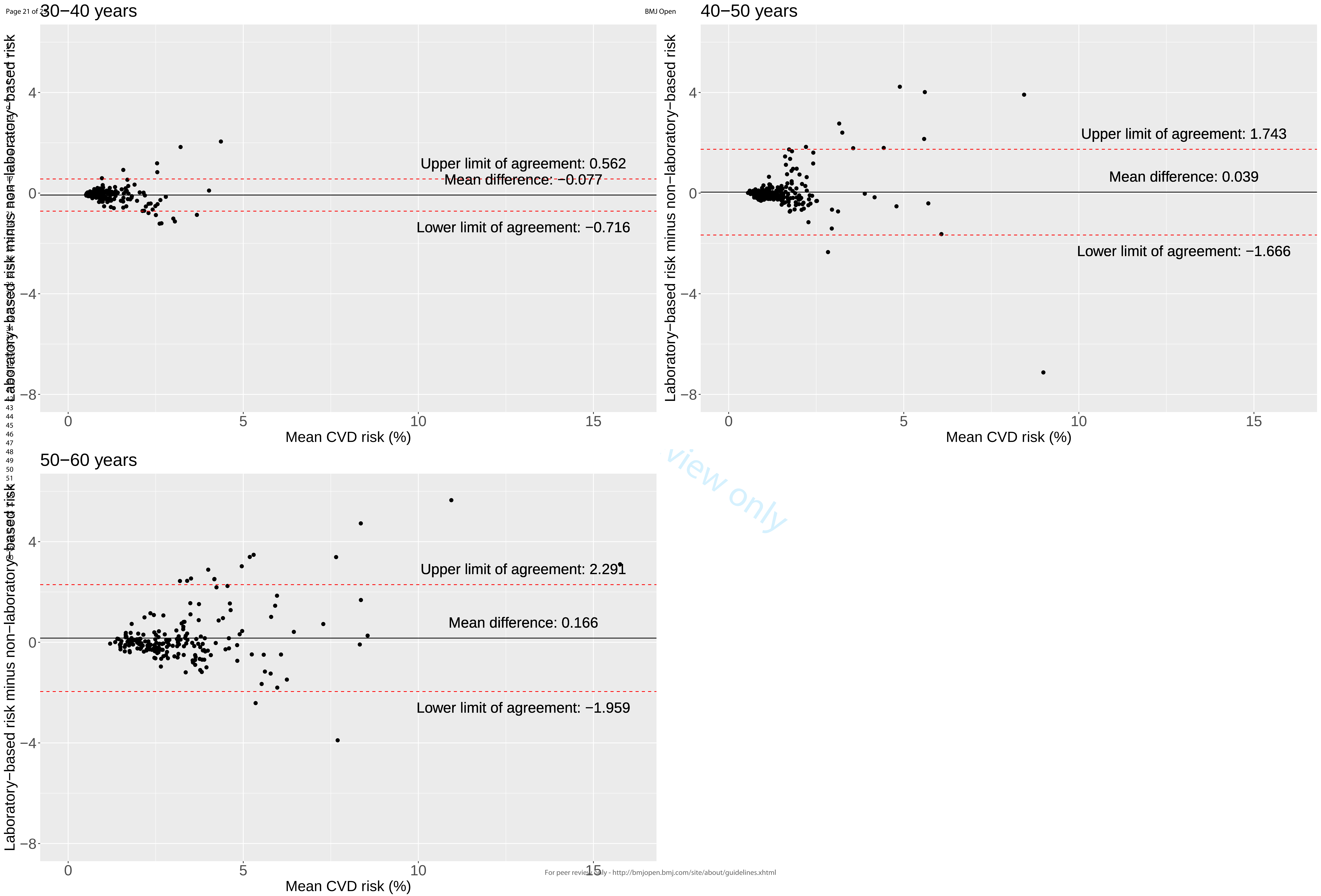
30−40 years
Laboratory−based risk minus non−laboratory−based risk
Upper limit of agreement: 0.562
Mean difference: −0.077
Lower limit of agreement: −0.716
Mean CVD risk (%)
40−50 years
Laboratory−based risk minus non−laboratory−based risk
Upper limit of agreement: 1.743
Mean difference: 0.039
Lower limit of agreement: −1.666
Mean CVD risk (%)
50−60 years
Laboratory−based risk minus non−laboratory−based risk
Upper limit of agreement: 2.291
Mean difference: 0.166
Lower limit of agreement: −1.959
Mean CVD risk (%)

view only

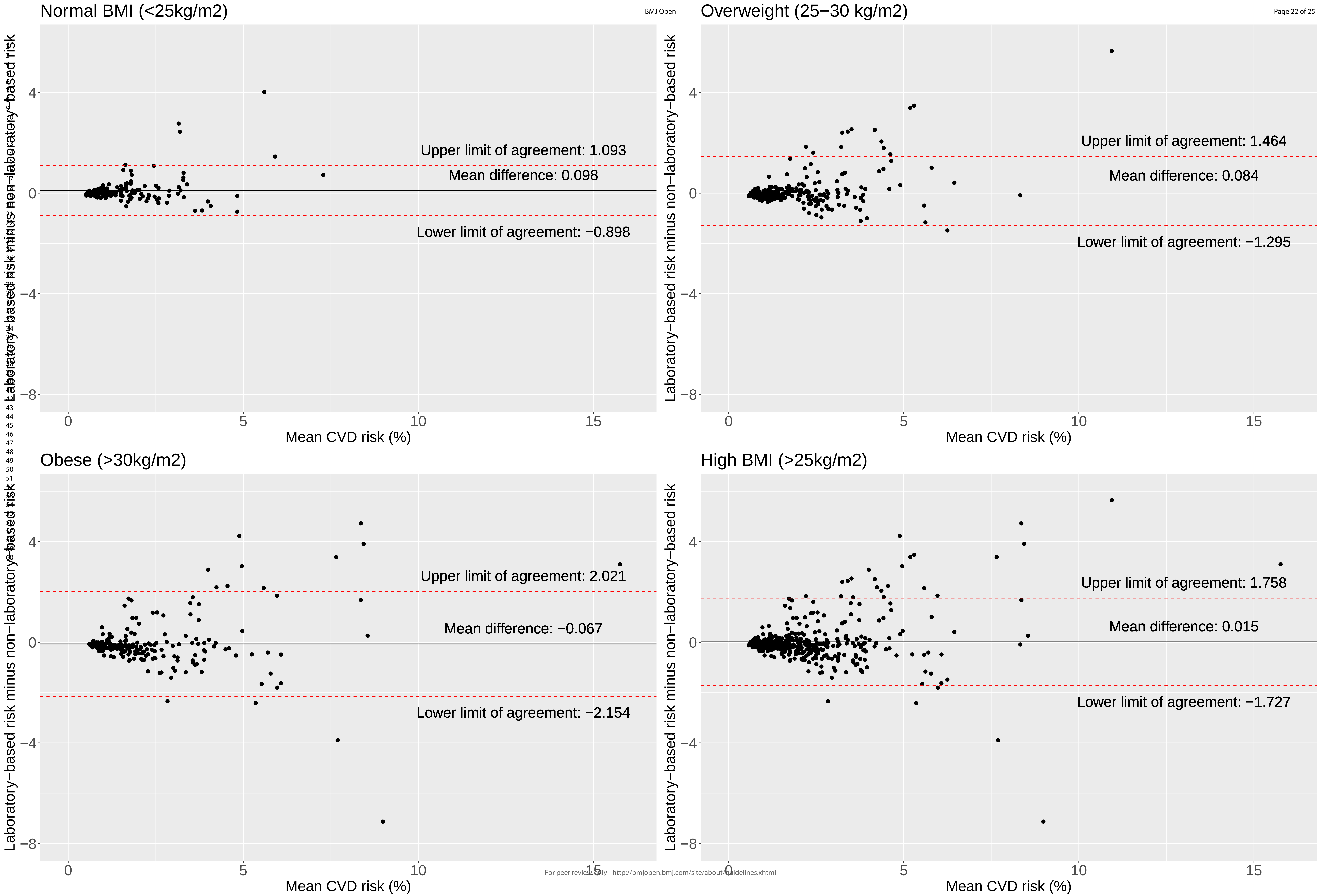
Normal BMI (<25kg/m2)
Upper limit of agreement: 1.093
Mean difference: 0.098
Lower limit of agreement: −0.898
Laboratory−based risk minus non−laboratory−based risk
Mean CVD risk (%)
Overweight (25−30 kg/m2)
Upper limit of agreement: 1.464
Mean difference: 0.084
Lower limit of agreement: −1.295
Obese (>30kg/m2)
Upper limit of agreement: 2.021
Mean difference: −0.067
Lower limit of agreement: −2.154
High BMI (>25kg/m2)
Upper limit of agreement: 1.758
Mean difference: 0.015
Lower limit of agreement: −1.727

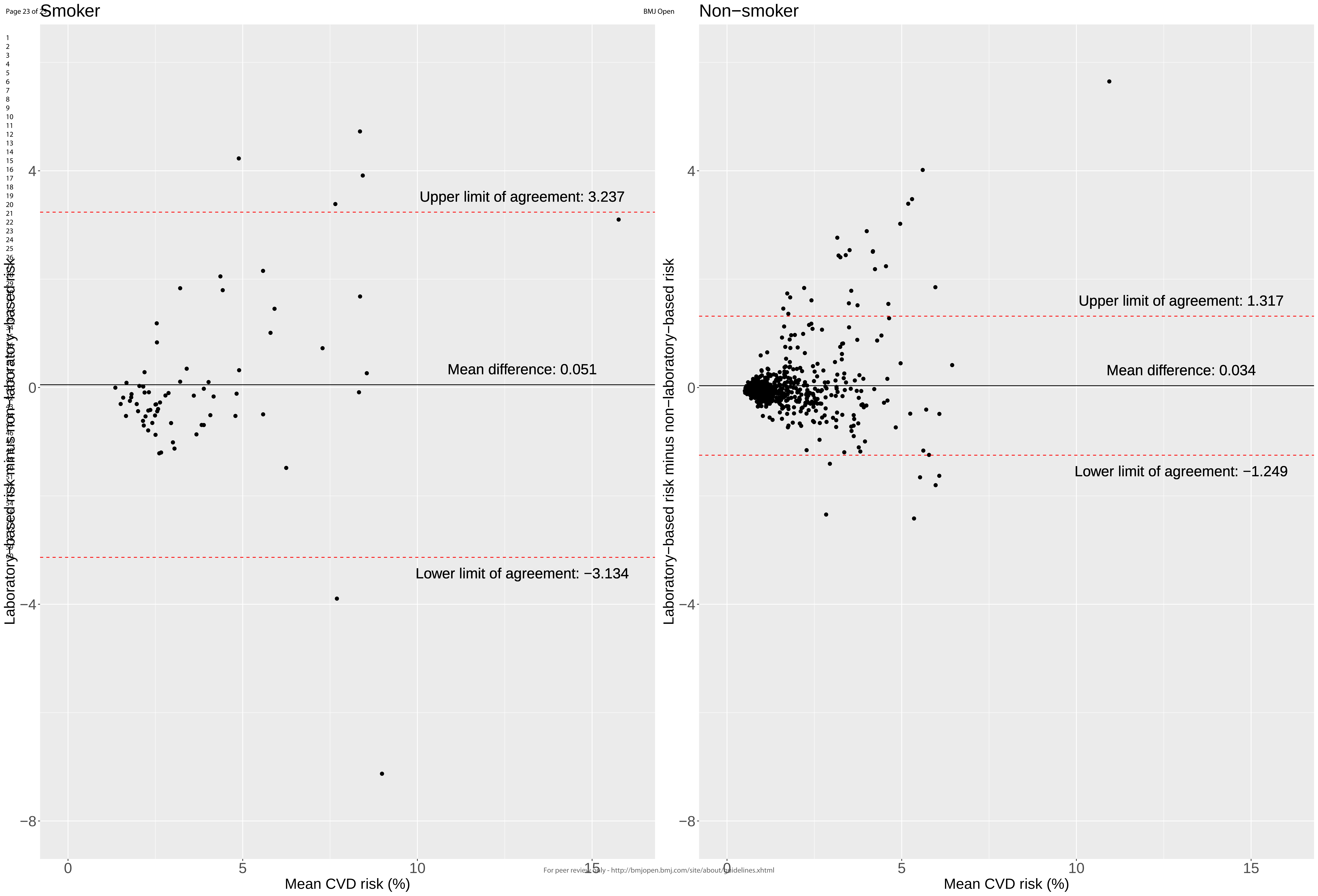
Smoker
Non−smoker
Upper limit of agreement: 3.237
Mean difference: 0.051
Lower limit of agreement: −3.134
Upper limit of agreement: 1.317
Mean difference: 0.034
Lower limit of agreement: −1.249
Laboratory−based risk minus non−laboratory−based risk
Mean CVD risk (%)
4
0
−4
−8
0
5
10
15

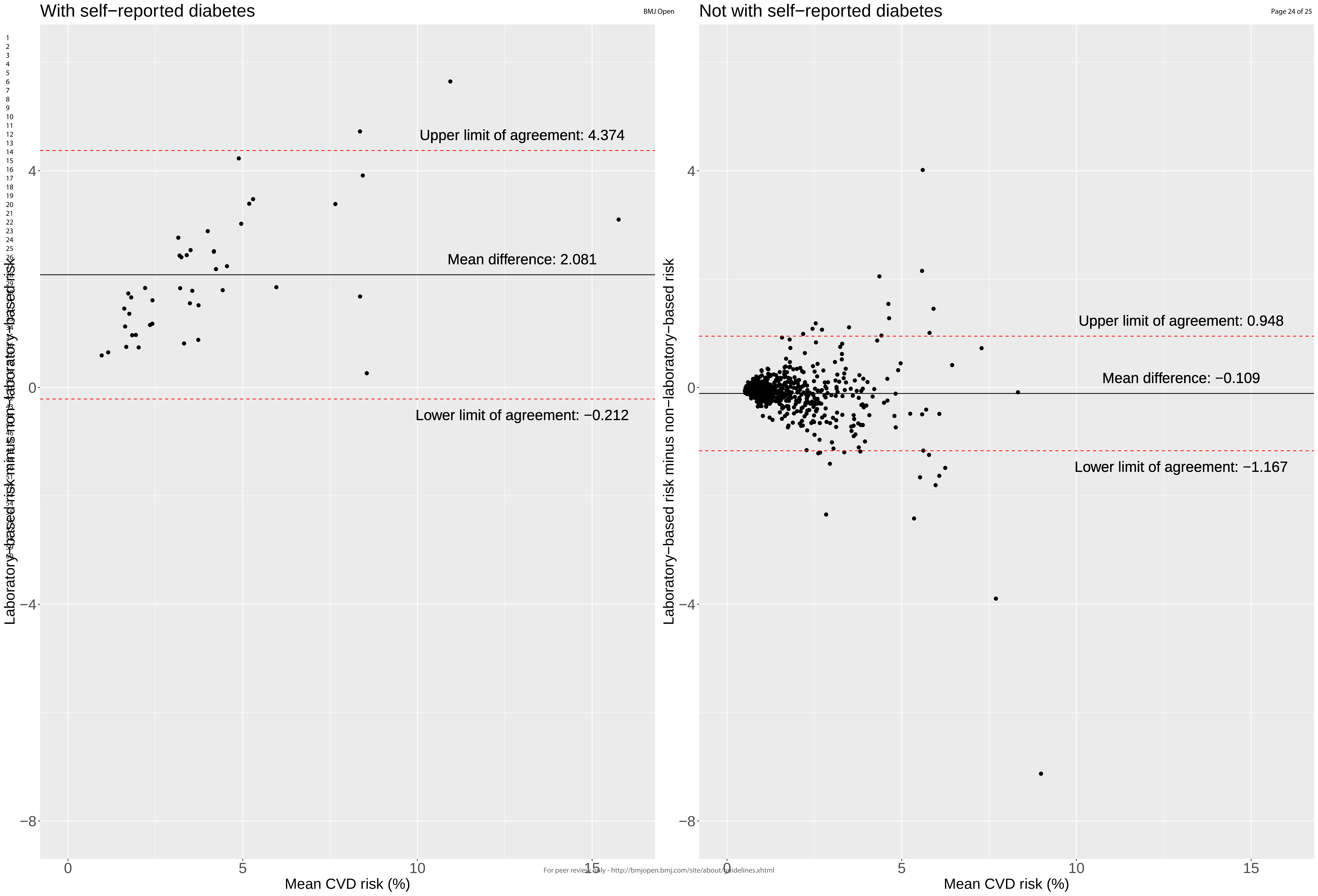
With self−reported diabetes
Upper limit of agreement: 4.374
Mean difference: 2.081
Lower limit of agreement: −0.212
Laboratory−based risk minus non−laboratory−based risk
Mean CVD risk (%)
4
0
−4
−8
0
5
10
15
Not with self−reported diabetes
Upper limit of agreement: 0.948
Mean difference: −0.109
Lower limit of agreement: −1.167
Laboratory−based risk minus non−laboratory−based risk
Mean CVD risk (%)
4
0
−4
−8
0
5
10
15

STROBE Statement—Checklist of items that should be included in reports of ***cross-sectional studies***

	Item No	Recommendation	Page No
**Title and abstract**	1	(*a*) Indicate the study's design with a commonly used term in the title or the abstract	3
		(*b*) Provide in the abstract an informative and balanced summary of what was done and what was found	3
**Introduction**			
Background/rationale	2	Explain the scientific background and rationale for the investigation being reported	4
Objectives	3	State specific objectives, including any prespecified hypotheses	5
**Methods**			
Study design	4	Present key elements of study design early in the paper	5
Setting	5	Describe the setting, locations, and relevant dates, including periods of recruitment, exposure, follow-up, and data collection	5-6
Participants	6	(*a*) Give the eligibility criteria, and the sources and methods of selection of participants	5-6
Variables	7	Clearly define all outcomes, exposures, predictors, potential confounders, and effect modifiers. Give diagnostic criteria, if applicable	6-7
Data sources/ measurement	8*	For each variable of interest, give sources of data and details of methods of assessment (measurement). Describe comparability of assessment methods if there is more than one group	*6-7*
Bias	9	Describe any efforts to address potential sources of bias	6
Study size	10	Explain how the study size was arrived at	5-6
Quantitative variables	11	Explain how quantitative variables were handled in the analyses. If applicable, describe which groupings were chosen and why	6-7
Statistical methods	12	(*a*) Describe all statistical methods, including those used to control for confounding	7-8
		(*b*) Describe any methods used to examine subgroups and interactions	7-8
		(*c*) Explain how missing data were addressed	5-6
		(*d*) If applicable, describe analytical methods taking account of sampling strategy	8
		(*e*) Describe any sensitivity analyses	6
**Results**			
Participants	13*	(a) Report numbers of individuals at each stage of study—eg numbers potentially eligible, examined for eligibility, confirmed eligible, included in the study, completing follow-up, and analysed	8
		(b) Give reasons for non-participation at each stage	Supp Fig 1
		(c) Consider use of a flow diagram	5
Descriptive data	14*	(a) Give characteristics of study participants (eg demographic, clinical, social) and information on exposures and potential confounders	8
		(b) Indicate number of participants with missing data for each variable of interest	Supp Fig 1
Outcome data	15*	Report numbers of outcome events or summary measures	

For peer review only - http://bmjopen.bmj.com/site/about/guidelines.xhtml

Main results	16	(*a*) Give unadjusted estimates and, if applicable, confounder-adjusted estimates and their precision (eg, 95% confidence interval). Make clear which confounders were adjusted for and why they were included	8-10
		(*b*) Report category boundaries when continuous variables were categorized	8-10
		(*c*) If relevant, consider translating estimates of relative risk into absolute risk for a meaningful time period	-
Other analyses	17	Report other analyses done—eg analyses of subgroups and interactions, and sensitivity analyses	10
**Discussion**			
Key results	18	Summarise key results with reference to study objectives	11
Limitations	19	Discuss limitations of the study, taking into account sources of potential bias or imprecision. Discuss both direction and magnitude of any potential bias	13
Interpretation	20	Give a cautious overall interpretation of results considering objectives, limitations, multiplicity of analyses, results from similar studies, and other relevant evidence	11-12
Generalisability	21	Discuss the generalisability (external validity) of the study results	11-12
**Other information**			
Funding	22	Give the source of funding and the role of the funders for the present study and, if applicable, for the original study on which the present article is based	2

*Give information separately for exposed and unexposed groups.

Note: An Explanation and Elaboration article discusses each checklist item and gives methodological background and published examples of transparent reporting. The STROBE checklist is best used in conjunction with this article (freely available on the Web sites of PLoS Medicine at http://www.plosmedicine.org/, Annals of Internal Medicine at http://www.annals.org/, and Epidemiology at http://www.epidem.com/). Information on the STROBE Initiative is available at www.strobe-statement.org.

For peer review only

BMJ Open

Agreement between the laboratory- and non-laboratory-based 2019 WHO cardiovascular risk charts in Peru

Journal:	*BMJ Open*
Manuscript ID	bmjopen-2022-063289.R1
Article Type:	Original research
Date Submitted by the Author:	08-Sep-2022
Complete List of Authors:	Guzman-Vilca, Wilmer Cristobal; Universidad Peruana Cayetano Heredia, School of Medicine "Alberto Hurtado"; Universidad Peruana Cayetano Heredia, CRONICAS Centre of Excellence in Chronic Diseases<br>Quispe-Villegas, Gustavo A.; Universidad Peruana Cayetano Heredia, School of Medicine "Alberto Hurtado"<br>Váscones Román, Fritz Fidel; Universidad Peruana Cayetano Heredia, School of Medicine "Alberto Hurtado"<br>Bernabe-Ortiz, Antonio; Universidad Peruana Cayetano Heredia, CRONICAS Centre of Excellence in Chronic Diseases; Universidad Cientifica del Sur, School of Medicine<br>Carrillo-Larco, Rodrigo; Universidad Peruana Cayetano Heredia, CRONICAS Centre of Excellence in Chronic Diseases; Imperial College London, Department of Epidemiology and Biostatistics
**Primary Subject Heading**:	Cardiovascular medicine
Secondary Subject Heading:	Cardiovascular medicine
Keywords:	Cardiac Epidemiology < CARDIOLOGY, Adult cardiology < CARDIOLOGY, EPIDEMIOLOGY

SCHOLARONE™
Manuscripts

I, the Submitting Author has the right to grant and does grant on behalf of all authors of the Work (as defined in the below author licence), an exclusive licence and/or a non-exclusive licence for contributions from authors who are: i) UK Crown employees; ii) where BMJ has agreed a CC-BY licence shall apply, and/or iii) in accordance with the terms applicable for US Federal Government officers or employees acting as part of their official duties; on a worldwide, perpetual, irrevocable, royalty-free basis to BMJ Publishing Group Ltd ("BMJ") its licensees and where the relevant Journal is co-owned by BMJ to the co-owners of the Journal, to publish the Work in this journal and any other BMJ products and to exploit all rights, as set out in our licence.

The Submitting Author accepts and understands that any supply made under these terms is made by BMJ to the Submitting Author unless you are acting as an employee on behalf of your employer or a postgraduate student of an affiliated institution which is paying any applicable article publishing charge ("APC") for Open Access articles. Where the Submitting Author wishes to make the Work available on an Open Access basis (and intends to pay the relevant APC), the terms of reuse of such Open Access shall be governed by a Creative Commons licence – details of these licences and which Creative Commons licence will apply to this Work are set out in our licence referred to above.

Other than as permitted in any relevant BMJ Author's Self Archiving Policies, I confirm this Work has not been accepted for publication elsewhere, is not being considered for publication elsewhere and does not duplicate material already published. I confirm all authors consent to publication of this Work and authorise the granting of this licence.

For peer review only

Agreement between the laboratory- and non-laboratory-based 2019 WHO cardiovascular risk charts in Peru

Wilmer Cristobal Guzman-Vilca [1,2,3]

Gustavo A. Quispe-Villegas [1,3]

Fritz Fidel Vascones-Roman [1.2,3]

Antonio Bernabe-Ortiz [2,5]

Rodrigo M. Carrillo-Larco [2,4]

1. School of Medicine "Alberto Hurtado", Universidad Peruana Cayetano Heredia, Lima, Peru.
2. CRONICAS Centre of Excellence in Chronic Diseases, Universidad Peruana Cayetano Heredia, Lima, Peru.
3. Sociedad Científica de Estudiantes de Medicina Cayetano Heredia (SOCEMCH), Universidad Peruana Cayetano Heredia, Lima, Peru.
4. Department of Epidemiology and Biostatistics, School of Public Health, Imperial College London, London, UK.
5. Universidad Cientifica del Sur, Lima, Peru.

Correspondence author:

Rodrigo M Carrillo-Larco, MD

Department of Epidemiology and Biostatistics

School of Public Health

Imperial College London

rcarrill@ic.ac.uk

For peer review only

ABSTRACT

Objective: To determine the agreement between the cardiovascular disease (CVD) risk predictions computed with the World Health Organization (WHO) non-laboratory-based model and laboratory-based model in a nationally-representative sample of Peruvian adults.

Design: Cross-sectional analysis of a national health survey.

Methods: Absolute CVD risk was computed with the 2019 WHO laboratory- and non-laboratory-based models. The risk predictions from both models were compared with Bland Altman plots, Lin's concordance coefficient correlation (LCCC) and kappa statistics, stratified by sex, age, body mass index categories, smoking and diabetes status.

Results: 663 people aged 30-59 years were included in the analysis. Overall, there were no substantial differences between the mean CVD risk computed with the laboratory-based model 2.0% (95% CI: 1.8%; 2.2%) and the non-laboratory-based model 2.0% (95% CI: 1.8%; 2.1%). In the Bland Altman plots, the limits of agreement were the widest among people with diabetes (-0.21; 4.37) compared with people without diabetes (-1.17; 0.95). The lowest agreement as per the LCCC was also seen in people with diabetes (0.74 (95% CI: 0.63; 0.82)), the same was observed with the kappa statistic (kappa=0.36). In general, agreement between the scores was appropriate in terms of clinical significance.

Conclusions: The absolute cardiovascular predicted risk was similar between the laboratory-based and non-laboratory-based 2019 WHO cardiovascular risk models. Pending validation from longitudinal studies, the non-laboratory-based model (instead of the laboratory-based) could be used when assessing CVD risk in Peruvian population.

Key words: cardiovascular diseases; risk assessment; health metrics

For peer review only

STRENGTHS AND LIMITATIONS OF THIS STUDY

- This analysis provided the first evidence of the agreement between the 2019 WHO CVD risk laboratory-based and non-laboratory-based models.
- We leveraged on the most recent nationally representative survey that included blood biomarkers in Peru.
- Our study population was young, mostly women, and with overall low absolute cardiovascular predicted risk. No one in the study population had an absolute cardiovascular risk ≥20%.
- We assumed all participants were free of CVD to use the 2019 WHO CVD risk score, as information regarding history of cardiovascular events was not reported.

For peer review only

INTRODUCTION

Cardiovascular diseases (CVD) are the main cause of death globally.[1] In 2019, CVD caused ~18.5 million deaths in adults, representing 36% of all global deaths.[2,3] Furthermore, CVD impose a huge burden in low- and middle-income countries (LMICs),[4] where deaths from CVD occur at younger ages compared to high-income countries (HICs).[1] However, CVD can be prevented and managed through a combination of population- and individual-level interventions;[5] for the latter, the identification of individuals at high cardiovascular risk is a cornerstone in the prevention of CVD. In this line, the World Health Organization (WHO) and the Pan American Health Organization (PAHO),[6] alongside several clinical guidelines,[7–9] recommend CVD risk stratification with CVD risk prediction models to inform evidence-based treatment.

CVD risk prediction models identify people who would benefit the most of preventive interventions (e.g., statin therapy).[10] Although there are several CVD risk prediction models,[11] these were mostly developed in HICs limiting their application in LMICs where they would need recalibration to deliver accurate predictions to guide treatment allocation. To overcome this limitation, the WHO convened a global effort to derive, calibrate and validate new CVD risk prediction models for all world regions.[12] Two WHO CVD risk prediction models were developed: a laboratory-based

model and a non-laboratory-based model. Because laboratory biomarkers (e.g., total cholesterol) may not be available in primary health care centres in LMICs limiting the use of laboratory-based CVD risk prediction models,[13] the non-laboratory-based model arises as a handy tool for clinicians in LMICs. Similarly, CVD risk prediction models are used to monitor the prevalence of high CVD risk and treatment coverage (i.e., people at high CVD receiving treatment).[14] In this context, countries conducting national or large population-based health surveys without lipid biomarkers, could benefit from the non-laboratory-based models. Peru, for example, does not have regular national health surveys including total cholesterol, but Peru has a yearly national health survey including anthropometrics, blood pressure, and health questionnaires. In Peru, and other similar LMICs, it would not be possible to monitor the burden of high CVD risk with a laboratory-based model and the non-laboratory-based model rises as the only alternative. Nonetheless, evidence regarding the agreement between the WHO laboratory-based and non-laboratory-based models in countries from Latin America is missing.[12] Whether the WHO non-laboratory-based model delivers predictions similar to those of the WHO laboratory-based model remains unknown in Latin America. However, clinicians need this evidence to inform their choice (non-laboratory-based vs laboratory-based model), and to interpret the results of the non-laboratory-based model under the assumption that the laboratory-based model is the gold standard.[12] To provide this evidence for practitioners in Peru, we determined the agreement between the risk predictions computed with the non-laboratory-based model and laboratory-based model in a nationally-representative sample of Peruvian adults.

For peer review only

METHODS

Data sources

This is a cross-sectional study of a national survey conducted by the National Centre for Food and Nutrition (CENAN, for its acronym in Spanish) of Peru. CENAN's survey was conducted between 2017-2018 on a nationally-representative sample of Peruvian adults aged between 18-59 years.[15] Of note, this is the most recent nationally-representative survey conducted in Peru that included blood biomarkers (e.g., lipid profile). CENAN's survey adhered to ethical guidelines and followed a standardised protocol that has been published elsewhere.[15] Each participant

was informed about all procedures and techniques used in the survey; also, participants could have left the study at any time and their personal information was kept confidential.[15]

The CENAN's survey sample was computed using the formula shown in Supplementary Figure 1 and followed a probabilistic sampling design approach with two stages.[15] First, clusters were randomly selected considering three strata: 1) Urban areas except Lima city, 2) Rural areas, and 3) Lima city. Then, households (of adults aged 18-59 years living in) were randomly selected within each cluster. To be selected for the survey sample, participants had to fulfil the following inclusion criteria: 1) Adults aged 18-59 years, and 2) Fasting 9-12 hours for blood biomarkers. The following participants were excluded: 1) Pregnant and postpartum women, 2) Adults taking medication that could alter glucose and lipid profiles, 3) Adults with congenital diseases that could limit anthropometrics measurement (e.g., Down syndrome).

Study population

We analysed a complete-case sample regarding all the laboratory- and office-based 2019 ten-year WHO CVD risk score variables (see Variables section). We studied men and women aged between 30-59 years. The younger age limit was decided because CVD risk models are not recommended in younger individuals; the older age limit was decided because of the survey design. A flowchart of data cleaning is shown in Supplementary figure 2. We did not apply other selection criteria.

Although the 2019 ten-year WHO CVD risk models were developed for people aged 40-80 years,[12] for people aged <40 years we assumed they had 40 years for the absolute CVD risk computation. This is consistent with the Package of essential noncommunicable (PEN) disease interventions for primary health care in low-resource settings,[16] and was also done in a recent global work.[17]

Variables

We calculated the ten-year CVD risk at the individual-level following the laboratory- and non-laboratory-based 2019 ten-year WHO CVD risk model.[12] We used the *whocvdrisk* command in

For peer review only - http://bmjopen.bmj.com/site/about/guidelines.xhtml

STATA, which was developed by the authors of the 2019 ten-year WHO CVD risk charts.[18] For the laboratory-based model, scores were calculated based on: age (years), current smoking status (yes/no), systolic blood pressure (SBP, mmHg), history of self-reported diabetes diagnosis (yes/no), and total cholesterol (mmol/L).[12] For the non-laboratory-based model, we used age (years), current smoking status (yes/no), systolic BP (mmHg) and body mass index (BMI, kg/m^2).[12]

The CENAN's survey collected anthropometrics and three BP measurements which were taken by trained fieldworkers following a standard protocol.[15] We computed BMI using measured weight (kg) divided by the square of height (meters); for descriptive purposes, we classified BMI in three levels: normal weight (BMI <25 kg/m^2), overweight (BMI ≥25-29.9 kg/m^2), and obesity (BMI ≥30 kg/m^2). BMI records outside the range 10-80 kg/m^2 were discarded. As the third BP measurement was only available in few participants (<2% of the initial sample), we used the second SBP measurement only (i.e., the first and third SBP records were discarded in the main analysis). Of note, there were no substantial differences between the first and second SBP records (Supplementary table 1); nonetheless, we performed a sensitivity analysis using the mean SBP of first and second SBP records. We discarded any SBP records outside the range 70-270 mmHg.

For people who self-reported being under antihypertensive treatment, we used the pre-treatment SBP; this is consistent with the PEN protocol and with a previous global work.[16,17]. Pre-treatment SBP was computed as: pre-treatment systolic blood pressure = (current systolic blood pressure-6.3)/0.9.[19] Conversely, for those not taking antihypertensive treatment, we used the recorded SBP as was.

We defined current smoker with one question coded as no versus yes: *do you currently smoke any tobacco product such as cigarettes, cigars or pipes?* Self-reported information about a prior history of diabetes was assessed by a question also coded as no versus yes: *have you ever been told by a physician or another healthcare worker that you have high blood sugar or diabetes?*

For peer review only

Total cholesterol was obtained via enzymatic colorimetric method.[15] Because CENAN's survey data had total cholesterol in mg/dl, these values were divided by 38.67 to obtain total cholesterol in mmol/L.

Statistical analysis

We determined the agreement between the absolute CVD risk predicted with the WHO laboratory- and non-laboratory-based models following three methods: Bland Altman plots, Lin's concordance coefficient correlation (LCCC), and kappa statistic. We considered the absolute CVD risk as a continuous variable, and the agreement between both models was examined using Bland Altman plots and the LCCC. Furthermore, we considered the CVD risk as a categorical variable, and divided into three groups: <5%, 5-9%, 10-19%; because there were no observations in the high-risk category (CVD risk ≥20%), agreement was not examined in this group. For these categories, we evaluated the agreement using the kappa statistic. For the Bland Altman plots, LCCC, and the kappa statistic, results were stratified by sex, 10-year age groups, BMI categories, smoking status, self-reported diabetes diagnosis and urban/rural location.

In the Bland Altman plots, the risk difference between the laboratory- and non-laboratory-based absolute cardiovascular predicted risk was plotted on the vertical axis, and the mean of both scores on the horizontal axis.[20] As the true risk of CVDs at the individual-level is uncertain, the mean of both scores is the best available estimate.[20,21] The 95% of the limit of agreement was represented by the mean difference of both scores ± two standard deviations; this limit provides an interval in which 95% of the differences between both scores would be expected to lie.[20] The LCCC between the laboratory- and non-laboratory-based absolute cardiovascular predicted risks was also evaluated. The agreement based on the LCCC ranges between -1 and 1, with 1 suggesting a perfect agreement. The categorical agreement was evaluated using the Kappa statistic. Kappa <0 indicated less than chance agreement, and values 0.01–0.20, 0.21–0.40, 0.41–0.60, 0.61–0.80, and 0.81–0.99 represented slight, fair, moderate, substantial, and almost perfect agreement, respectively.[22]

For peer review only

All analyses code were conducted with R (version 4.0.3) and STATA (version 17.0, College Station, Texas 77845, USA). Population characteristics along with their 95% confidence interval (95% CI) were summarized accounting for the complex survey design of the CENAN's survey.[15]

Patient and public involvement

No patient involved.

RESULTS

Our pooled dataset included 663 participants (Supplementary figure 2). The mean age was 44.0 years (95% CI: 43.2; 44.7) and the proportion of men was 41.5%. The mean SBP was 108.9 mmHg (95% CI: 107.5; 110.3 mmHg) and the mean BMI was 28.8 kg/m^2 (95% CI: 28.3; 29.2 kg/m^2). The proportion of people with overweight was 41.0% (95% CI: 36.6; 45.6 %), whereas 35.9% (95% CI: (31.6; 40.5%) of the population were obese. The mean total cholesterol was 4.9 mmol/L (95% CI: 4.8; 5.0 mmol/L), 11.7% (95% CI: 8.8; 15.3%) of the population were smokers and 7.1% (95% CI: 5.0%; 9.8%) had diabetes (Table 1).

Table 1. Weighted distribution of the predictors in the 2019 WHO CVD risk models, overall and by sex.

	Total	Men	Women
**Sample size**	663	280	383
**Age (mean and 95% CI, years)**	44 (43.2-44.7)	44.2 (43-45.4)	43.8 (42.9-44.7)
**Proportion of people aged 30-39 years (95% CI, %)**	35.8 (31.6-40.3)	37.2 (31-43.9)	34.8 (29.4-40.7)
**Proportion of people aged 40-49 years (95% CI, %)**	34 (29.8-38.4)	29.5 (24-35.7)	37.1 (31.5-43.1)
**Proportion of people aged 50-59 years (95% CI, %)**	30.2 (26.3-34.5)	33.3 (27.1-40)	28 (23.5-33.1)
**Systolic blood pressure (mean and 95% CI, mmHg)**	108.9 (107.5-110.3)	116.1 (113.8-118.4)	103.8 (102.3-105.3)
**Diastolic blood pressure (mean and 95% CI, mmHg)**	71.9 (71-72.8)	74.6 (73.1-76.1)	70 (69-71)
**Body mass index (mean and 95% CI, kg/m2)**	28.8 (28.3-29.2)	28.3 (27.6-29)	29.1 (28.5-29.7)
**Proportion of people with normal weight (95% CI, %)**	23.1 (19.5-27.2)	24.5 (19.5-30.4)	22.1 (17.2-27.9)

For peer review only

**Proportion of people with overweight (95% CI, %)**	41 (36.6-45.6)	45.3 (38.9-52)	37.9 (31.9-44.3)
**Proportion of people with obesity (95% CI, %)**	35.9 (31.6-40.5)	30.1 (24.1-36.9)	40 (34-46.2)
**Total cholesterol (mean and 95% CI, mmol/L)**	4.9 (4.8-5)	4.8 (4.6-5)	4.9 (4.8-5.1)
**Proportion of smokers (95% CI, %)**	11.7 (8.8-15.3)	21.1 (15.8-27.6)	5 (3-8.3)
**Proportion of people with diabetes (95% CI, %)**	7.1 (5-9.8)	7.8 (4.7-12.6)	6.5 (4.3-9.9)
**Laboratory-based CVD risk score (mean and 95% CI, %)**	2 (1.8-2.2)	2.6 (2.3-2.9)	1.6 (1.4-1.7)
**Non-laboratory-based CVD risk score (mean and 95% CI, %)**	2 (1.8-2.1)	2.7 (2.4-3)	1.5 (1.4-1.6)

For peer review only

Absolute cardiovascular risk according to the 2019 WHO cardiovascular risk models

Overall, there were no substantial differences between the mean absolute cardiovascular risk computed with the laboratory-based and non-laboratory-based models (Table 1). The mean absolute cardiovascular risk was 2.0% (95% CI: 1.8%; 2.2%) according to the laboratory-based model, and 2.0% (95% CI: 1.8%; 2.1%) according to the non-laboratory-based model. In both models, the mean absolute cardiovascular risk was higher in men than women. The sensitivity analysis (using the mean of two SBP records) yielded the same findings in the overall sample: 2.0% (95% CI: 1.8; 2.2%) in the laboratory-based-model and 2.0 (95% CI: 1.8; 2.1%) in the non-laboratory-based model.

Mean difference between risk predictions

Overall, the mean difference between the laboratory-based and the non-laboratory-based models was 0.03% (95% CI: -0.03%; 0.10%). According to sex, the mean difference between models was -0.02% (95% CI: -0.14%; 0.09%) in men, and 0.08% (95% CI: 0.01%; 0.15%) in women. According to age, the mean difference between models was -0.07% (95% CI: -0.12%; 0.04%) in people aged 30-39 years, 0.04% (95% CI: -0.08%; 0.15%) in people aged 40-49 years, and 0.17% (95% CI: 0.02%; 0.32%) in people aged 50-59 years. Stratified by BMI categories, the mean difference was 0.10% (95% CI: 0.02%; 0.18%) in people with normal weight, 0.08% (95% CI: 0.00%; 0.17%) in people with overweight, and -0.07% (95% CI: -0.21%; 0.07%) in people with obesity. The mean difference was 0.05% (95% CI: -0.34%; 0.44%) in smokers and 0.03% (95% CI: -0.02%; 0.09%) in non-smokers. According to self-reported diabetes status, mean difference was 2.08% (95% CI: 1.73%; 2.44%) in people with self-reported diabetes, and -0.11% (95% CI: -0.15%; 0.07%) in people without self-reported diabetes. The sensitivity analysis provided similar results across all variables; for example, the largest mean difference was also observed in people with self-reported diabetes (2.09% (95% CI: 1.73; 2.45%).

Bland-Altman plots and limits of agreement.

The limit of agreement was slightly narrower for women (-1.23; 1.39) compared to men (-1.93; 1.89) (Figure 1). The limit of agreement widened with older ages and higher BMI levels; for

For peer review only - http://bmjopen.bmj.com/site/about/guidelines.xhtml

example, the limit of agreement was narrower in people aged 30-39 years (-0.72; 0.56) compared to those aged 50-59 years (-1.96; 2.29) (Figure 2), and for people with normal weight (-0.90; 1.09) compared to those with obesity (-2.15; 2.02) (Figure 3). According to smoking and self-reported diabetes status, the limit of agreement was wider in those who had the condition. For example, the limit of agreement in smokers (-3.13; 3.24) was wider compared to non-smokers (-1.25; 1.32) (Figure 4). Similarly, in people with self-reported diabetes (-0.21; 4.37) the limit of agreement was wider compared to people without self-reported diabetes (-1.17; 0.95) (Figure 5). Notably, the sensitivity analysis resulted in similar limits of agreement in all variables (Supplementary figures 3-7).

Agreement by Lin's concordance coefficient correlation

The overall agreement between scores as per the LCCC was 0.87 (95% CI: 0.85; 0.89), and it was virtually the same in men (0.87 (95% CI 0.84; 0.89)) and women (0.85 (95% CI 0.82; 0.87)). Across age groups, the highest agreement was seen in the 30-39 age group (0.87 (95% CI 0.84; 0.9)). Across BMI categories, it was the normal BMI category which had the highest agreement (0.90 (95% CI 0.87; 0.92)). Overall, the lowest agreement values were seen across smokers (0.81 (95% CI 0.72; 0.88)), those aged 40-49 years (0.74 (95% CI 0.67; 0.79)), and those with self-reported diabetes (0.74 (95% CI 0.63; 0.82)) (Table 2). Similar results were seen in the sensitivity analysis (Supplementary table 2).

Table 2. Lin's concordance coefficient correlation showing agreement between laboratory and non-laboratory-based risk models according to the predictors in the 2019 WHO CVD risk models and urban/rural location

Variables	Categories	Lin's concordance coefficient correlation (95% CI)
**Sex**	**Men**	0.87 (0.84 - 0.89)
	**Women**	0.85 (0.82 - 0.87)
**Age (years)**	**30-39**	0.87 (0.84 - 0.9)
	**40-49**	0.74 (0.67 - 0.79)
	**50-59**	0.83 (0.78 - 0.86)
**Body mass index category**	**Normal**	0.9 (0.87 - 0.92)

For peer review only - http://bmjopen.bmj.com/site/about/guidelines.xhtml

	**Overweight**	0.87 (0.85 - 0.9)
	**Obese**	0.86 (0.82 - 0.89)
**Smoking status**	**Smoker**	0.81 (0.72 - 0.88)
	**Non-smoker**	0.86 (0.84 - 0.88)
**Diabetes status**	**With self−reported diabetes**	0.74 (0.63 - 0.82)
	**Not with self−reported diabetes**	0.91 (0.9 - 0.92)
**Urban or rural**	**Urban**	0.88 (0.85 - 0.9)
	**Rural**	0.85 (0.82 - 0.88)

Categorical agreement

In the overall population, there was a slightly larger number of people categorized as having an absolute CVD risk of 5-9% and 10-19% with the laboratory-based-model compared with the non-laboratory-based model (Supplementary table 3). For example, the laboratory-based model categorized 37 people in the 5-9% CVD risk category, whereas the non-laboratory-based model categorized 26 people. Overall, the agreement between risk categories was substantial (kappa=0.62), and it was better for men (kappa=0.70) compared to women (kappa=0.44) (Supplementary table 4). Of note, the lowest agreement between risk categories was observed among people with self-reported diabetes (kappa=0.36): out of 14 people with self-reported diabetes in the 5-9% CVD risk category following the laboratory-based-model, 4 were placed in the same category following the non-laboratory-based model, as the rest were placed in the 0-5% CVD risk category. The categorical agreement according to all variables is presented in Supplementary tables 3-9.

DISCUSSION

Main findings

In this work, we evaluated the agreement between the CVD risk estimates predicted with the 2019 WHO ten-year laboratory-based and non-laboratory-based models in a nationally-representative sample of Peruvian adults. The mean absolute predicted CVD risk according to both models in the general population was virtually the same. In addition, we found that the limits of agreement between both models increased with a higher CVD risk; for instance, the limits of agreement were

For peer review only - http://bmjopen.bmj.com/site/about/guidelines.xhtml

wider in smokers and people with self-reported diabetes. We observed good agreement between the laboratory-based and non-laboratory-based models in terms of clinical significance.[21,23] These findings suggest that, in a population with a similar profile to that in this study, practitioners could use either the laboratory-based or non-laboratory-based models. Although the difference is very small, careful interpretation could be needed for people with cardiovascular risk factors: obesity, smokers and people with self-reported diabetes, amongst whom the difference was slightly larger than in their peers without these risk factors.

Public health implications

We provided insights about the applicability of the 2019 WHO non-laboratory-based model in the Peruvian population. This evidence is relevant in terms of clinical practice and public health in Peru and other similar countries (e.g., Andean Latin America), because it informs whether the predictions based on the laboratory-based and non-laboratory-based CVD risk models are equivalent. If so, either of these models could be used without substantial bias, hence supporting the use of the non-laboratory-based model when blood biomarkers are not available. The latter is of special importance in LMICs like Peru, where laboratory measurements are not always available in primary health centres, which represent >98% of all health care facilities in Peru.[24]

According to the three statistical analyses we implemented (Bland-Altman plots, Lin's concordance coefficient correlations; and kappa statistics), our results suggest that the agreement between the laboratory-based and non-laboratory-based models was appropriate among Peruvians with low CVD risk and younger than 60 years. In other words, our results suggest that the laboratory-based and non-laboratory-based models provide similar predictions and may therefore be used interchangeably as needed, though the profile of our study population ought to be considered when extracting or implementing our findings into clinical practice and public health. Of note, the small number of participants in some variables of interest (e.g., only 44 participants had self-reported diabetes) could have explained the broader limits of agreement in our results. Further studies should include a larger number of participants to further confirm whether limits of agreement are wider according to smoking and diabetes status.

For peer review only - http://bmjopen.bmj.com/site/about/guidelines.xhtml

Research in context

The study most comparable to ours evaluated the agreement between the Framingham 10-year CVD risk laboratory and non-laboratory models on a population aged 40-75 years in southern Iran.[23] They found the mean CVD risk following the non-laboratory-based model (9.4%) was higher than the laboratory-based model (6.7%).[23] Additionally, their limits of agreement between both Framingham models in people <60 years old were wider compared to ours in both men (-1.9-1.9 by our estimates versus -2.5%-8.9% by Rezaei et al.[23]) and women (-1.2-1.4 by our estimates versus -2.3%-4.6% by Rezaei et al.[23]). This could be explained by the fact that Rezaei et al.[23] included an older population, which tend to have higher levels of CVD risk factors and therefore a higher absolute CVD risk. As limits of agreement between two models tend to widen with higher CVD risk,[21,23] our limits of agreement would presumably be wider if we had studied a similar population to that of the work by Rezaei et al.[23] The differences between our results could be further explained by the CVD risk score herein used. We used the 2019 WHO CVD risk models,[12] whereas Rezaei et al. used the Framingham risk scores. The Framingham risk score was developed for a more specific population (Caucasians in the US),[25] yet the 2019 WHO CVD risk model was developed and recalibrated for a global use (e.g., those living in LMICs).[12]

The agreement between the 2019 WHO laboratory- and non-laboratory-based model was also explored in the global work convened by the WHO.[12] They applied the two models to WHO STEPS surveys, and compared the proportion of people categorized at different levels of predicted CVD risk.[12] Overall, they found moderate agreement between both models, and their discrepancy was attributed to poor performance of the non-laboratory-based model in people with diabetes.[12] This finding is consistent with our results because we found the widest limits of agreement, the lowest LCCC, and the lowest categorical agreement in people with self-reported diabetes. When possible, it would seem reasonable to use the laboratory-based model in those whose have diabetes.

For peer review only - http://bmjopen.bmj.com/site/about/guidelines.xhtml

Potential explanations

In our study, diabetes status was only included in the laboratory-based model (and not in the non-laboratory-based model) and reasonably, we observed the lowest agreement between both models in those with diabetes. That is, in people with diabetes, the non-laboratory-based model underestimated the absolute CVD risk computed following the laboratory-based model. Probably because people with diabetes are already at high CVD risk and the non-laboratory model, we used without diabetes information would underestimate the absolute risk.

Strengths and limitations

To the best of our knowledge, we provided the first evidence of the agreement between the 2019 WHO CVD risk laboratory-based and non-laboratory-based models; furthermore, we leveraged on a nationally-representative survey conducted in a LMIC.[26] Different CVD risk equations have been created but none has proved to produce reliable estimates for LMICs. The 2019 WHO CVD risk charts were adapted for LMICs using extensive datasets for its derivation, recalibration, and validation, which brings them several advantages over previous risk charts. Nonetheless, this study has also limitations. First, our study population was young (30-59 years), mostly women (58%), and with overall low absolute cardiovascular predicted risk. This led to the observation that no one in the study population had an absolute cardiovascular risk ≥20%. Thus, we could only draw conclusions for people within the low and medium CVD risk range, and with a similar demographic and risk factor profile. We acknowledge that further subgroup analysis could be relevant, for example by diabetes status. However, because of data availability and the reduced number of observations in some groups, this subgroup analyses would be impossible to conduct. Future work in Peru and Latin America should verify our results with a larger, older and more diverse population. Second, as CENAN's survey did not include history of cardiovascular events, we assumed all participants were free of CVD to use the 2019 WHO CVD risk score. This approach could have led to higher absolute cardiovascular risk because people who have had a cardiovascular event (e.g., myocardial infarction) are at higher risk of another cardiovascular event. Nonetheless, considering that our study population was young and therefore with a low incidence of cardiovascular diseases,[3] the proportion of people with history of CVD excluded from the total sample size would have been small; not excluding potentially a small group may

For peer review only - http://bmjopen.bmj.com/site/about/guidelines.xhtml

not have altered the overall results. Third, we only used one blood pressure record (the second SBP out of two measurements). Ideally, and following standard protocols recommended by WHO and other international organizations,[27,28] we should have used the average of multiple records having discarded the very first measurement. This was not possible with the available data because they only measured blood pressure twice. Nonetheless, we performed a sensitivity analysis using the mean SBP of the first and second records and had virtually the same results as in our main analysis in which only the second SBP record was used.

Conclusions

The absolute cardiovascular predicted risk was similar between the laboratory-based and non-laboratory based 2019 WHO cardiovascular risk models. Pending validation from longitudinal studies, the non-laboratory-based model (instead of the laboratory-based which requires additional resources) could be used in Peruvian population. Nonetheless, it should be noted that the agreement between these models was less clear in people with cardiovascular risk factors: obesity, smokers and people with diabetes. While universal health coverage momentum helps to have laboratory tests in (most) primary care facilities to use the laboratory-based model, it seems reasonable to use the non-laboratory-based model for primary prevention of CVD following the risk stratification approach.

For peer review only

For peer review only - http://bmjopen.bmj.com/site/about/guidelines.xhtml

FIGURES

Figure 1. Bland Altman plots showing agreement between laboratory and non-laboratory-based risk scores according to sex.

Figure 2. Bland Altman plots showing agreement between laboratory and non-laboratory-based risk scores according to age groups.

Figure 3. Bland Altman plots showing agreement between laboratory and non-laboratory-based risk scores according to body mass index categories

Figure 4. Bland Altman plots showing agreement between laboratory and non-laboratory-based risk scores according to smoking status.

Figure 5. Bland Altman plots showing agreement between laboratory and non-laboratory-based risk scores according to self-reported diabetes status.

For peer review only - http://bmjopen.bmj.com/site/about/guidelines.xhtml

Contributorship: RMC-L conceived the idea with WCG-V. WCG-V conducted the analysis with support from GAQ-V and FFV-R. WCG-V wrote the first draft of the manuscript with support from GAQ-V, FFV-R, AB-O and RMC-L. All authors provided relevant scientific contribution. All authors approved the submitted version.

Funding statement: RMC-L is supported by a Wellcome Trust International Training Fellowship (214185/Z/18/Z). The funder had no role in the study design, analysis, interpretation or decision to publish. The authors are collectively responsible for the accuracy of the data. The arguments and opinions in this work are those of the authors alone, and do not represent the position of the institutions to which they belong.

Competing of interests: None to declare.

Ethics: This study used de-identified nationally-representative survey data that can be requested from the CENAN and is used for independent analyses.[26] Authors had no access to the participants' personal information. Therefore, this work was deemed as of minimal risk and we did not seek approval by an Ethics Committee to conduct the analysis.

Data sharing: Datasets available upon request from the CENAN.

Word count: 4,220

For peer review only

REFERENCES

1. World Health Organization. Cardiovascular diseases (CVDs) [Internet]. [cited 2021 Aug 15]. Available from: https://www.who.int/news-room/fact-sheets/detail/cardiovascular-diseases-(cvds)

2. GBD 2019 Diseases and Injuries Collaborators. Global burden of 369 diseases and injuries in 204 countries and territories, 1990-2019: a systematic analysis for the Global Burden of Disease Study 2019. Lancet. 2020 Oct 17;396(10258):1204–22.

3. GBD Results Tool | GHDx [Internet]. Available from: http://ghdx.healthdata.org/gbd-results-tool

4. Prabhakaran D, Anand S, Watkins D, Gaziano T, Wu Y, Mbanya JC, et al. Cardiovascular, respiratory, and related disorders: key messages from Disease Control Priorities, 3rd edition. Lancet. 2018 Mar 24;391(10126):1224–36.

5. Arnett DK, Blumenthal RS, Albert MA, Buroker AB, Goldberger ZD, Hahn EJ, et al. 2019 ACC/AHA Guideline on the Primary Prevention of Cardiovascular Disease: Executive Summary: A Report of the American College of Cardiology/American Heart Association Task Force on Clinical Practice Guidelines. Circulation. 2019 Sep 10;140(11):e563–95.

6. PAHO/WHO | Pan American Health Organization. HEARTS in the Americas - [Internet]. [cited 2021 Aug 16]. Available from: https://www.paho.org/en/hearts-americas

7. Whelton PK, Carey RM, Aronow WS, Casey DE, Collins KJ, Dennison Himmelfarb C, et al. 2017 ACC/AHA/AAPA/ABC/ACPM/AGS/APhA/ASH/ASPC/NMA/PCNA Guideline for the Prevention, Detection, Evaluation, and Management of High Blood Pressure in Adults: Executive Summary: A Report of the American College of Cardiology/American Heart Association Task Force on Clinical Practice Guidelines. Hypertension. 2018 Jun;71(6):1269–324.

8. Williams B, Mancia G, Spiering W, Agabiti Rosei E, Azizi M, Burnier M, et al. 2018 ESC/ESH Guidelines for the management of arterial hypertension: The Task Force for the management of arterial hypertension of the European Society of Cardiology and the European Society of Hypertension: The Task Force for the management of arterial hypertension of the European Society of Cardiology and the European Society of Hypertension. J Hypertens. 2018 Oct;36(10):1953–2041.

9. NICE National Institute for Health and Care Excellence. Hypertension in adults: diagnosis and management | Guidance [Internet]. NICE; 2019 [cited 2021 Aug 16]. Available from: https://www.nice.org.uk/guidance/ng136

10. Karmali KN, Persell SD, Perel P, Lloyd-Jones DM, Berendsen MA, Huffman MD. Risk scoring for the primary prevention of cardiovascular disease. Cochrane Database Syst Rev. 2017 Mar 14;3:CD006887.

11. Damen JAAG, Hooft L, Schuit E, Debray TPA, Collins GS, Tzoulaki I, et al. Prediction models for cardiovascular disease risk in the general population: systematic review. BMJ. 2016 May 16;353:i2416.

12. WHO CVD Risk Chart Working Group. World Health Organization cardiovascular disease risk charts: revised models to estimate risk in 21 global regions. Lancet Glob Health. 2019 Oct;7(10):e1332–45.

13. Wilson ML, Fleming KA, Kuti MA, Looi LM, Lago N, Ru K. Access to pathology and laboratory medicine services: a crucial gap. Lancet. 2018 May 12;391(10133):1927–38.

14. Marcus ME, Manne-Goehler J, Theilmann M, Farzadfar F, Moghaddam SS, Keykhaei M, et al. Use of statins for the prevention of cardiovascular disease in 41 low-income and

For peer review only

middle-income countries: a cross-sectional study of nationally representative, individual-level data. Lancet Glob Health. 2022 Mar;10(3):e369–79.

15. Centro Nacional de Alimentación y Nutrición. ESTADO NUTRICIONAL EN ADULTOS DE 18 A 59 AÑOS, PERÚ: 2017 - 2018 [Internet]. 2021. Available from: https://web.ins.gob.pe/sites/default/files/Archivos/cenan/van/sala_nutricional/sala_3/2021/Informe%20Tecnico-%20Estado%20nutricional%20en%20adultos%20de%2018%20a%2059%20a%C3%B1os%2CVIANEV%202017-2018.pdf

16. Package of essential noncommunicable (PEN) disease interventions for primary health care in low-resource setting. WHO PEN Protocol 1 Prevention of HEart Attacks, Stroke and Kidney Disease through Integrated Management of Diabetes and Hypertension [Internet]. Available from: https://www.who.int/ncds/management/Protocol1_HeartAttack_strokes_kidneyDisease.pdf?ua=1

17. Peiris D, Ghosh A, Manne-Goehler J, Jaacks LM, Theilmann M, Marcus ME, et al. Cardiovascular disease risk profile and management practices in 45 low-income and middle-income countries: A cross-sectional study of nationally representative individual-level survey data. PLoS Med. 2021 Mar;18(3):e1003485.

18. University of Cambridge. Cardiovascular Epidemiology Unit. Programs [Internet]. Cardiovascular Epidemiology Unit. [cited 2021 Aug 15]. Available from: https://www.phpc.cam.ac.uk/ceu/population-resources-and-tools/programs/

19. Law MR, Morris JK, Wald NJ. Use of blood pressure lowering drugs in the prevention of cardiovascular disease: meta-analysis of 147 randomised trials in the context of expectations from prospective epidemiological studies. BMJ. 2009 May 19;338:b1665.

20. Bland JM, Altman DG. Statistical methods for assessing agreement between two methods of clinical measurement. Lancet. 1986 Feb 8;1(8476):307–10.

21. Jones CA, Ross L, Surani N, Dharamshi N, Karmali K. Framingham ten-year general cardiovascular disease risk: agreement between BMI-based and cholesterol-based estimates in a South Asian convenience sample. PLoS One. 2015;10(3):e0119183.

22. Viera AJ, Garrett JM. Understanding interobserver agreement: the kappa statistic. Fam Med. 2005 May;37(5):360–3.

23. Rezaei F, Seif M, Gandomkar A, Fattahi MR, Hasanzadeh J. Agreement between laboratory-based and non-laboratory-based Framingham risk score in Southern Iran. Sci Rep. 2021 May 24;11(1):10767.

24. Gobierno del Perú. Conocer establecimientos de salud del Primer Nivel de Atención [Internet]. 2022 [cited 2022 May 17]. Available from: https://www.gob.pe/16727

25. D'Agostino RB, Vasan RS, Pencina MJ, Wolf PA, Cobain M, Massaro JM, et al. General cardiovascular risk profile for use in primary care: the Framingham Heart Study. Circulation. 2008 Feb 12;117(6):743–53.

26. Guzman-Vilca WC, Yovera-Juarez EA, Tarazona-Meza C, García-Larsen V, Carrillo-Larco RM. Sugar-Sweetened Beverage Consumption in Adults: Evidence from a National Health Survey in Peru. Nutrients. 2022 Jan 28;14(3):582.

27. World Health Organization. STEPwise Approach to NCD Risk Factor Surveillance (STEPS) [Internet]. [cited 2022 Jan 10]. Available from: https://www.who.int/teams/noncommunicable-diseases/surveillance/systems-tools/steps

For peer review only - http://bmjopen.bmj.com/site/about/guidelines.xhtml

28. Poulter NR, Borghi C, Damasceno A, Jafar TH, Khan N, Kokubo Y, et al. May Measurement Month 2019: results of blood pressure screening from 47 countries. Eur Heart J Suppl. 2021 May;23(Suppl B):B1–5.

For peer review only

For peer review only - http://bmjopen.bmj.com/site/about/guidelines.xhtml

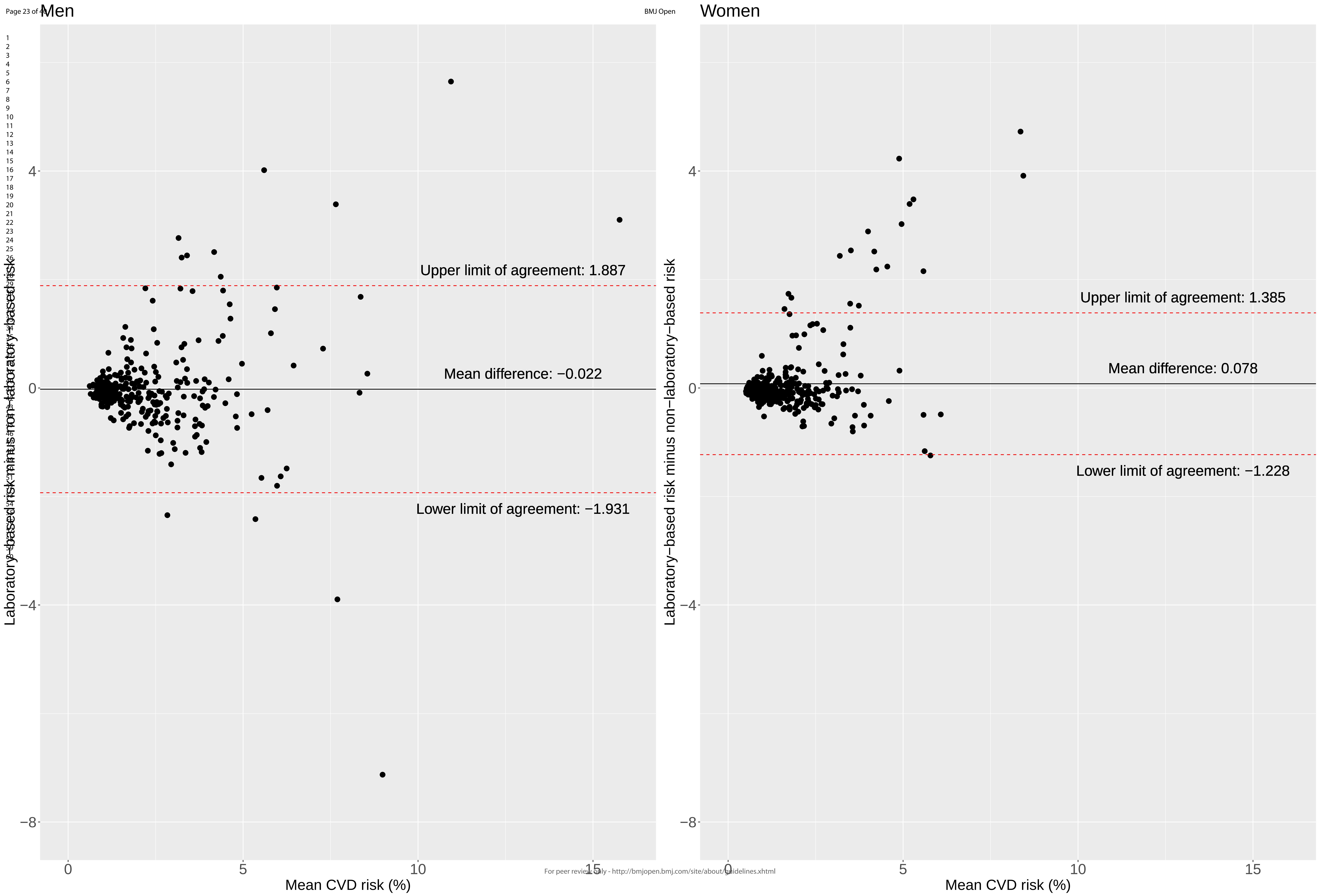
Men
Upper limit of agreement: 1.887
Mean difference: −0.022
Lower limit of agreement: −1.931
Laboratory−based risk minus non−laboratory−based risk
Mean CVD risk (%)
Women
Upper limit of agreement: 1.385
Mean difference: 0.078
Lower limit of agreement: −1.228
Laboratory−based risk minus non−laboratory−based risk
Mean CVD risk (%)

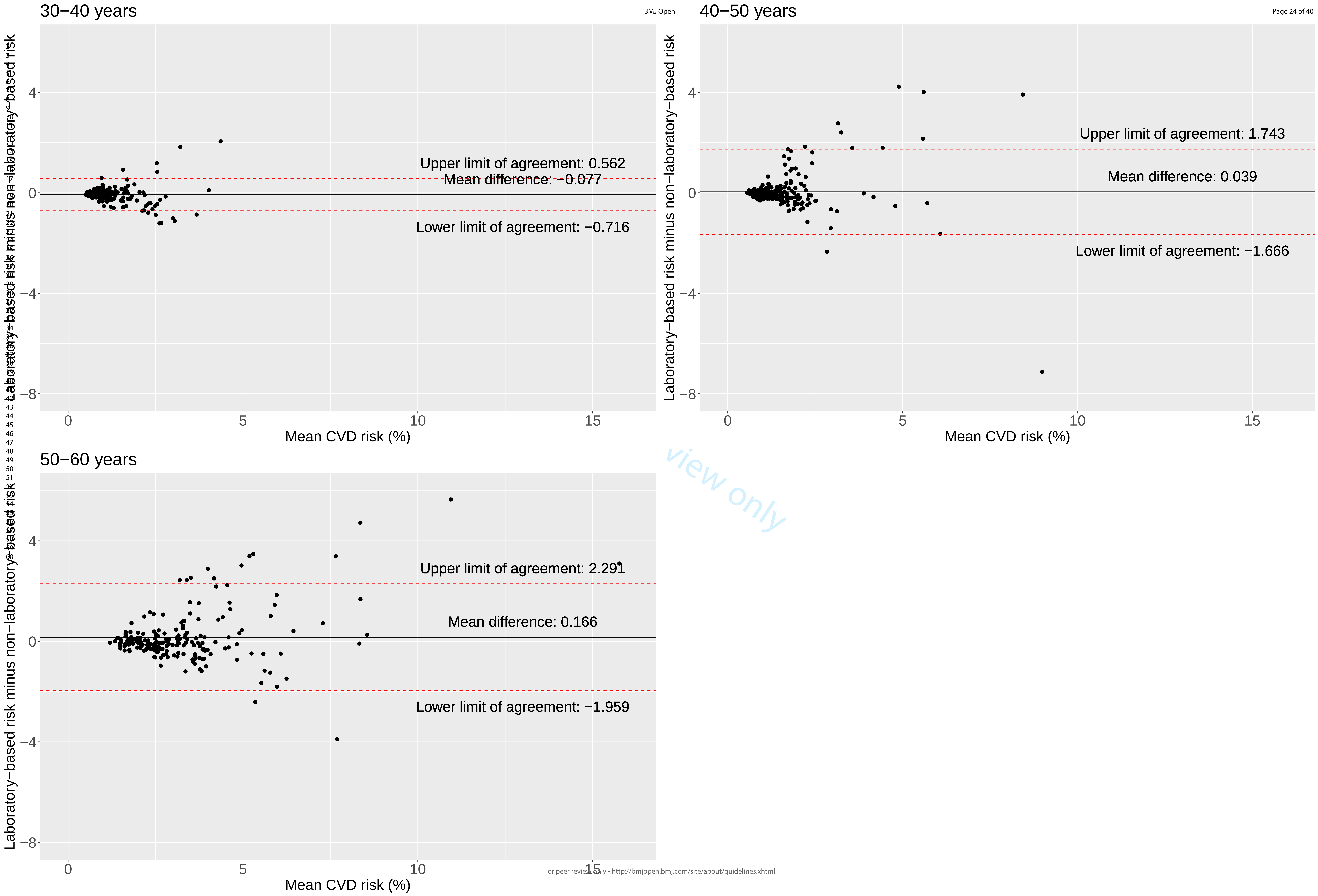

For peer review only - http://bmjopen.bmj.com/site/about/guidelines.xhtml

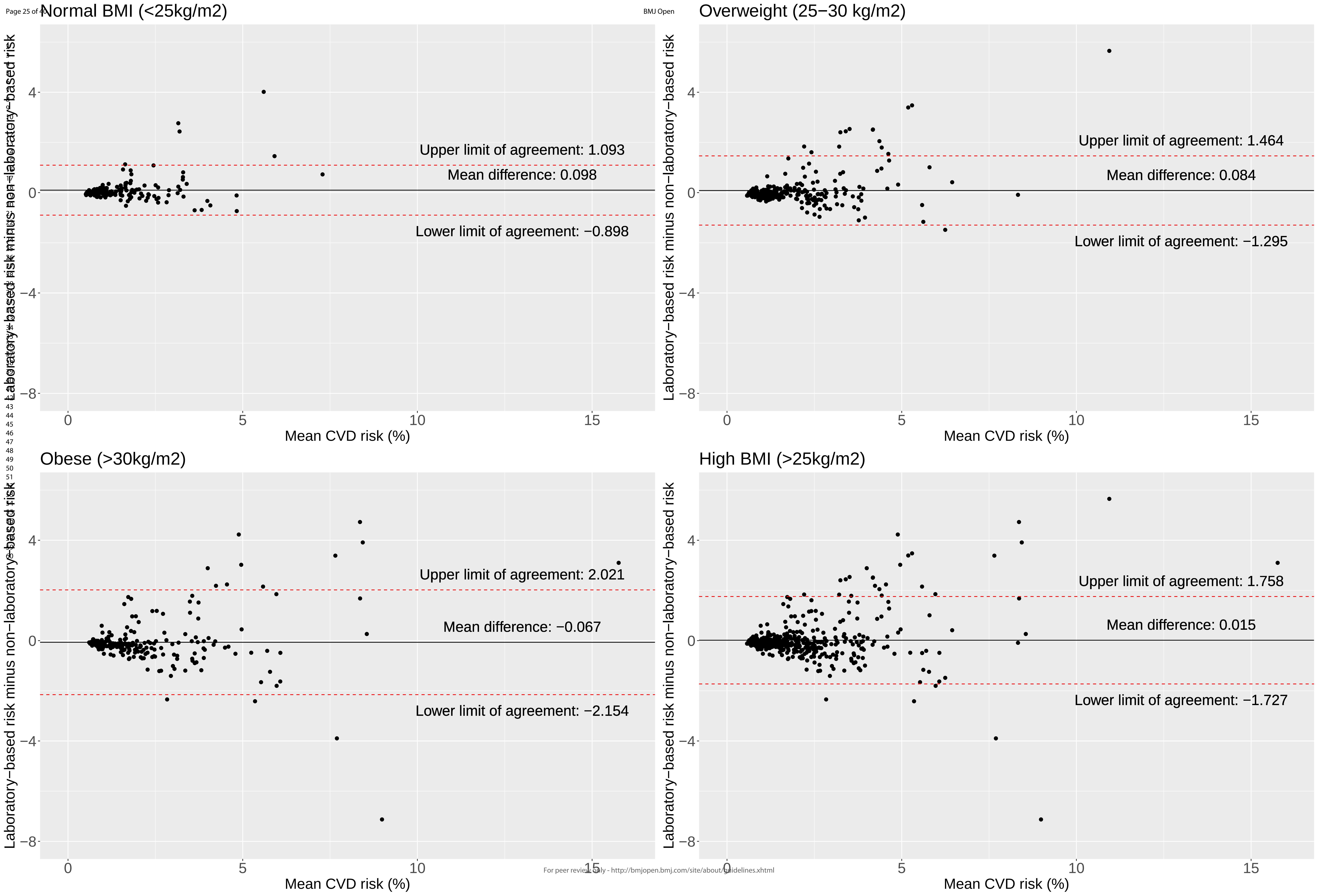
Normal BMI (<25kg/m2)
Laboratory−based risk minus non−laboratory−based risk
Upper limit of agreement: 1.093
Mean difference: 0.098
Lower limit of agreement: −0.898
Mean CVD risk (%)
Overweight (25−30 kg/m2)
Upper limit of agreement: 1.464
Mean difference: 0.084
Lower limit of agreement: −1.295
Obese (>30kg/m2)
Upper limit of agreement: 2.021
Mean difference: −0.067
Lower limit of agreement: −2.154
High BMI (>25kg/m2)
Upper limit of agreement: 1.758
Mean difference: 0.015
Lower limit of agreement: −1.727

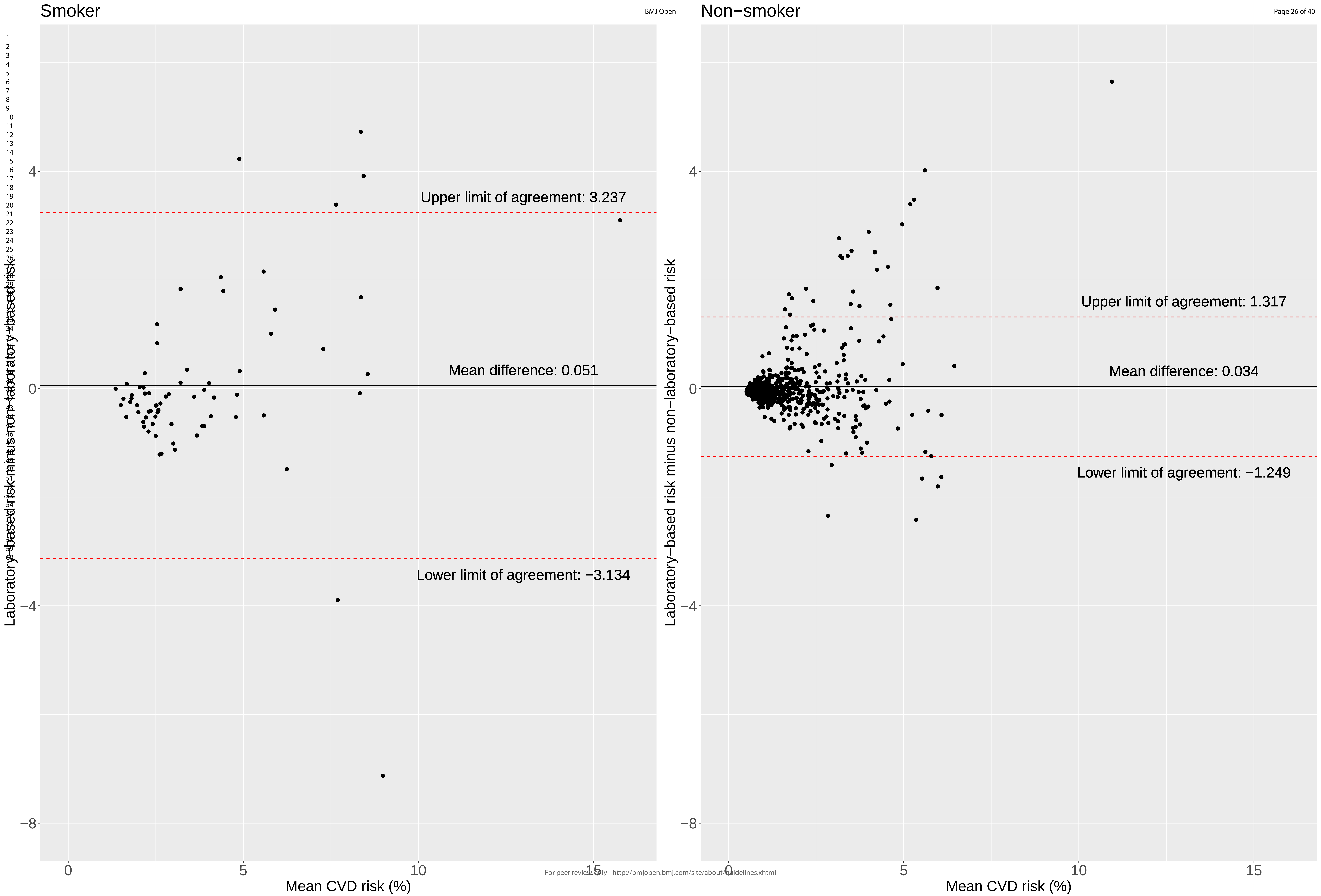
Smoker
Non−smoker
Upper limit of agreement: 3.237
Mean difference: 0.051
Lower limit of agreement: −3.134
Upper limit of agreement: 1.317
Mean difference: 0.034
Lower limit of agreement: −1.249
Laboratory−based risk minus non−laboratory−based risk
Mean CVD risk (%)
4
0
−4
−8
0
5
10
15

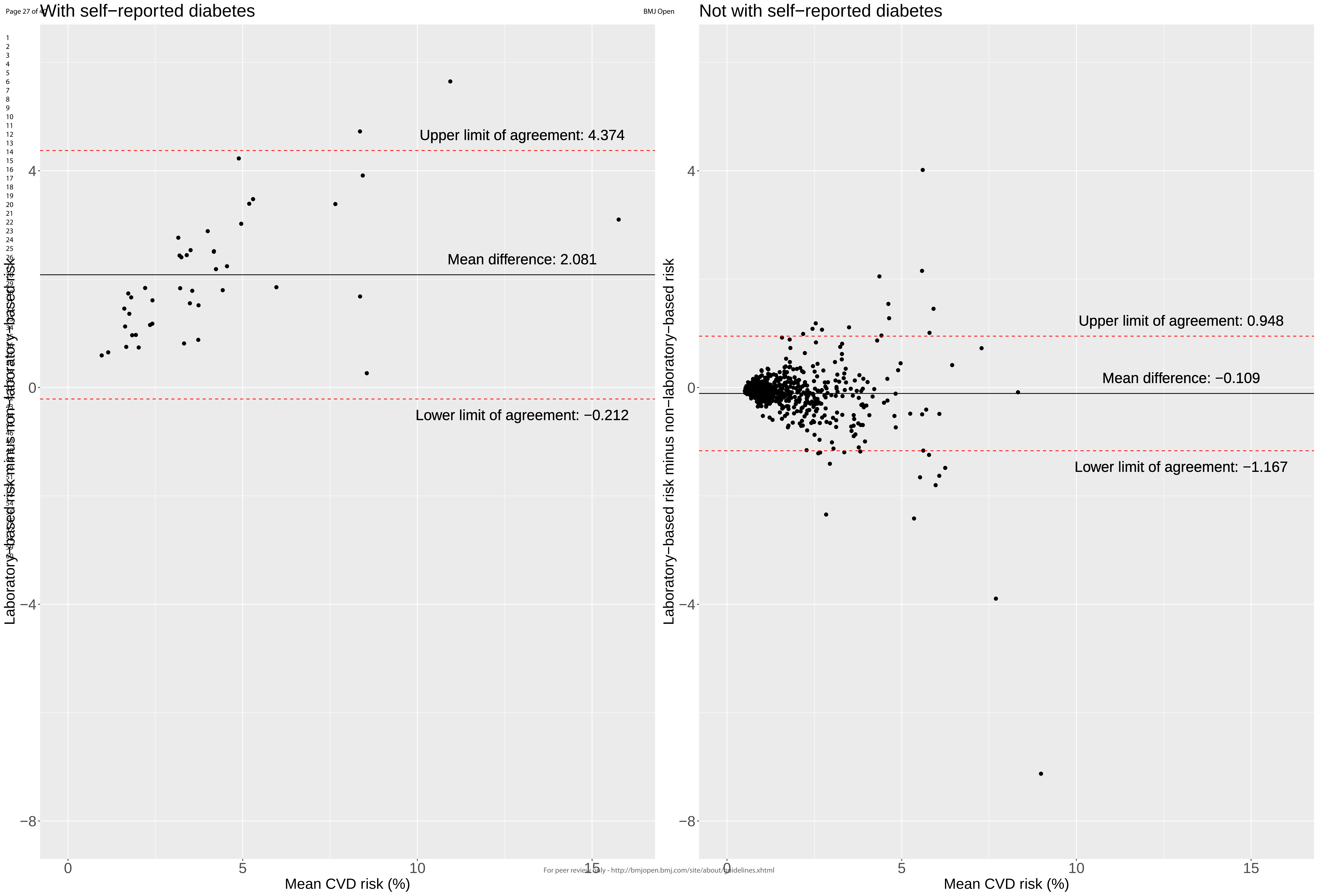
With self−reported diabetes
Not with self−reported diabetes
Upper limit of agreement: 4.374
Mean difference: 2.081
Lower limit of agreement: −0.212
Upper limit of agreement: 0.948
Mean difference: −0.109
Lower limit of agreement: −1.167
Laboratory−based risk minus non−laboratory−based risk
Mean CVD risk (%)
4
0
−4
−8
0
5
10
15

Agreement between the laboratory- and non-laboratory-based 2019 WHO cardiovascular risk charts in Peru

Correspondence author:

Rodrigo M Carrillo-Larco, MD

Department of Epidemiology and Biostatistics

School of Public Health

Imperial College London

rcarrill@ic.ac.uk

For peer review only

For peer review only - http://bmjopen.bmj.com/site/about/guidelines.xhtml

Table of Contents

Table of Contents 2

Supplementary Figure 1. Formula to compute the survey sample size 3

Supplementary Figure 2. Flowchart of data cleaning and inclusion criteria 3

Supplementary Table 1. Summary statistics of the first and second systolic blood pressure (SBP) records in the overall sample 4

Supplementary Table 2. Sensitivity Analysis: Lin's concordance coefficient correlation showing agreement between laboratory and non-laboratory-based risk models according to the predictors in the 2019 WHO CVD risk models and urban/rural location 4

Supplementary Table 3. Kappa statistics showing agreement between laboratory and non-laboratory-based risk scores in the overall sample 5

Supplementary Table 4. Kappa statistics showing agreement between laboratory and non-laboratory-based risk scores by sex 5

Supplementary Table 5. Kappa statistics showing agreement between laboratory and non-laboratory-based risk scores by age groups 5

Supplementary Table 6. Kappa statistics showing agreement between laboratory and non-laboratory-based risk scores by body mass index categories 6

Supplementary Table 7. Kappa statistics showing agreement between laboratory and non-laboratory-based risk scores by smoking status 6

Supplementary Table 8. Kappa statistics showing agreement between laboratory and non-laboratory-based risk scores by diabetes status 6

Supplementary Table 9. Kappa statistics showing agreement between laboratory and non-laboratory-based risk scores by urban/rural location 7

Supplementary Figure 3. Sensitivity Analysis: Bland Altman plots showing agreement between laboratory and non-laboratory-based risk scores according to sex. 7

Supplementary Figure 4. Sensitivity Analysis: Bland Altman plots showing agreement between laboratory and non-laboratory-based risk scores according to age groups. 8

Supplementary Figure 5. Sensitivity Analysis: Bland Altman plots showing agreement between laboratory and non-laboratory-based risk scores according to body mass index categories. 9

Supplementary Figure 6. Sensitivity Analysis: Bland Altman plots showing agreement between laboratory and non-laboratory-based risk scores according to smoking status. 10

Supplementary Figure 7. Sensitivity Analysis: Bland Altman plots showing agreement between laboratory and non-laboratory-based risk scores according to self-reported diabetes status. 11

For peer review only - http://bmjopen.bmj.com/site/about/guidelines.xhtml

Supplementary Figure 1. Formula to compute the survey sample size

$$n_h = \frac{N_h Z^2 P_h Q_h}{(N_h - 1)d^2 + Z^2 P_h Q_h} * TNR$$

Where:
N_h: number of people within an age group in the "h" conglomerate
N_h: number of people in the sample within an age group in the "h" conglomerate
d: error margin assumed in the estimation of P_h
Z: 95% confidence level
TNR (*Tasa de No Respuesta* in Spanish): Expected refusal rate
P_h: prevalence of overweight in adults in the "h" conglomerate

Supplementary Figure 2. Flowchart of data cleaning and inclusion criteria

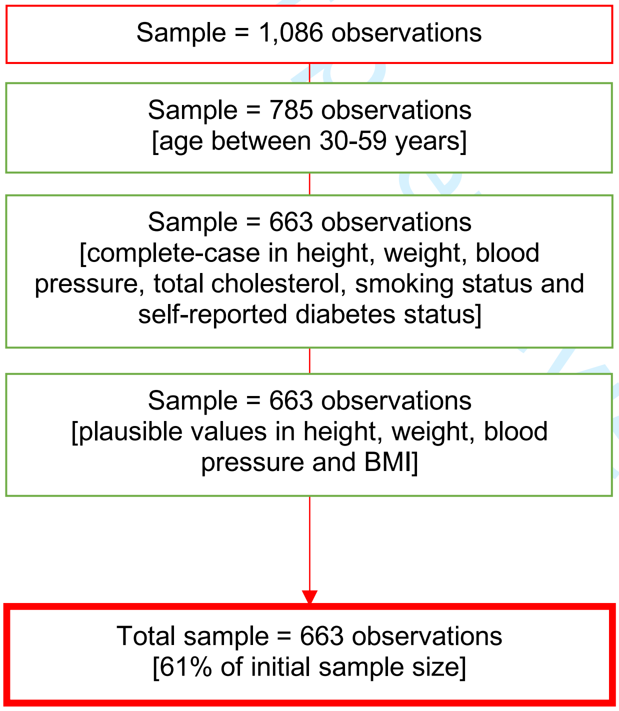

For peer review only

For peer review only - http://bmjopen.bmj.com/site/about/guidelines.xhtml

Supplementary Table 1. Summary statistics of the first and second systolic blood pressure (SBP) records in the overall sample

	First SBP record	Second SBP record
**Minimum value**	78	75
**1st quartile**	101	99
**Median**	109	108
**Mean**	110.33	109.54
**3rd quartile**	118	118
**Maximum value**	203	198

Supplementary Table 2. Sensitivity Analysis: Lin's concordance coefficient correlation showing agreement between laboratory and non-laboratory-based risk models according to the predictors in the 2019 WHO CVD risk models and urban/rural location

Variables	Categories	Lin's concordance coefficient correlation (95% CI)
**Sex**	**Men**	0.87 (0.84 - 0.89)
**Sex**	**Women**	0.85 (0.83 - 0.87)
**Age (years)**	**30-39**	0.87 (0.84 - 0.9)
**Age (years)**	**40-49**	0.74 (0.68 - 0.79)
**Age (years)**	**50-59**	0.83 (0.78 - 0.87)
**Body mass index category**	**Normal**	0.9 (0.87 - 0.92)
**Body mass index category**	**Overweight**	0.87 (0.85 - 0.9)
**Body mass index category**	**Obese**	0.86 (0.82 - 0.89)
**Smoking status**	**Smoker**	0.82 (0.73 - 0.88)
**Smoking status**	**Non-smoker**	0.86 (0.84 - 0.88)
**Diabetes status**	**With self−reported diabetes**	0.74 (0.63 - 0.82)
**Diabetes status**	**Not with self−reported diabetes**	0.91 (0.9 - 0.92)
**Urban or rural**	**Urban**	0.88 (0.86 - 0.9)
**Urban or rural**	**Rural**	0.86 (0.82 - 0.88)

For peer review only - http://bmjopen.bmj.com/site/about/guidelines.xhtml

Supplementary Table 3. Kappa statistics showing agreement between laboratory and non-laboratory-based risk scores in the overall sample

	Non-laboratory-based-risk category			
**Laboratory-based-risk category**	**0-5**	**5-9**	**10-19**	**kappa**
**0-5**	618	4	0	0.62
**5-9**	17	19	1	
**10-19**	0	3	1	

Supplementary Table 4. Kappa statistics showing agreement between laboratory and non-laboratory-based risk scores by sex

		Non-laboratory-based-risk category			
**Laboratory-based-risk category**	**Sex**	**0-5**	**5-9**	**10-19**	**kappa**
**0-5**	Men	251	4	0	0.7
**5-9**	Men	7	15	1	
**10-19**	Men	0	1	1	
**0-5**	Women	367	0	0	0.44
**5-9**	Women	10	4	0	
**10-19**	Women	0	2	0	

Supplementary Table 5. Kappa statistics showing agreement between laboratory and non-laboratory-based risk scores by age groups

		Non-laboratory-based-risk category			
**Laboratory-based-risk category**	**Age group**	**0-5**	**5-9**	**10-19**	**kappa**
**0-5**	30-39	236	0	0	0
**5-9**	30-39	1	0	0	
**10-19**	30-39	0	0	0	
**0-5**	40-49	216	1	0	0.45
**5-9**	40-49	4	2	1	
**10-19**	40-49	0	1	0	
**0-5**	50-59	166	3	0	0.65
**5-9**	50-59	12	17	0	
**10-19**	50-59	0	2	1	

For peer review only - http://bmjopen.bmj.com/site/about/guidelines.xhtml

Supplementary Table 6. Kappa statistics showing agreement between laboratory and non-laboratory-based risk scores by body mass index categories

		Non-laboratory-based-risk category			
**Laboratory-based-risk category**	**Body mass index category**	**0-5**	**5-9**	**10-19**	**kappa**
**0-5**	Normal	161	1	0	0.66
**5-9**	Normal	1	2	0	
**10-19**	Normal	0	0	0	
**0-5**	Obese	202	3	0	0.65
**5-9**	Obese	7	11	1	
**10-19**	Obese	0	2	1	
**0-5**	Overweight	255	0	0	0.55
**5-9**	Overweight	9	6	0	
**10-19**	Overweight	0	1	0	

Supplementary Table 7. Kappa statistics showing agreement between laboratory and non-laboratory-based risk scores by smoking status

		Non-laboratory-based-risk category			
**Laboratory-based-risk category**	**Smoking status**	**0-5**	**5-9**	**10-19**	**kappa**
**0-5**	Non-smoker	568	3	0	0.53
**5-9**	Non-smoker	12	9	0	
**10-19**	Non-smoker	0	1	0	
**0-5**	Smoker	50	1	0	0.67
**5-9**	Smoker	5	10	1	
**10-19**	Smoker	0	2	1	

Supplementary Table 8. Kappa statistics showing agreement between laboratory and non-laboratory-based risk scores by diabetes status

For peer review only - http://bmjopen.bmj.com/site/about/guidelines.xhtml

		Non-laboratory-based-risk category			
Laboratory-based-risk category	Diabetes status	0-5	5-9	10-19	kappa
**0-5**	Not with self−reported diabetes	592	4	0	0.71
**5-9**	Not with self−reported diabetes	7	15	1	
**10-19**	Not with self−reported diabetes	0	0	0	
**0-5**	With self−reported diabetes	26	0	0	0.36
**5-9**	With self−reported diabetes	10	4	0	
**10-19**	With self−reported diabetes	0	3	1	

Supplementary Table 9. Kappa statistics showing agreement between laboratory and non-laboratory-based risk scores by urban/rural location

		Non-laboratory-based-risk category			
Laboratory-based-risk category	Urban/Rural	0-5	5-9	10-19	kappa
**0-5**	Rural	224	1	0	0.53
**5-9**	Rural	4	3	0	
**10-19**	Rural	0	1	0	
**0-5**	Urban	394	3	0	0.64
**5-9**	Urban	13	16	1	
**10-19**	Urban	0	2	1	

Supplementary Figure 3. Sensitivity Analysis: Bland Altman plots showing agreement between laboratory and non-laboratory-based risk scores according to sex.

For peer review only - http://bmjopen.bmj.com/site/about/guidelines.xhtml

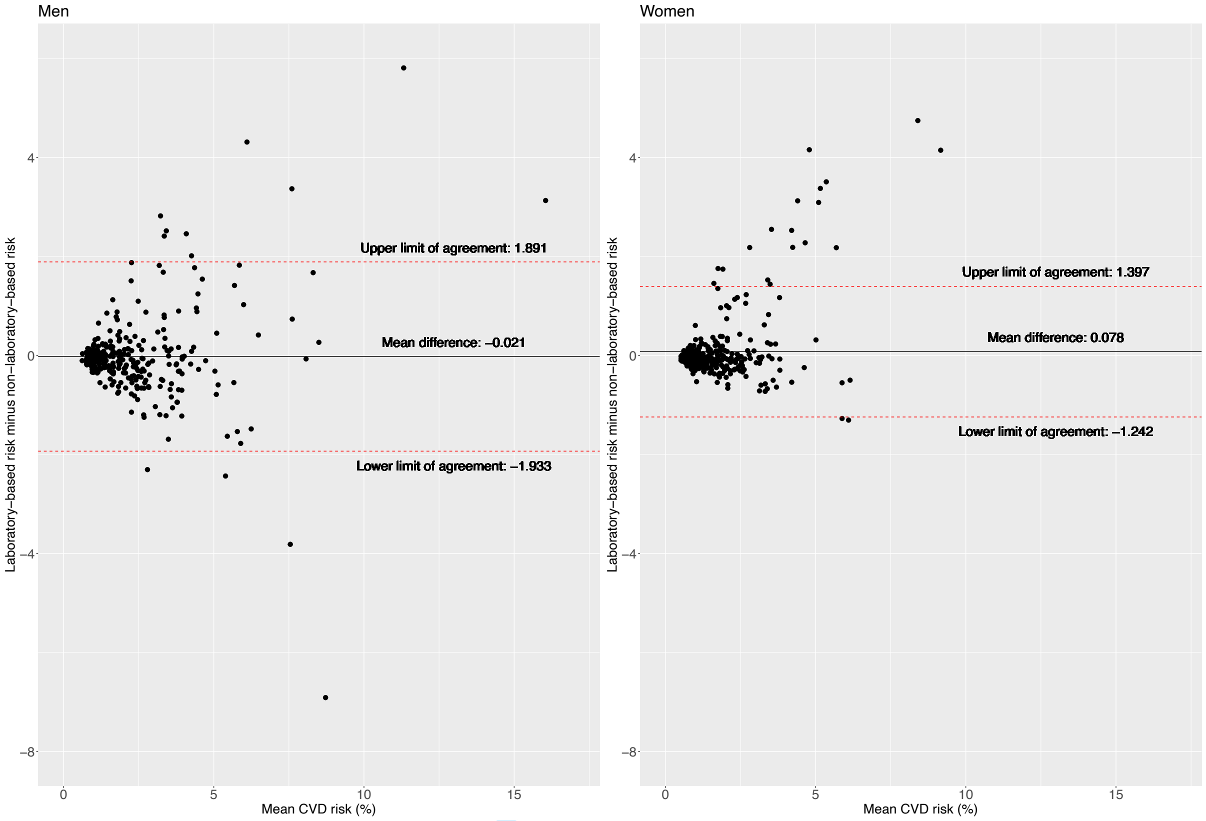

Supplementary Figure 4. Sensitivity Analysis: Bland Altman plots showing agreement between laboratory and non-laboratory-based risk scores according to age groups.

For peer review only - http://bmjopen.bmj.com/site/about/guidelines.xhtml

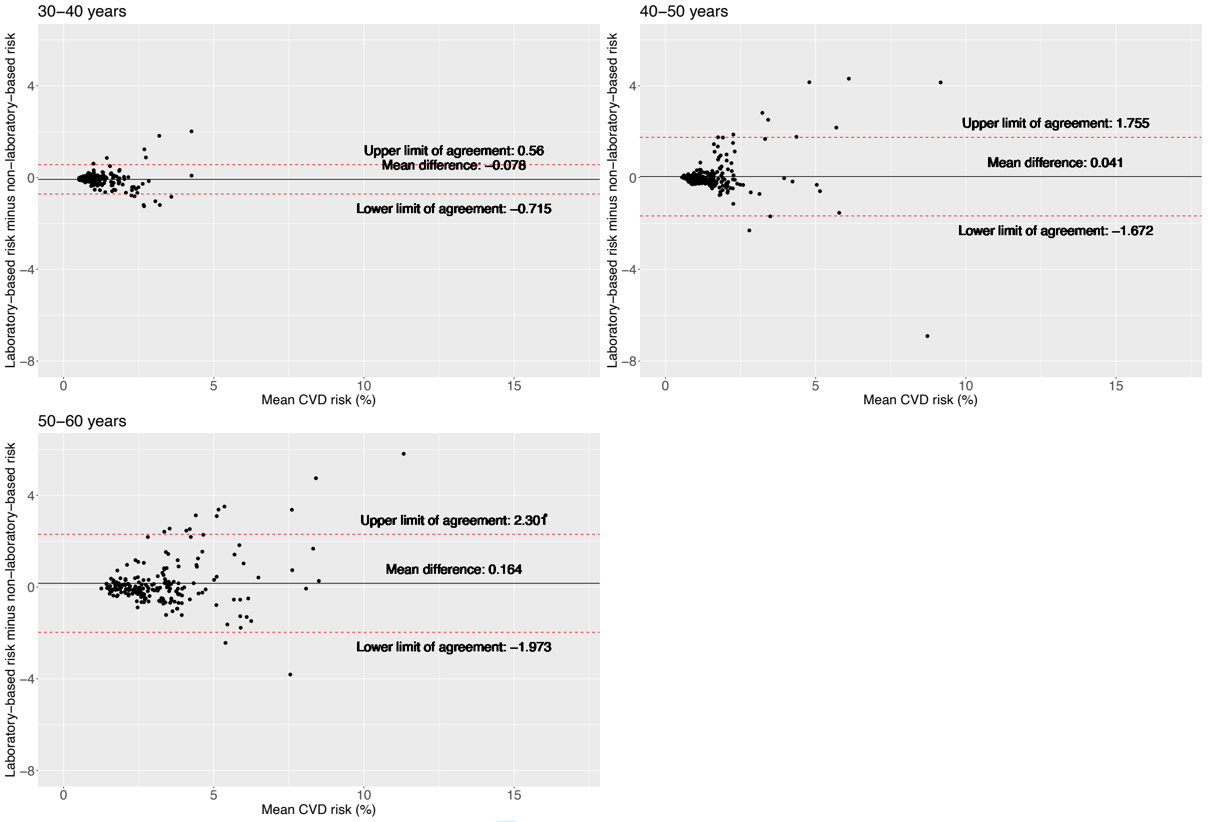

Supplementary Figure 5. Sensitivity Analysis: Bland Altman plots showing agreement between laboratory and non-laboratory-based risk scores according to body mass index categories.

For peer review only - http://bmjopen.bmj.com/site/about/guidelines.xhtml

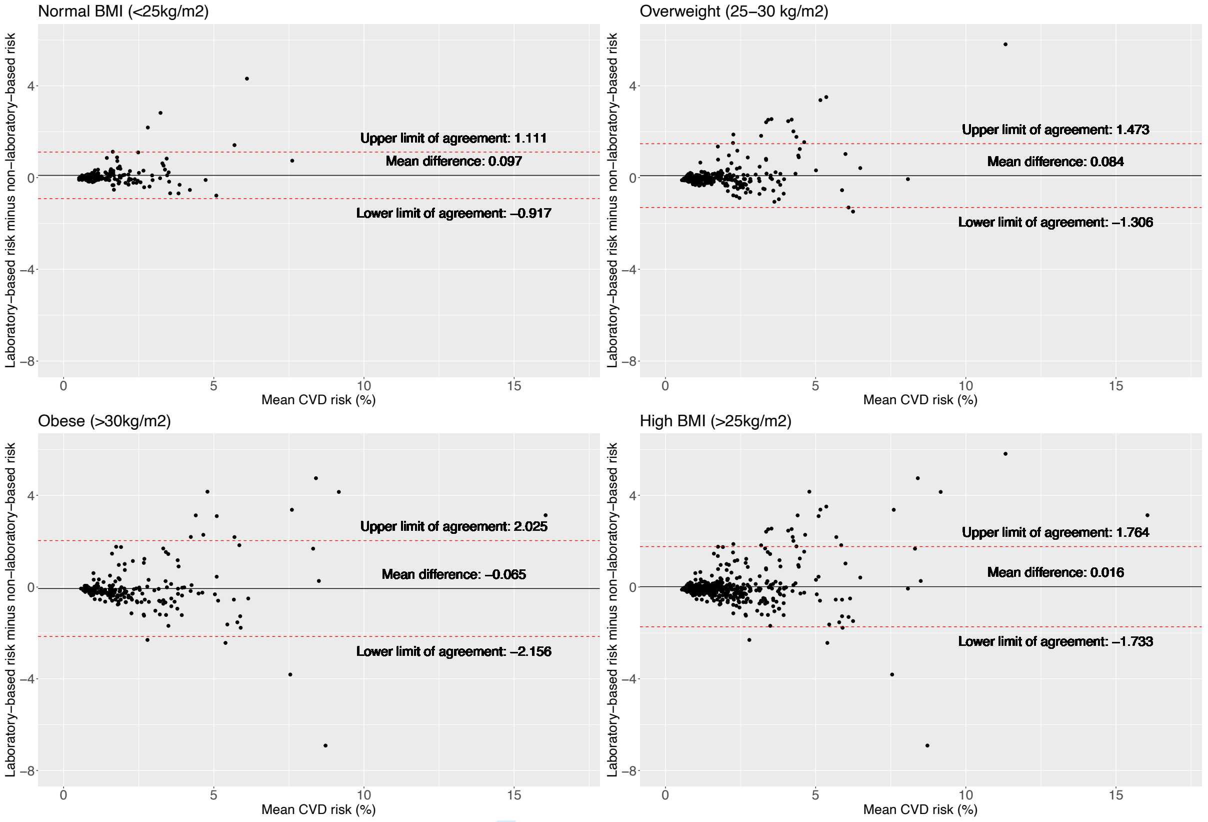

Supplementary Figure 6. Sensitivity Analysis: Bland Altman plots showing agreement between laboratory and non-laboratory-based risk scores according to smoking status.

For peer review only - http://bmjopen.bmj.com/site/about/guidelines.xhtml

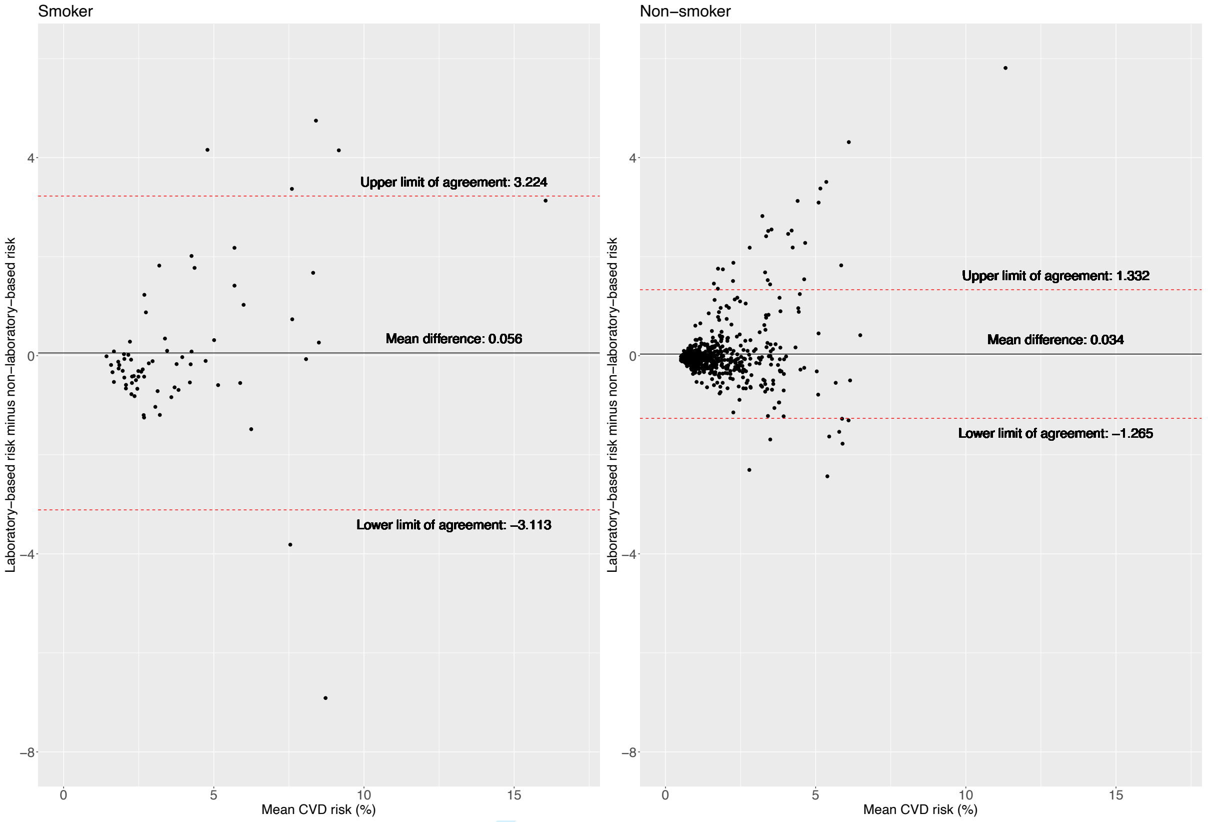

Supplementary Figure 7. Sensitivity Analysis: Bland Altman plots showing agreement between laboratory and non-laboratory-based risk scores according to self-reported diabetes status.

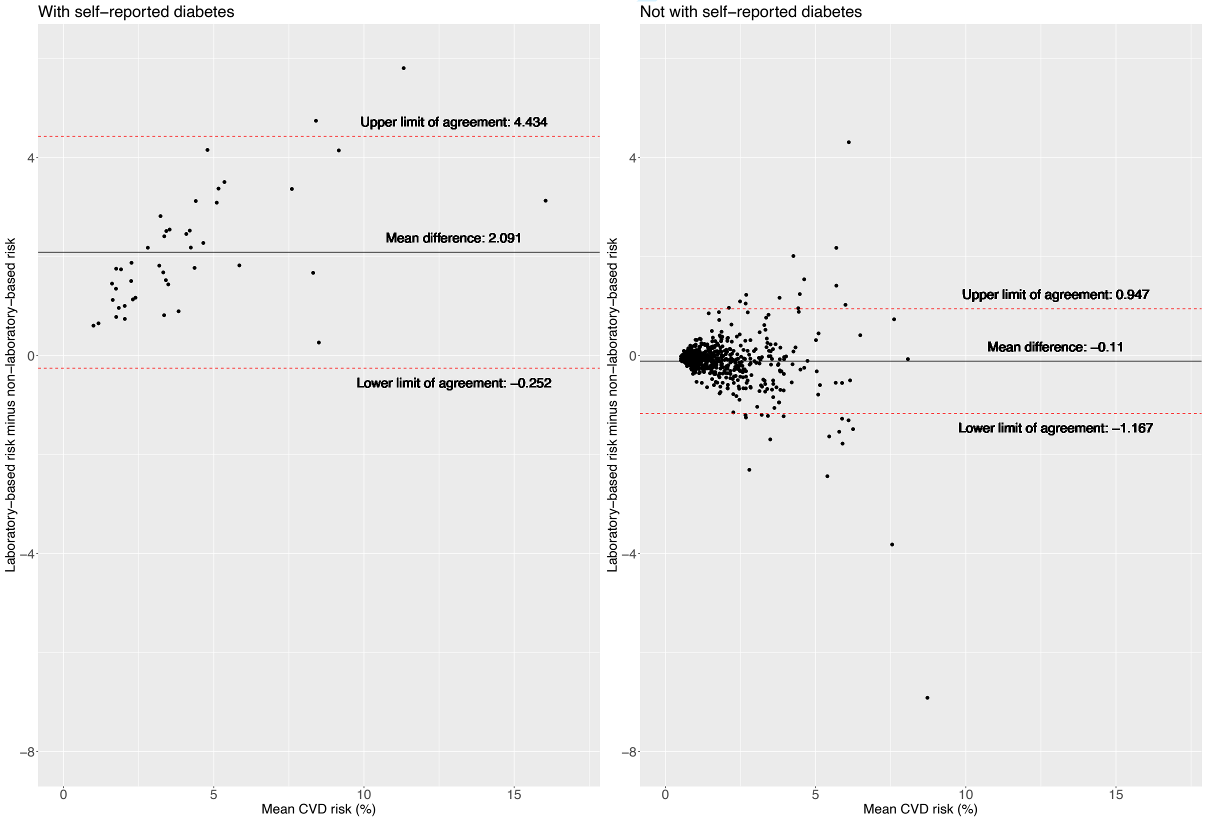

For peer review only - http://bmjopen.bmj.com/site/about/guidelines.xhtml

For peer review only

For peer review only - http://bmjopen.bmj.com/site/about/guidelines.xhtml

STROBE Statement—Checklist of items that should be included in reports of ***cross-sectional studies***

	Item No	Recommendation	Page No
**Title and abstract**	1	(*a*) Indicate the study's design with a commonly used term in the title or the abstract	3
		(*b*) Provide in the abstract an informative and balanced summary of what was done and what was found	3
**Introduction**			
Background/rationale	2	Explain the scientific background and rationale for the investigation being reported	4
Objectives	3	State specific objectives, including any prespecified hypotheses	5
**Methods**			
Study design	4	Present key elements of study design early in the paper	5
Setting	5	Describe the setting, locations, and relevant dates, including periods of recruitment, exposure, follow-up, and data collection	5-6
Participants	6	(*a*) Give the eligibility criteria, and the sources and methods of selection of participants	5-6
Variables	7	Clearly define all outcomes, exposures, predictors, potential confounders, and effect modifiers. Give diagnostic criteria, if applicable	6-7
Data sources/ measurement	8*	For each variable of interest, give sources of data and details of methods of assessment (measurement). Describe comparability of assessment methods if there is more than one group	*6-7*
Bias	9	Describe any efforts to address potential sources of bias	6
Study size	10	Explain how the study size was arrived at	5-6
Quantitative variables	11	Explain how quantitative variables were handled in the analyses. If applicable, describe which groupings were chosen and why	6-7
Statistical methods	12	(*a*) Describe all statistical methods, including those used to control for confounding	7-8
		(*b*) Describe any methods used to examine subgroups and interactions	7-8
		(*c*) Explain how missing data were addressed	5-6
		(*d*) If applicable, describe analytical methods taking account of sampling strategy	8
		(*e*) Describe any sensitivity analyses	6
**Results**			
Participants	13*	(a) Report numbers of individuals at each stage of study—eg numbers potentially eligible, examined for eligibility, confirmed eligible, included in the study, completing follow-up, and analysed	8
		(b) Give reasons for non-participation at each stage	Supp Fig 1
		(c) Consider use of a flow diagram	5
Descriptive data	14*	(a) Give characteristics of study participants (eg demographic, clinical, social) and information on exposures and potential confounders	8
		(b) Indicate number of participants with missing data for each variable of interest	Supp Fig 1
Outcome data	15*	Report numbers of outcome events or summary measures	

For peer review only

Main results	16	(*a*) Give unadjusted estimates and, if applicable, confounder-adjusted estimates and their precision (eg, 95% confidence interval). Make clear which confounders were adjusted for and why they were included	8-10
		(*b*) Report category boundaries when continuous variables were categorized	8-10
		(*c*) If relevant, consider translating estimates of relative risk into absolute risk for a meaningful time period	-
Other analyses	17	Report other analyses done—eg analyses of subgroups and interactions, and sensitivity analyses	10
**Discussion**			
Key results	18	Summarise key results with reference to study objectives	11
Limitations	19	Discuss limitations of the study, taking into account sources of potential bias or imprecision. Discuss both direction and magnitude of any potential bias	13
Interpretation	20	Give a cautious overall interpretation of results considering objectives, limitations, multiplicity of analyses, results from similar studies, and other relevant evidence	11-12
Generalisability	21	Discuss the generalisability (external validity) of the study results	11-12
**Other information**			
Funding	22	Give the source of funding and the role of the funders for the present study and, if applicable, for the original study on which the present article is based	2

*Give information separately for exposed and unexposed groups.

Note: An Explanation and Elaboration article discusses each checklist item and gives methodological background and published examples of transparent reporting. The STROBE checklist is best used in conjunction with this article (freely available on the Web sites of PLoS Medicine at http://www.plosmedicine.org/, Annals of Internal Medicine at http://www.annals.org/, and Epidemiology at http://www.epidem.com/). Information on the STROBE Initiative is available at www.strobe-statement.org.

For peer review only

For peer review only - http://bmjopen.bmj.com/site/about/guidelines.xhtml

BMJ Open

Agreement between the laboratory- and non-laboratory-based WHO cardiovascular risk charts: a cross-sectional analysis of a national health survey in Peru

Journal:	*BMJ Open*
Manuscript ID	bmjopen-2022-063289.R2
Article Type:	Original research
Date Submitted by the Author:	03-Oct-2022
Complete List of Authors:	Guzman-Vilca, Wilmer Cristobal; Universidad Peruana Cayetano Heredia, School of Medicine "Alberto Hurtado"; Universidad Peruana Cayetano Heredia, CRONICAS Centre of Excellence in Chronic Diseases<br>Quispe-Villegas, Gustavo A.; Universidad Peruana Cayetano Heredia, School of Medicine "Alberto Hurtado"<br>Váscones Román, Fritz Fidel; Universidad Peruana Cayetano Heredia, School of Medicine "Alberto Hurtado"<br>Bernabe-Ortiz, Antonio; Universidad Peruana Cayetano Heredia, CRONICAS Centre of Excellence in Chronic Diseases; Universidad Cientifica del Sur, School of Medicine<br>Carrillo-Larco, Rodrigo; Universidad Peruana Cayetano Heredia, CRONICAS Centre of Excellence in Chronic Diseases; Imperial College London, Department of Epidemiology and Biostatistics
**Primary Subject Heading**:	Cardiovascular medicine
Secondary Subject Heading:	Cardiovascular medicine
Keywords:	Cardiac Epidemiology < CARDIOLOGY, Adult cardiology < CARDIOLOGY, EPIDEMIOLOGY

SCHOLARONE™
Manuscripts

I, the Submitting Author has the right to grant and does grant on behalf of all authors of the Work (as defined in the below author licence), an exclusive licence and/or a non-exclusive licence for contributions from authors who are: i) UK Crown employees; ii) where BMJ has agreed a CC-BY licence shall apply, and/or iii) in accordance with the terms applicable for US Federal Government officers or employees acting as part of their official duties; on a worldwide, perpetual, irrevocable, royalty-free basis to BMJ Publishing Group Ltd ("BMJ") its licensees and where the relevant Journal is co-owned by BMJ to the co-owners of the Journal, to publish the Work in this journal and any other BMJ products and to exploit all rights, as set out in our licence.

The Submitting Author accepts and understands that any supply made under these terms is made by BMJ to the Submitting Author unless you are acting as an employee on behalf of your employer or a postgraduate student of an affiliated institution which is paying any applicable article publishing charge ("APC") for Open Access articles. Where the Submitting Author wishes to make the Work available on an Open Access basis (and intends to pay the relevant APC), the terms of reuse of such Open Access shall be governed by a Creative Commons licence – details of these licences and which Creative Commons licence will apply to this Work are set out in our licence referred to above.

Other than as permitted in any relevant BMJ Author's Self Archiving Policies, I confirm this Work has not been accepted for publication elsewhere, is not being considered for publication elsewhere and does not duplicate material already published. I confirm all authors consent to publication of this Work and authorise the granting of this licence.

For peer review only

Agreement between the laboratory- and non-laboratory-based WHO cardiovascular risk charts: a cross-sectional analysis of a national health survey in Peru

Wilmer Cristobal Guzman-Vilca [1,2,3]

Gustavo A. Quispe-Villegas [1,3]

Fritz Fidel Vascones-Roman [1.2,3]

Antonio Bernabe-Ortiz [2,5]

Rodrigo M. Carrillo-Larco [2,4]

1. School of Medicine "Alberto Hurtado", Universidad Peruana Cayetano Heredia, Lima, Peru.
2. CRONICAS Centre of Excellence in Chronic Diseases, Universidad Peruana Cayetano Heredia, Lima, Peru.
3. Sociedad Científica de Estudiantes de Medicina Cayetano Heredia (SOCEMCH), Universidad Peruana Cayetano Heredia, Lima, Peru.
4. Department of Epidemiology and Biostatistics, School of Public Health, Imperial College London, London, UK.
5. Universidad Cientifica del Sur, Lima, Peru.

Correspondence author:

Rodrigo M Carrillo-Larco, MD

Department of Epidemiology and Biostatistics

School of Public Health

Imperial College London

rcarrill@ic.ac.uk

For peer review only

ABSTRACT

Objective: To determine the agreement between the cardiovascular disease (CVD) risk predictions computed with the World Health Organization (WHO) non-laboratory-based model and laboratory-based model in a nationally-representative sample of Peruvian adults.

Design: Cross-sectional analysis of a national health survey.

Methods: Absolute CVD risk was computed with the 2019 WHO laboratory- and non-laboratory-based models. The risk predictions from both models were compared with Bland Altman plots, Lin's concordance coefficient correlation (LCCC) and kappa statistics, stratified by sex, age, body mass index categories, smoking and diabetes status.

Results: 663 people aged 30-59 years were included in the analysis. Overall, there were no substantial differences between the mean CVD risk computed with the laboratory-based model 2.0% (95% CI: 1.8%; 2.2%) and the non-laboratory-based model 2.0% (95% CI: 1.8%; 2.1%). In the Bland Altman plots, the limits of agreement were the widest among people with diabetes (-0.21; 4.37) compared with people without diabetes (-1.17; 0.95). The lowest agreement as per the LCCC was also seen in people with diabetes (0.74 (95% CI: 0.63; 0.82)), the same was observed with the kappa statistic (kappa=0.36). In general, agreement between the scores was appropriate in terms of clinical significance.

Conclusions: The absolute cardiovascular predicted risk was similar between the laboratory-based and non-laboratory-based 2019 WHO cardiovascular risk models. Pending validation from longitudinal studies, the non-laboratory-based model (instead of the laboratory-based) could be used when assessing CVD risk in Peruvian population.

Key words: cardiovascular diseases; risk assessment; health metrics

For peer review only

STRENGTHS AND LIMITATIONS OF THIS STUDY

- This analysis provided the first evidence of the agreement between the 2019 WHO CVD risk laboratory-based and non-laboratory-based models.
- We leveraged on the most recent nationally representative survey that included blood biomarkers in Peru.
- Our study population was young, mostly women, and with overall low absolute cardiovascular predicted risk. No one in the study population had an absolute cardiovascular risk ≥20%.
- We assumed all participants were free of CVD to use the 2019 WHO CVD risk score, as information regarding history of cardiovascular events was not reported.

INTRODUCTION

Cardiovascular diseases (CVD) are the main cause of death globally.[1] In 2019, CVD caused ~18.5 million deaths in adults, representing 36% of all global deaths.[2,3] Furthermore, CVD impose a huge burden in low- and middle-income countries (LMICs),[4] where deaths from CVD occur at younger ages compared to high-income countries (HICs).[1] However, CVD can be prevented and managed through a combination of population- and individual-level interventions;[5] for the latter, the identification of individuals at high cardiovascular risk is a cornerstone in the prevention of CVD. In this line, the World Health Organization (WHO) and the Pan American Health Organization (PAHO),[6] alongside several clinical guidelines,[7–9] recommend CVD risk stratification with CVD risk prediction models to inform evidence-based treatment.

CVD risk prediction models identify people who would benefit the most of preventive interventions (e.g., statin therapy).[10] Although there are several CVD risk prediction models,[11] these were mostly developed in HICs limiting their application in LMICs where they would need recalibration to deliver accurate predictions to guide treatment allocation. To overcome this limitation, the WHO convened a global effort to derive, calibrate and validate new CVD risk prediction models for all world regions.[12] Two WHO CVD risk prediction models were developed: a laboratory-based

For peer review only

model and a non-laboratory-based model. Because laboratory biomarkers (e.g., total cholesterol) may not be available in primary health care centres in LMICs limiting the use of laboratory-based CVD risk prediction models,[13] the non-laboratory-based model arises as a handy tool for clinicians in LMICs. Similarly, CVD risk prediction models are used to monitor the prevalence of high CVD risk and treatment coverage (i.e., people at high CVD receiving treatment).[14] In this context, countries conducting national or large population-based health surveys without lipid biomarkers, could benefit from the non-laboratory-based models. Peru, for example, does not have regular national health surveys including total cholesterol, but Peru has a yearly national health survey including anthropometrics, blood pressure, and health questionnaires. In Peru, and other similar LMICs, it would not be possible to monitor the burden of high CVD risk with a laboratory-based model and the non-laboratory-based model rises as the only alternative. Nonetheless, evidence regarding the agreement between the WHO laboratory-based and non-laboratory-based models in countries from Latin America is missing.[12] Whether the WHO non-laboratory-based model delivers predictions similar to those of the WHO laboratory-based model remains unknown in Latin America. However, clinicians need this evidence to inform their choice (non-laboratory-based vs laboratory-based model), and to interpret the results of the non-laboratory-based model under the assumption that the laboratory-based model is the gold standard.[12] To provide this evidence for practitioners in Peru, we determined the agreement between the risk predictions computed with the non-laboratory-based model and laboratory-based model in a nationally-representative sample of Peruvian adults.

METHODS

Data sources

This is a cross-sectional study of a national survey conducted by the National Centre for Food and Nutrition (CENAN, for its acronym in Spanish) of Peru. CENAN's survey was conducted between 2017-2018 on a nationally-representative sample of Peruvian adults aged between 18-59 years.[15] Of note, this is the most recent nationally-representative survey conducted in Peru that included blood biomarkers (e.g., lipid profile). CENAN's survey adhered to ethical guidelines and followed a standardised protocol that has been published elsewhere.[15] Each participant

For peer review only

was informed about all procedures and techniques used in the survey; also, participants could have left the study at any time and their personal information was kept confidential.[15]

The CENAN's survey sample was computed using the formula shown in Supplementary Figure 1 and followed a probabilistic sampling design approach with two stages.[15] First, clusters were randomly selected considering three strata: 1) Urban areas except Lima city, 2) Rural areas, and 3) Lima city. Then, households (of adults aged 18-59 years living in) were randomly selected within each cluster. To be selected for the survey sample, participants had to fulfil the following inclusion criteria: 1) Adults aged 18-59 years, and 2) Fasting 9-12 hours for blood biomarkers. The following participants were excluded: 1) Pregnant and postpartum women, 2) Adults taking medication that could alter glucose and lipid profiles, 3) Adults with congenital diseases that could limit anthropometrics measurement (e.g., Down syndrome).

Study population

We analysed a complete-case sample regarding all the laboratory- and office-based 2019 ten-year WHO CVD risk score variables (see Variables section). We studied men and women aged between 30-59 years. The younger age limit was decided because CVD risk models are not recommended in younger individuals; the older age limit was decided because of the survey design. A flowchart of data cleaning is shown in Supplementary figure 2. We did not apply other selection criteria.

Although the 2019 ten-year WHO CVD risk models were developed for people aged 40-80 years,[12] for people aged <40 years we assumed they had 40 years for the absolute CVD risk computation. This is consistent with the Package of essential noncommunicable (PEN) disease interventions for primary health care in low-resource settings,[16] and was also done in a recent global work.[17]

Variables

We calculated the ten-year CVD risk at the individual-level following the laboratory- and non-laboratory-based 2019 ten-year WHO CVD risk model.[12] We used the *whocvdrisk* command in

For peer review only

STATA, which was developed by the authors of the 2019 ten-year WHO CVD risk charts.[18] For the laboratory-based model, scores were calculated based on: age (years), current smoking status (yes/no), systolic blood pressure (SBP, mmHg), history of self-reported diabetes diagnosis (yes/no), and total cholesterol (mmol/L).[12] For the non-laboratory-based model, we used age (years), current smoking status (yes/no), systolic BP (mmHg) and body mass index (BMI, kg/m^2).[12]

The CENAN's survey collected anthropometrics and three BP measurements which were taken by trained fieldworkers following a standard protocol.[15] We computed BMI using measured weight (kg) divided by the square of height (meters); for descriptive purposes, we classified BMI in three levels: normal weight (BMI <25 kg/m^2), overweight (BMI ≥25-29.9 kg/m^2), and obesity (BMI ≥30 kg/m^2). BMI records outside the range 10-80 kg/m^2 were discarded. As the third BP measurement was only available in few participants (<2% of the initial sample), we used the second SBP measurement only (i.e., the first and third SBP records were discarded in the main analysis). Of note, there were no substantial differences between the first and second SBP records (Supplementary table 1); nonetheless, we performed a sensitivity analysis using the mean SBP of first and second SBP records. We discarded any SBP records outside the range 70-270 mmHg.

For people who self-reported being under antihypertensive treatment, we used the pre-treatment SBP; this is consistent with the PEN protocol and with a previous global work.[16,17]. Pre-treatment SBP was computed as: pre-treatment systolic blood pressure = (current systolic blood pressure-6.3)/0.9.[19] Conversely, for those not taking antihypertensive treatment, we used the recorded SBP as was.

We defined current smoker with one question coded as no versus yes: *do you currently smoke any tobacco product such as cigarettes, cigars or pipes?* Self-reported information about a prior history of diabetes was assessed by a question also coded as no versus yes: *have you ever been told by a physician or another healthcare worker that you have high blood sugar or diabetes?*

For peer review only

Total cholesterol was obtained via enzymatic colorimetric method.[15] Because CENAN's survey data had total cholesterol in mg/dl, these values were divided by 38.67 to obtain total cholesterol in mmol/L.

Statistical analysis

We determined the agreement between the absolute CVD risk predicted with the WHO laboratory- and non-laboratory-based models following three methods: Bland Altman plots, Lin's concordance coefficient correlation (LCCC), and kappa statistic. We considered the absolute CVD risk as a continuous variable, and the agreement between both models was examined using Bland Altman plots and the LCCC. Furthermore, we considered the CVD risk as a categorical variable, and divided into three groups: <5%, 5-9%, 10-19%; because there were no observations in the high-risk category (CVD risk ≥20%), agreement was not examined in this group. For these categories, we evaluated the agreement using the kappa statistic. For the Bland Altman plots, LCCC, and the kappa statistic, results were stratified by sex, 10-year age groups, BMI categories, smoking status, self-reported diabetes diagnosis and urban/rural location.

In the Bland Altman plots, the risk difference between the laboratory- and non-laboratory-based absolute cardiovascular predicted risk was plotted on the vertical axis, and the mean of both scores on the horizontal axis.[20] As the true risk of CVDs at the individual-level is uncertain, the mean of both scores is the best available estimate.[20,21] The 95% of the limit of agreement was represented by the mean difference of both scores ± two standard deviations; this limit provides an interval in which 95% of the differences between both scores would be expected to lie.[20] The LCCC between the laboratory- and non-laboratory-based absolute cardiovascular predicted risks was also evaluated. The agreement based on the LCCC ranges between -1 and 1, with 1 suggesting a perfect agreement. The categorical agreement was evaluated using the Kappa statistic. Kappa <0 indicated less than chance agreement, and values 0.01–0.20, 0.21–0.40, 0.41–0.60, 0.61–0.80, and 0.81–0.99 represented slight, fair, moderate, substantial, and almost perfect agreement, respectively.[22]

For peer review only

All analyses code were conducted with R (version 4.0.3) and STATA (version 17.0, College Station, Texas 77845, USA). Population characteristics along with their 95% confidence interval (95% CI) were summarized accounting for the complex survey design of the CENAN's survey.[15]

Patient and public involvement

No patient involved.

RESULTS

Our pooled dataset included 663 participants (Supplementary figure 2). The mean age was 44.0 years (95% CI: 43.2; 44.7) and the proportion of men was 41.5%. The mean SBP was 108.9 mmHg (95% CI: 107.5; 110.3 mmHg) and the mean BMI was 28.8 kg/m^2 (95% CI: 28.3; 29.2 kg/m^2). The proportion of people with overweight was 41.0% (95% CI: 36.6; 45.6 %), whereas 35.9% (95% CI: (31.6; 40.5%) of the population were obese. The mean total cholesterol was 4.9 mmol/L (95% CI: 4.8; 5.0 mmol/L), 11.7% (95% CI: 8.8; 15.3%) of the population were smokers and 7.1% (95% CI: 5.0%; 9.8%) had diabetes (Table 1).

Table 1. Weighted distribution of the predictors in the 2019 WHO CVD risk models, overall and by sex.

	Total	Men	Women
**Sample size**	663	280	383
**Age (mean and 95% CI, years)**	44 (43.2-44.7)	44.2 (43-45.4)	43.8 (42.9-44.7)
**Proportion of people aged 30-39 years (95% CI, %)**	35.8 (31.6-40.3)	37.2 (31-43.9)	34.8 (29.4-40.7)
**Proportion of people aged 40-49 years (95% CI, %)**	34 (29.8-38.4)	29.5 (24-35.7)	37.1 (31.5-43.1)
**Proportion of people aged 50-59 years (95% CI, %)**	30.2 (26.3-34.5)	33.3 (27.1-40)	28 (23.5-33.1)
**Systolic blood pressure (mean and 95% CI, mmHg)**	108.9 (107.5-110.3)	116.1 (113.8-118.4)	103.8 (102.3-105.3)
**Diastolic blood pressure (mean and 95% CI, mmHg)**	71.9 (71-72.8)	74.6 (73.1-76.1)	70 (69-71)
**Body mass index (mean and 95% CI, kg/m2)**	28.8 (28.3-29.2)	28.3 (27.6-29)	29.1 (28.5-29.7)
**Proportion of people with normal weight (95% CI, %)**	23.1 (19.5-27.2)	24.5 (19.5-30.4)	22.1 (17.2-27.9)

For peer review only

**Proportion of people with overweight (95% CI, %)**	41 (36.6-45.6)	45.3 (38.9-52)	37.9 (31.9-44.3)
**Proportion of people with obesity (95% CI, %)**	35.9 (31.6-40.5)	30.1 (24.1-36.9)	40 (34-46.2)
**Total cholesterol (mean and 95% CI, mmol/L)**	4.9 (4.8-5)	4.8 (4.6-5)	4.9 (4.8-5.1)
**Proportion of smokers (95% CI, %)**	11.7 (8.8-15.3)	21.1 (15.8-27.6)	5 (3-8.3)
**Proportion of people with diabetes (95% CI, %)**	7.1 (5-9.8)	7.8 (4.7-12.6)	6.5 (4.3-9.9)
**Laboratory-based CVD risk score (mean and 95% CI, %)**	2 (1.8-2.2)	2.6 (2.3-2.9)	1.6 (1.4-1.7)
**Non-laboratory-based CVD risk score (mean and 95% CI, %)**	2 (1.8-2.1)	2.7 (2.4-3)	1.5 (1.4-1.6)

For peer review only

Absolute cardiovascular risk according to the 2019 WHO cardiovascular risk models

Overall, there were no substantial differences between the mean absolute cardiovascular risk computed with the laboratory-based and non-laboratory-based models (Table 1). The mean absolute cardiovascular risk was 2.0% (95% CI: 1.8%; 2.2%) according to the laboratory-based model, and 2.0% (95% CI: 1.8%; 2.1%) according to the non-laboratory-based model. In both models, the mean absolute cardiovascular risk was higher in men than women. The sensitivity analysis (using the mean of two SBP records) yielded the same findings in the overall sample: 2.0% (95% CI: 1.8; 2.2%) in the laboratory-based-model and 2.0 (95% CI: 1.8; 2.1%) in the non-laboratory-based model.

Mean difference between risk predictions

Overall, the mean difference between the laboratory-based and the non-laboratory-based models was 0.03% (95% CI: -0.03%; 0.10%). According to sex, the mean difference between models was -0.02% (95% CI: -0.14%; 0.09%) in men, and 0.08% (95% CI: 0.01%; 0.15%) in women. According to age, the mean difference between models was -0.07% (95% CI: -0.12%; 0.04%) in people aged 30-39 years, 0.04% (95% CI: -0.08%; 0.15%) in people aged 40-49 years, and 0.17% (95% CI: 0.02%; 0.32%) in people aged 50-59 years. Stratified by BMI categories, the mean difference was 0.10% (95% CI: 0.02%; 0.18%) in people with normal weight, 0.08% (95% CI: 0.00%; 0.17%) in people with overweight, and -0.07% (95% CI: -0.21%; 0.07%) in people with obesity. The mean difference was 0.05% (95% CI: -0.34%; 0.44%) in smokers and 0.03% (95% CI: -0.02%; 0.09%) in non-smokers. According to self-reported diabetes status, mean difference was 2.08% (95% CI: 1.73%; 2.44%) in people with self-reported diabetes, and -0.11% (95% CI: -0.15%; 0.07%) in people without self-reported diabetes. The sensitivity analysis provided similar results across all variables; for example, the largest mean difference was also observed in people with self-reported diabetes (2.09% (95% CI: 1.73; 2.45%).

Bland-Altman plots and limits of agreement.

The limit of agreement was slightly narrower for women (-1.23; 1.39) compared to men (-1.93; 1.89) (Figure 1). The limit of agreement widened with older ages and higher BMI levels; for

For peer review only - http://bmjopen.bmj.com/site/about/guidelines.xhtml

example, the limit of agreement was narrower in people aged 30-39 years (-0.72; 0.56) compared to those aged 50-59 years (-1.96; 2.29) (Figure 2), and for people with normal weight (-0.90; 1.09) compared to those with obesity (-2.15; 2.02) (Figure 3). According to smoking and self-reported diabetes status, the limit of agreement was wider in those who had the condition. For example, the limit of agreement in smokers (-3.13; 3.24) was wider compared to non-smokers (-1.25; 1.32) (Figure 4). Similarly, in people with self-reported diabetes (-0.21; 4.37) the limit of agreement was wider compared to people without self-reported diabetes (-1.17; 0.95) (Figure 5). Notably, the sensitivity analysis resulted in similar limits of agreement in all variables (Supplementary figures 3-7).

Agreement by Lin's concordance coefficient correlation

The overall agreement between scores as per the LCCC was 0.87 (95% CI: 0.85; 0.89), and it was virtually the same in men (0.87 (95% CI 0.84; 0.89)) and women (0.85 (95% CI 0.82; 0.87)). Across age groups, the highest agreement was seen in the 30-39 age group (0.87 (95% CI 0.84; 0.9)). Across BMI categories, it was the normal BMI category which had the highest agreement (0.90 (95% CI 0.87; 0.92)). Overall, the lowest agreement values were seen across smokers (0.81 (95% CI 0.72; 0.88)), those aged 40-49 years (0.74 (95% CI 0.67; 0.79)), and those with self-reported diabetes (0.74 (95% CI 0.63; 0.82)) (Table 2). Similar results were seen in the sensitivity analysis (Supplementary table 2).

Table 2. Lin's concordance coefficient correlation showing agreement between laboratory and non-laboratory-based risk models according to the predictors in the 2019 WHO CVD risk models and urban/rural location

Variables	Categories	Lin's concordance coefficient correlation (95% CI)
**Sex**	**Men**	0.87 (0.84 - 0.89)
	**Women**	0.85 (0.82 - 0.87)
**Age (years)**	**30-39**	0.87 (0.84 - 0.9)
	**40-49**	0.74 (0.67 - 0.79)
	**50-59**	0.83 (0.78 - 0.86)
**Body mass index category**	**Normal**	0.9 (0.87 - 0.92)

For peer review only - http://bmjopen.bmj.com/site/about/guidelines.xhtml

	**Overweight**	0.87 (0.85 - 0.9)
	**Obese**	0.86 (0.82 - 0.89)
**Smoking status**	**Smoker**	0.81 (0.72 - 0.88)
	**Non-smoker**	0.86 (0.84 - 0.88)
**Diabetes status**	**With self−reported diabetes**	0.74 (0.63 - 0.82)
	**Not with self−reported diabetes**	0.91 (0.9 - 0.92)
**Urban or rural**	**Urban**	0.88 (0.85 - 0.9)
	**Rural**	0.85 (0.82 - 0.88)

Categorical agreement

In the overall population, there was a slightly larger number of people categorized as having an absolute CVD risk of 5-9% and 10-19% with the laboratory-based-model compared with the non-laboratory-based model (Supplementary table 3). For example, the laboratory-based model categorized 37 people in the 5-9% CVD risk category, whereas the non-laboratory-based model categorized 26 people. Overall, the agreement between risk categories was substantial (kappa=0.62), and it was better for men (kappa=0.70) compared to women (kappa=0.44) (Supplementary table 4). Of note, the lowest agreement between risk categories was observed among people with self-reported diabetes (kappa=0.36): out of 14 people with self-reported diabetes in the 5-9% CVD risk category following the laboratory-based-model, 4 were placed in the same category following the non-laboratory-based model, as the rest were placed in the 0-5% CVD risk category. The categorical agreement according to all variables is presented in Supplementary tables 3-9.

DISCUSSION

Main findings

In this work, we evaluated the agreement between the CVD risk estimates predicted with the 2019 WHO ten-year laboratory-based and non-laboratory-based models in a nationally-representative sample of Peruvian adults. The mean absolute predicted CVD risk according to both models in the general population was virtually the same. In addition, we found that the limits of agreement between both models increased with a higher CVD risk; for instance, the limits of agreement were

For peer review only - http://bmjopen.bmj.com/site/about/guidelines.xhtml

wider in smokers and people with self-reported diabetes. We observed good agreement between the laboratory-based and non-laboratory-based models in terms of clinical significance.[21,23] These findings suggest that, in a population with a similar profile to that in this study, practitioners could use either the laboratory-based or non-laboratory-based models. Although the difference is very small, careful interpretation could be needed for people with cardiovascular risk factors: obesity, smokers and people with self-reported diabetes, amongst whom the difference was slightly larger than in their peers without these risk factors.

Public health implications

We provided insights about the applicability of the 2019 WHO non-laboratory-based model in the Peruvian population. This evidence is relevant in terms of clinical practice and public health in Peru and other similar countries (e.g., Andean Latin America), because it informs whether the predictions based on the laboratory-based and non-laboratory-based CVD risk models are equivalent. If so, either of these models could be used without substantial bias, hence supporting the use of the non-laboratory-based model when blood biomarkers are not available. The latter is of special importance in LMICs like Peru, where laboratory measurements are not always available in primary health centres, which represent >98% of all health care facilities in Peru.[24]

According to the three statistical analyses we implemented (Bland-Altman plots, Lin's concordance coefficient correlations; and kappa statistics), our results suggest that the agreement between the laboratory-based and non-laboratory-based models was appropriate among Peruvians with low CVD risk and younger than 60 years. In other words, our results suggest that the laboratory-based and non-laboratory-based models provide similar predictions and may therefore be used interchangeably as needed, though the profile of our study population ought to be considered when extracting or implementing our findings into clinical practice and public health. Of note, the small number of participants in some variables of interest (e.g., only 44 participants had self-reported diabetes) could have explained the broader limits of agreement in our results. Further studies should include a larger number of participants to further confirm whether limits of agreement are wider according to smoking and diabetes status.

For peer review only - http://bmjopen.bmj.com/site/about/guidelines.xhtml

Research in context

The study most comparable to ours evaluated the agreement between the Framingham 10-year CVD risk laboratory and non-laboratory models on a population aged 40-75 years in southern Iran.[23] They found the mean CVD risk following the non-laboratory-based model (9.4%) was higher than the laboratory-based model (6.7%).[23] Additionally, their limits of agreement between both Framingham models in people <60 years old were wider compared to ours in both men (-1.9-1.9 by our estimates versus -2.5%-8.9% by Rezaei et al.[23]) and women (-1.2-1.4 by our estimates versus -2.3%-4.6% by Rezaei et al.[23]). This could be explained by the fact that Rezaei et al.[23] included an older population, which tend to have higher levels of CVD risk factors and therefore a higher absolute CVD risk. As limits of agreement between two models tend to widen with higher CVD risk,[21,23] our limits of agreement would presumably be wider if we had studied a similar population to that of the work by Rezaei et al.[23] The differences between our results could be further explained by the CVD risk score herein used. We used the 2019 WHO CVD risk models,[12] whereas Rezaei et al. used the Framingham risk scores. The Framingham risk score was developed for a more specific population (Caucasians in the US),[25] yet the 2019 WHO CVD risk model was developed and recalibrated for a global use (e.g., those living in LMICs).[12]

The agreement between the 2019 WHO laboratory- and non-laboratory-based model was also explored in the global work convened by the WHO.[12] They applied the two models to WHO STEPS surveys, and compared the proportion of people categorized at different levels of predicted CVD risk.[12] Overall, they found moderate agreement between both models, and their discrepancy was attributed to poor performance of the non-laboratory-based model in people with diabetes.[12] This finding is consistent with our results because we found the widest limits of agreement, the lowest LCCC, and the lowest categorical agreement in people with self-reported diabetes. When possible, it would seem reasonable to use the laboratory-based model in those whose have diabetes.

For peer review only - http://bmjopen.bmj.com/site/about/guidelines.xhtml

Potential explanations

In our study, diabetes status was only included in the laboratory-based model (and not in the non-laboratory-based model) and reasonably, we observed the lowest agreement between both models in those with diabetes. That is, in people with diabetes, the non-laboratory-based model underestimated the absolute CVD risk computed following the laboratory-based model. Probably because people with diabetes are already at high CVD risk and the non-laboratory model, we used without diabetes information would underestimate the absolute risk.

Strengths and limitations

To the best of our knowledge, we provided the first evidence of the agreement between the 2019 WHO CVD risk laboratory-based and non-laboratory-based models; furthermore, we leveraged on a nationally-representative survey conducted in a LMIC.[26] Different CVD risk equations have been created but none has proved to produce reliable estimates for LMICs. The 2019 WHO CVD risk charts were adapted for LMICs using extensive datasets for its derivation, recalibration, and validation, which brings them several advantages over previous risk charts. Nonetheless, this study has also limitations. First, our study population was young (30-59 years), mostly women (58%), and with overall low absolute cardiovascular predicted risk. This led to the observation that no one in the study population had an absolute cardiovascular risk ≥20%. Thus, we could only draw conclusions for people within the low and medium CVD risk range, and with a similar demographic and risk factor profile. We acknowledge that further subgroup analysis could be relevant, for example by diabetes status. However, because of data availability and the reduced number of observations in some groups, this subgroup analyses would be impossible to conduct. Future work in Peru and Latin America should verify our results with a larger, older and more diverse population. Second, as CENAN's survey did not include history of cardiovascular events, we assumed all participants were free of CVD to use the 2019 WHO CVD risk score. This approach could have led to higher absolute cardiovascular risk because people who have had a cardiovascular event (e.g., myocardial infarction) are at higher risk of another cardiovascular event. Nonetheless, considering that our study population was young and therefore with a low incidence of cardiovascular diseases,[3] the proportion of people with history of CVD excluded from the total sample size would have been small; not excluding potentially a small group may

For peer review only - http://bmjopen.bmj.com/site/about/guidelines.xhtml

not have altered the overall results. Third, we only used one blood pressure record (the second SBP out of two measurements). Ideally, and following standard protocols recommended by WHO and other international organizations,[27,28] we should have used the average of multiple records having discarded the very first measurement. This was not possible with the available data because they only measured blood pressure twice. Nonetheless, we performed a sensitivity analysis using the mean SBP of the first and second records and had virtually the same results as in our main analysis in which only the second SBP record was used.

Conclusions

The absolute cardiovascular predicted risk was similar between the laboratory-based and non-laboratory based 2019 WHO cardiovascular risk models. Pending validation from longitudinal studies, the non-laboratory-based model (instead of the laboratory-based which requires additional resources) could be used in Peruvian population. Nonetheless, it should be noted that the agreement between these models was less clear in people with cardiovascular risk factors: obesity, smokers and people with diabetes. While universal health coverage momentum helps to have laboratory tests in (most) primary care facilities to use the laboratory-based model, it seems reasonable to use the non-laboratory-based model for primary prevention of CVD following the risk stratification approach.

For peer review only

For peer review only - http://bmjopen.bmj.com/site/about/guidelines.xhtml

FIGURES

Figure 1. Bland Altman plots showing agreement between laboratory and non-laboratory-based risk scores according to sex.

Figure 2. Bland Altman plots showing agreement between laboratory and non-laboratory-based risk scores according to age groups.

Figure 3. Bland Altman plots showing agreement between laboratory and non-laboratory-based risk scores according to body mass index categories

Figure 4. Bland Altman plots showing agreement between laboratory and non-laboratory-based risk scores according to smoking status.

Figure 5. Bland Altman plots showing agreement between laboratory and non-laboratory-based risk scores according to self-reported diabetes status.

For peer review only

Contributorship: RMC-L conceived the idea with WCG-V. WCG-V conducted the analysis with support from GAQ-V and FFV-R. WCG-V wrote the first draft of the manuscript with support from GAQ-V, FFV-R, AB-O and RMC-L. All authors provided relevant scientific contribution. All authors approved the submitted version.

Funding statement: RMC-L is supported by a Wellcome Trust International Training Fellowship (214185/Z/18/Z). The funder had no role in the study design, analysis, interpretation or decision to publish. The authors are collectively responsible for the accuracy of the data. The arguments and opinions in this work are those of the authors alone, and do not represent the position of the institutions to which they belong.

Competing of interests: None to declare.

Ethics: This study used de-identified nationally-representative survey data that can be requested from the CENAN and is used for independent analyses.[26] Authors had no access to the participants' personal information. Therefore, this work was deemed as of minimal risk and we did not seek approval by an Ethics Committee to conduct the analysis.

Data sharing: Datasets available upon request from the CENAN.

Word count: 4,220

For peer review only

REFERENCES

1. World Health Organization. Cardiovascular diseases (CVDs) [Internet]. [cited 2021 Aug 15]. Available from: https://www.who.int/news-room/fact-sheets/detail/cardiovascular-diseases-(cvds)

2. GBD 2019 Diseases and Injuries Collaborators. Global burden of 369 diseases and injuries in 204 countries and territories, 1990-2019: a systematic analysis for the Global Burden of Disease Study 2019. Lancet. 2020 Oct 17;396(10258):1204–22.

3. GBD Results Tool | GHDx [Internet]. Available from: http://ghdx.healthdata.org/gbd-results-tool

4. Prabhakaran D, Anand S, Watkins D, Gaziano T, Wu Y, Mbanya JC, et al. Cardiovascular, respiratory, and related disorders: key messages from Disease Control Priorities, 3rd edition. Lancet. 2018 Mar 24;391(10126):1224–36.

5. Arnett DK, Blumenthal RS, Albert MA, Buroker AB, Goldberger ZD, Hahn EJ, et al. 2019 ACC/AHA Guideline on the Primary Prevention of Cardiovascular Disease: Executive Summary: A Report of the American College of Cardiology/American Heart Association Task Force on Clinical Practice Guidelines. Circulation. 2019 Sep 10;140(11):e563–95.

6. PAHO/WHO | Pan American Health Organization. HEARTS in the Americas - [Internet]. [cited 2021 Aug 16]. Available from: https://www.paho.org/en/hearts-americas

7. Whelton PK, Carey RM, Aronow WS, Casey DE, Collins KJ, Dennison Himmelfarb C, et al. 2017 ACC/AHA/AAPA/ABC/ACPM/AGS/APhA/ASH/ASPC/NMA/PCNA Guideline for the Prevention, Detection, Evaluation, and Management of High Blood Pressure in Adults: Executive Summary: A Report of the American College of Cardiology/American Heart Association Task Force on Clinical Practice Guidelines. Hypertension. 2018 Jun;71(6):1269–324.

8. Williams B, Mancia G, Spiering W, Agabiti Rosei E, Azizi M, Burnier M, et al. 2018 ESC/ESH Guidelines for the management of arterial hypertension: The Task Force for the management of arterial hypertension of the European Society of Cardiology and the European Society of Hypertension: The Task Force for the management of arterial hypertension of the European Society of Cardiology and the European Society of Hypertension. J Hypertens. 2018 Oct;36(10):1953–2041.

9. NICE National Institute for Health and Care Excellence. Hypertension in adults: diagnosis and management | Guidance [Internet]. NICE; 2019 [cited 2021 Aug 16]. Available from: https://www.nice.org.uk/guidance/ng136

10. Karmali KN, Persell SD, Perel P, Lloyd-Jones DM, Berendsen MA, Huffman MD. Risk scoring for the primary prevention of cardiovascular disease. Cochrane Database Syst Rev. 2017 Mar 14;3:CD006887.

11. Damen JAAG, Hooft L, Schuit E, Debray TPA, Collins GS, Tzoulaki I, et al. Prediction models for cardiovascular disease risk in the general population: systematic review. BMJ. 2016 May 16;353:i2416.

12. WHO CVD Risk Chart Working Group. World Health Organization cardiovascular disease risk charts: revised models to estimate risk in 21 global regions. Lancet Glob Health. 2019 Oct;7(10):e1332–45.

13. Wilson ML, Fleming KA, Kuti MA, Looi LM, Lago N, Ru K. Access to pathology and laboratory medicine services: a crucial gap. Lancet. 2018 May 12;391(10133):1927–38.

14. Marcus ME, Manne-Goehler J, Theilmann M, Farzadfar F, Moghaddam SS, Keykhaei M, et al. Use of statins for the prevention of cardiovascular disease in 41 low-income and

For peer review only - http://bmjopen.bmj.com/site/about/guidelines.xhtml

middle-income countries: a cross-sectional study of nationally representative, individual-level data. Lancet Glob Health. 2022 Mar;10(3):e369–79.

15. Centro Nacional de Alimentación y Nutrición. ESTADO NUTRICIONAL EN ADULTOS DE 18 A 59 AÑOS, PERÚ: 2017 - 2018 [Internet]. 2021. Available from: https://web.ins.gob.pe/sites/default/files/Archivos/cenan/van/sala_nutricional/sala_3/2021/Informe%20Tecnico-%20Estado%20nutricional%20en%20adultos%20de%2018%20a%2059%20a%C3%B1os%2CVIANEV%202017-2018.pdf

16. Package of essential noncommunicable (PEN) disease interventions for primary health care in low-resource setting. WHO PEN Protocol 1 Prevention of HEart Attacks, Stroke and Kidney Disease through Integrated Management of Diabetes and Hypertension [Internet]. Available from: https://www.who.int/ncds/management/Protocol1_HeartAttack_strokes_kidneyDisease.pdf?ua=1

17. Peiris D, Ghosh A, Manne-Goehler J, Jaacks LM, Theilmann M, Marcus ME, et al. Cardiovascular disease risk profile and management practices in 45 low-income and middle-income countries: A cross-sectional study of nationally representative individual-level survey data. PLoS Med. 2021 Mar;18(3):e1003485.

18. University of Cambridge. Cardiovascular Epidemiology Unit. Programs [Internet]. Cardiovascular Epidemiology Unit. [cited 2021 Aug 15]. Available from: https://www.phpc.cam.ac.uk/ceu/population-resources-and-tools/programs/

19. Law MR, Morris JK, Wald NJ. Use of blood pressure lowering drugs in the prevention of cardiovascular disease: meta-analysis of 147 randomised trials in the context of expectations from prospective epidemiological studies. BMJ. 2009 May 19;338:b1665.

20. Bland JM, Altman DG. Statistical methods for assessing agreement between two methods of clinical measurement. Lancet. 1986 Feb 8;1(8476):307–10.

21. Jones CA, Ross L, Surani N, Dharamshi N, Karmali K. Framingham ten-year general cardiovascular disease risk: agreement between BMI-based and cholesterol-based estimates in a South Asian convenience sample. PLoS One. 2015;10(3):e0119183.

22. Viera AJ, Garrett JM. Understanding interobserver agreement: the kappa statistic. Fam Med. 2005 May;37(5):360–3.

23. Rezaei F, Seif M, Gandomkar A, Fattahi MR, Hasanzadeh J. Agreement between laboratory-based and non-laboratory-based Framingham risk score in Southern Iran. Sci Rep. 2021 May 24;11(1):10767.

24. Gobierno del Perú. Conocer establecimientos de salud del Primer Nivel de Atención [Internet]. 2022 [cited 2022 May 17]. Available from: https://www.gob.pe/16727

25. D'Agostino RB, Vasan RS, Pencina MJ, Wolf PA, Cobain M, Massaro JM, et al. General cardiovascular risk profile for use in primary care: the Framingham Heart Study. Circulation. 2008 Feb 12;117(6):743–53.

26. Guzman-Vilca WC, Yovera-Juarez EA, Tarazona-Meza C, García-Larsen V, Carrillo-Larco RM. Sugar-Sweetened Beverage Consumption in Adults: Evidence from a National Health Survey in Peru. Nutrients. 2022 Jan 28;14(3):582.

27. World Health Organization. STEPwise Approach to NCD Risk Factor Surveillance (STEPS) [Internet]. [cited 2022 Jan 10]. Available from: https://www.who.int/teams/noncommunicable-diseases/surveillance/systems-tools/steps

For peer review only

28. Poulter NR, Borghi C, Damasceno A, Jafar TH, Khan N, Kokubo Y, et al. May Measurement Month 2019: results of blood pressure screening from 47 countries. Eur Heart J Suppl. 2021 May;23(Suppl B):B1–5.

For peer review only

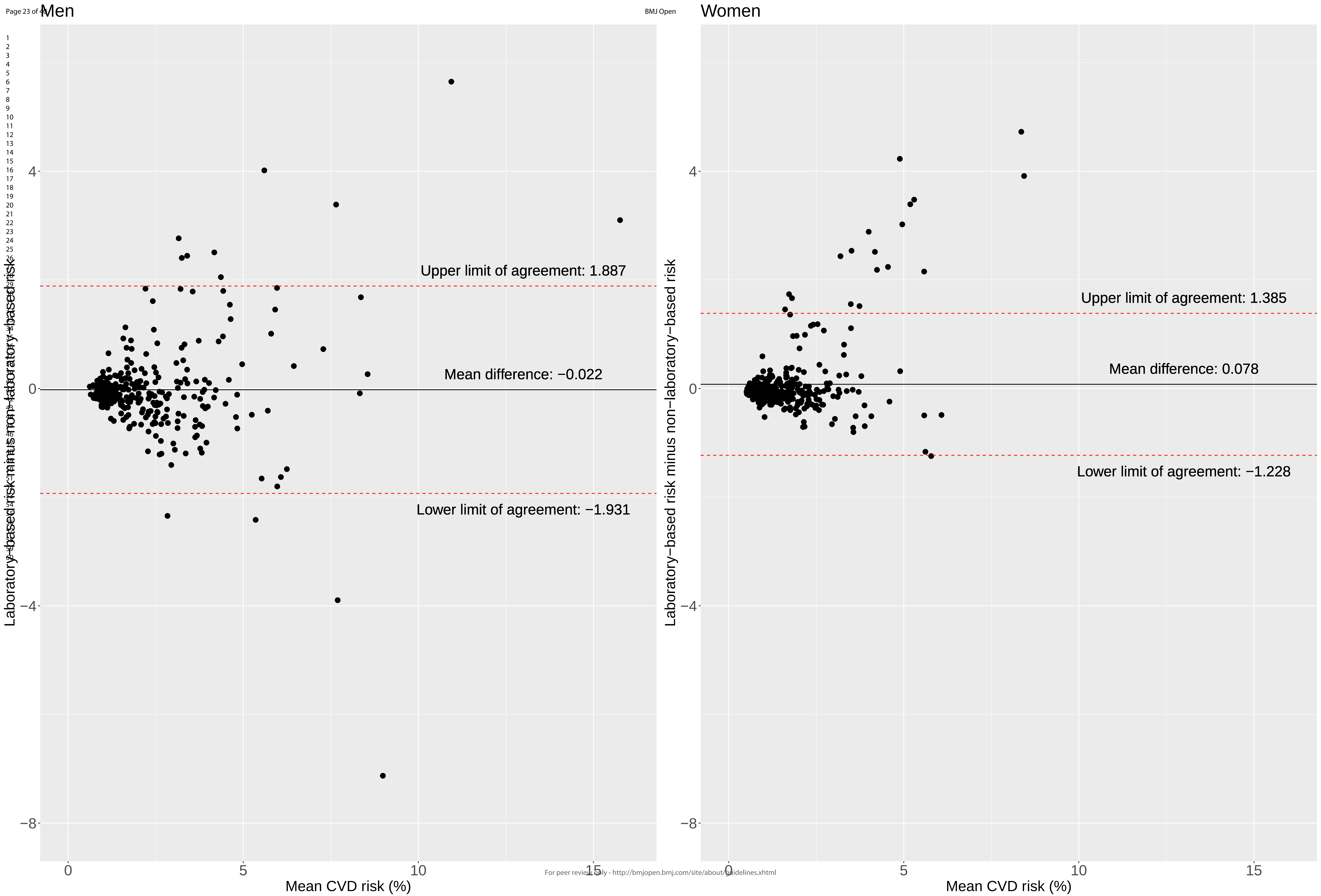
Men
Upper limit of agreement: 1.887
Mean difference: −0.022
Lower limit of agreement: −1.931
Laboratory−based risk minus non−laboratory−based risk
Mean CVD risk (%)
Women
Upper limit of agreement: 1.385
Mean difference: 0.078
Lower limit of agreement: −1.228
Laboratory−based risk minus non−laboratory−based risk
Mean CVD risk (%)

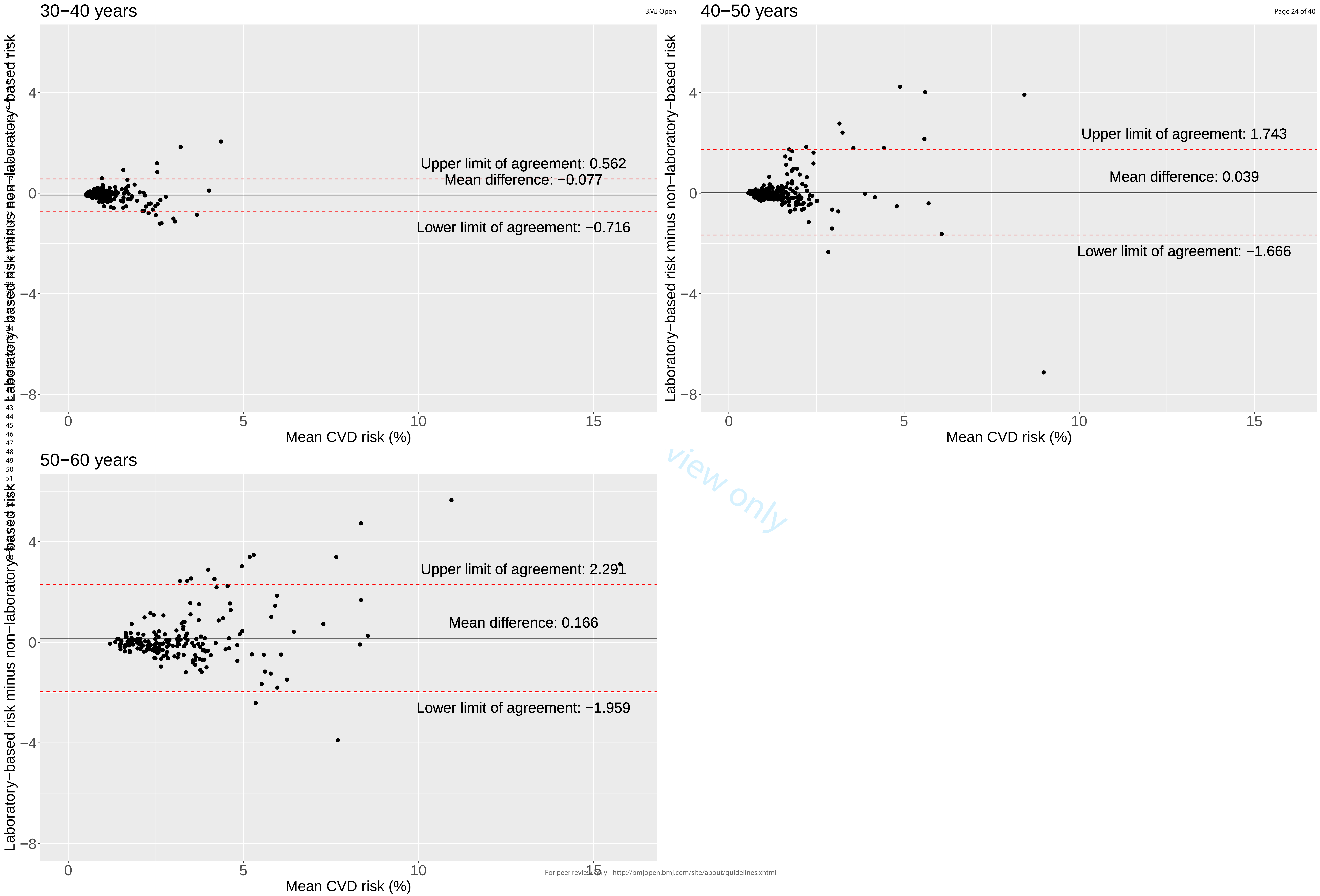
30−40 years
Upper limit of agreement: 0.562
Mean difference: −0.077
Lower limit of agreement: −0.716
Laboratory−based risk minus non−laboratory−based risk
Mean CVD risk (%)
40−50 years
Upper limit of agreement: 1.743
Mean difference: 0.039
Lower limit of agreement: −1.666
Laboratory−based risk minus non−laboratory−based risk
Mean CVD risk (%)
50−60 years
Upper limit of agreement: 2.291
Mean difference: 0.166
Lower limit of agreement: −1.959
Laboratory−based risk minus non−laboratory−based risk
Mean CVD risk (%)

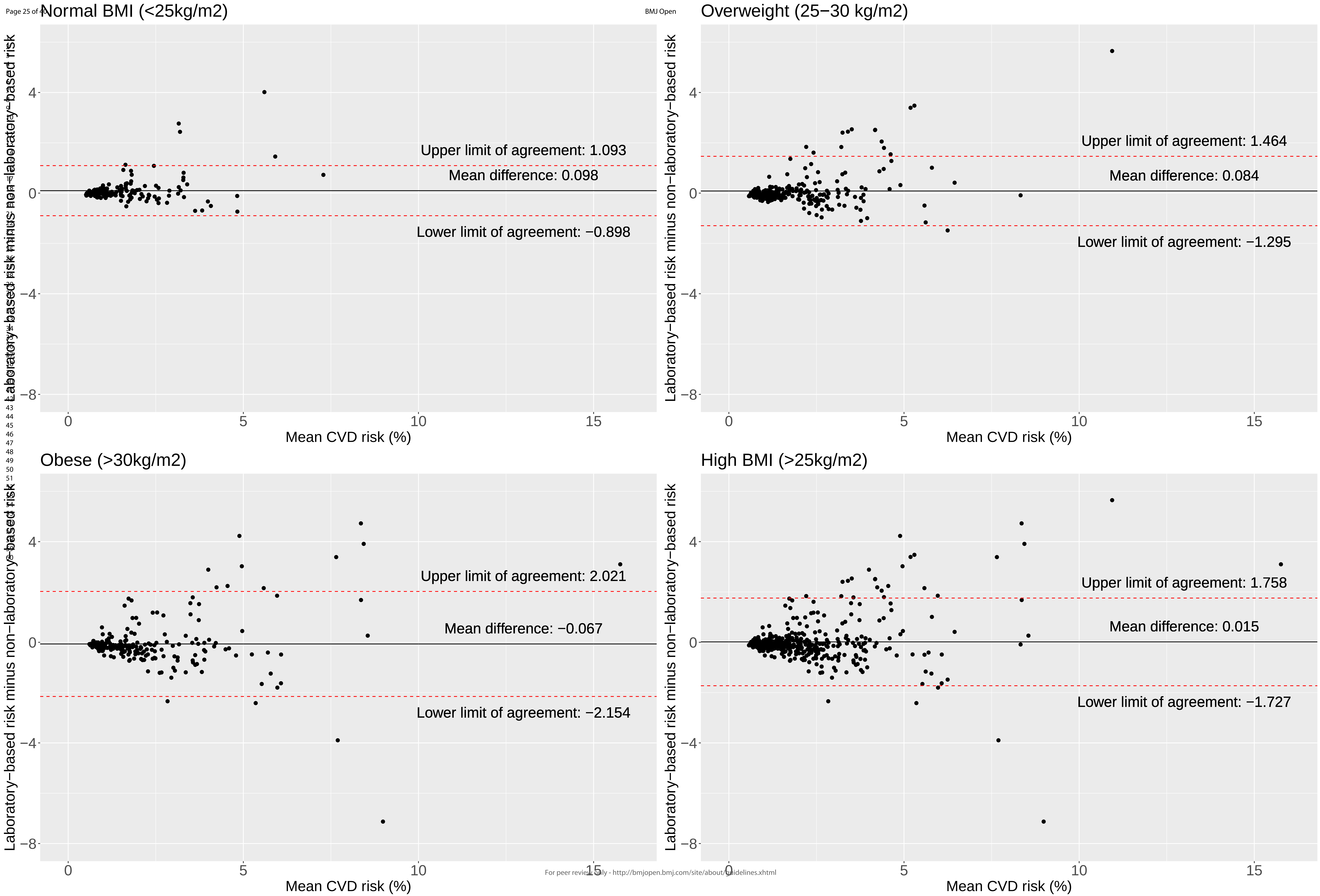
Normal BMI (<25kg/m2)
Upper limit of agreement: 1.093
Mean difference: 0.098
Lower limit of agreement: −0.898
Overweight (25−30 kg/m2)
Upper limit of agreement: 1.464
Mean difference: 0.084
Lower limit of agreement: −1.295
Obese (>30kg/m2)
Upper limit of agreement: 2.021
Mean difference: −0.067
Lower limit of agreement: −2.154
High BMI (>25kg/m2)
Upper limit of agreement: 1.758
Mean difference: 0.015
Lower limit of agreement: −1.727
Laboratory−based risk minus non−laboratory−based risk
Mean CVD risk (%)
4
0
−4
−8
0
5
10
15

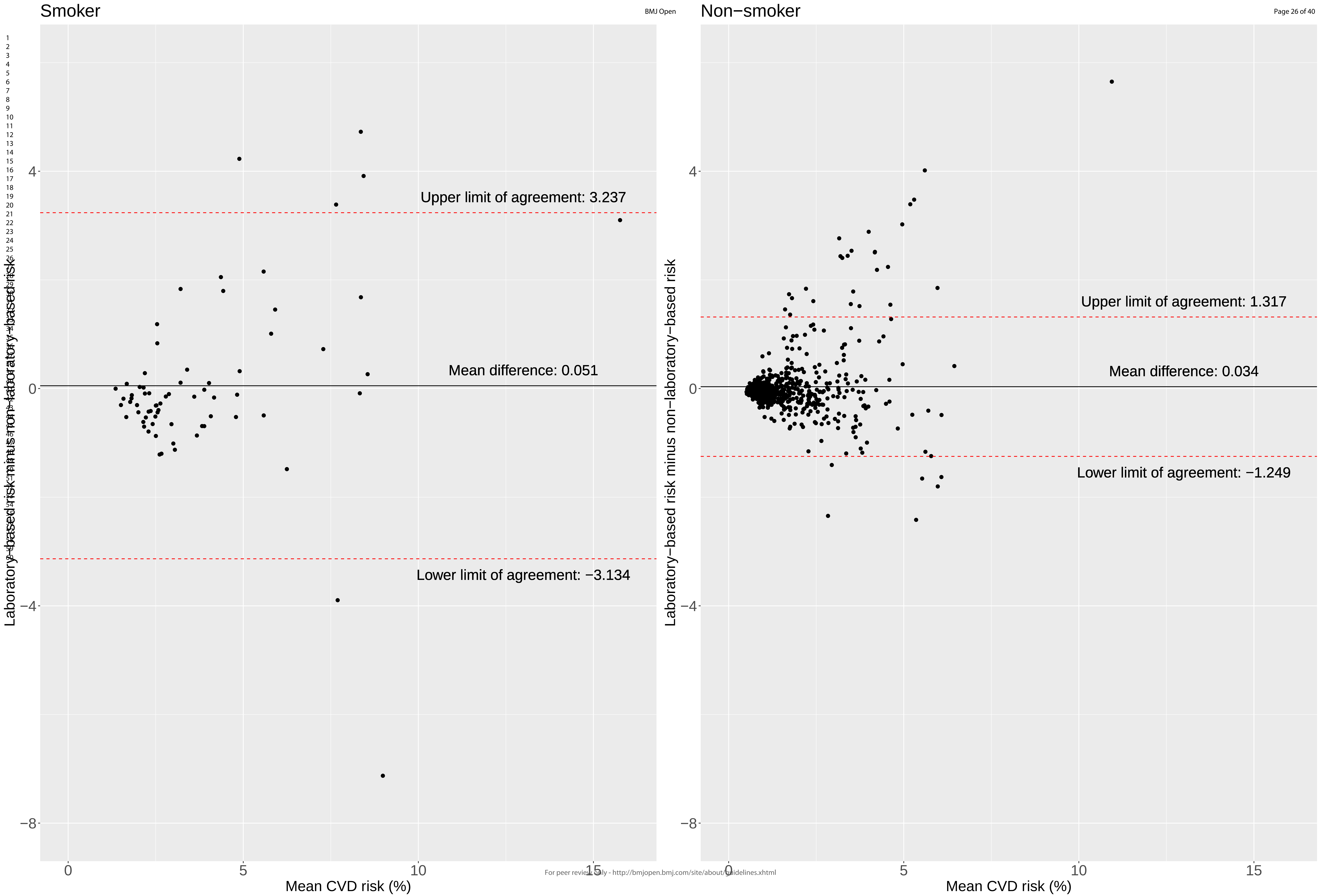
Smoker
Upper limit of agreement: 3.237
Mean difference: 0.051
Lower limit of agreement: −3.134
Laboratory−based risk minus non−laboratory−based risk
Mean CVD risk (%)
Non−smoker
Upper limit of agreement: 1.317
Mean difference: 0.034
Lower limit of agreement: −1.249
Laboratory−based risk minus non−laboratory−based risk
Mean CVD risk (%)

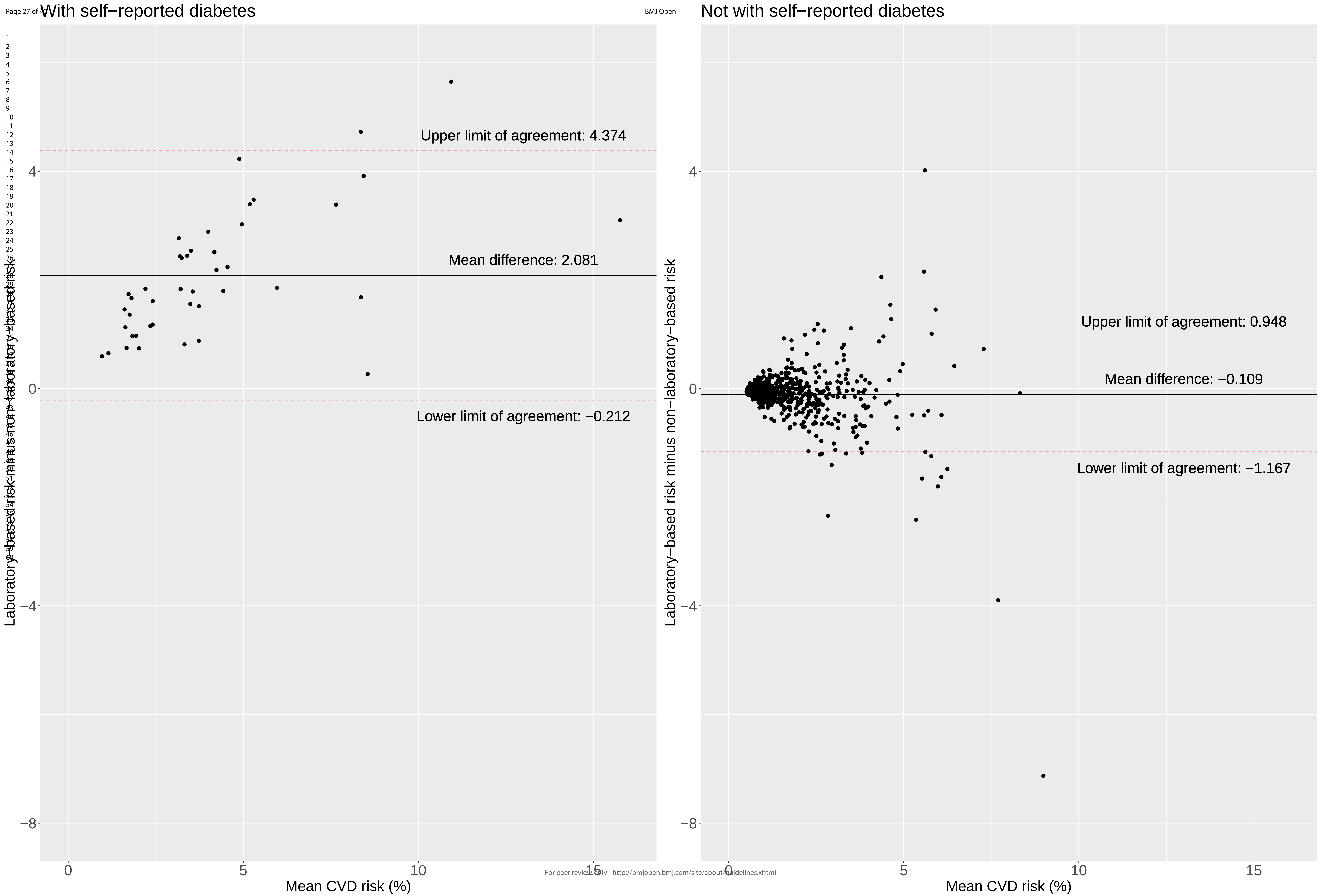
With self−reported diabetes
Not with self−reported diabetes
Upper limit of agreement: 4.374
Mean difference: 2.081
Lower limit of agreement: −0.212
Upper limit of agreement: 0.948
Mean difference: −0.109
Lower limit of agreement: −1.167
Laboratory−based risk minus non−laboratory−based risk
Mean CVD risk (%)
4
0
−4
−8
0
5
10
15

Agreement between the laboratory- and non-laboratory-based WHO cardiovascular risk charts: a cross-sectional analysis of a national health survey in Peru

Correspondence author:

Rodrigo M Carrillo-Larco, MD

Department of Epidemiology and Biostatistics

School of Public Health

Imperial College London

rcarrill@ic.ac.uk

For peer review only

For peer review only - http://bmjopen.bmj.com/site/about/guidelines.xhtml

Table of Contents

Table of Contents 2
Supplementary Figure 1. Formula to compute the survey sample size........ 3
Supplementary Figure 2. Flowchart of data cleaning and inclusion criteria........ 3
Supplementary Table 1. Summary statistics of the first and second systolic blood pressure (SBP) records in the overall sample........ 4
Supplementary Table 2. Sensitivity Analysis: Lin's concordance coefficient correlation showing agreement between laboratory and non-laboratory-based risk models according to the predictors in the 2019 WHO CVD risk models and urban/rural location........ 4
Supplementary Table 3. Kappa statistics showing agreement between laboratory and non-laboratory-based risk scores in the overall sample........ 5
Supplementary Table 4. Kappa statistics showing agreement between laboratory and non-laboratory-based risk scores by sex........ 5
Supplementary Table 5. Kappa statistics showing agreement between laboratory and non-laboratory-based risk scores by age groups........ 5
Supplementary Table 6. Kappa statistics showing agreement between laboratory and non-laboratory-based risk scores by body mass index categories........ 6
Supplementary Table 7. Kappa statistics showing agreement between laboratory and non-laboratory-based risk scores by smoking status........ 6
Supplementary Table 8. Kappa statistics showing agreement between laboratory and non-laboratory-based risk scores by diabetes status........ 6
Supplementary Table 9. Kappa statistics showing agreement between laboratory and non-laboratory-based risk scores by urban/rural location........ 7
Supplementary Figure 3. Sensitivity Analysis: Bland Altman plots showing agreement between laboratory and non-laboratory-based risk scores according to sex. 7
Supplementary Figure 4. Sensitivity Analysis: Bland Altman plots showing agreement between laboratory and non-laboratory-based risk scores according to age groups........ 8
Supplementary Figure 5. Sensitivity Analysis: Bland Altman plots showing agreement between laboratory and non-laboratory-based risk scores according to body mass index categories. 9
Supplementary Figure 6. Sensitivity Analysis: Bland Altman plots showing agreement between laboratory and non-laboratory-based risk scores according to smoking status. 10
Supplementary Figure 7. Sensitivity Analysis: Bland Altman plots showing agreement between laboratory and non-laboratory-based risk scores according to self-reported diabetes status........ 11

For peer review only

For peer review only - http://bmjopen.bmj.com/site/about/guidelines.xhtml

Supplementary Figure 1. Formula to compute the survey sample size

$$n_h = \frac{N_h Z^2 P_h Q_h}{(N_h - 1)d^2 + Z^2 P_h Q_h} * TNR$$

Where:
N_h: number of people within an age group in the “h” conglomerate
N_h: number of people in the sample within an age group in the “h” conglomerate
d: error margin assumed in the estimation of P_h
Z: 95% confidence level
TNR (*Tasa de No Respuesta* in Spanish): Expected refusal rate
P_h: prevalence of overweight in adults in the “h” conglomerate

Supplementary Figure 2. Flowchart of data cleaning and inclusion criteria

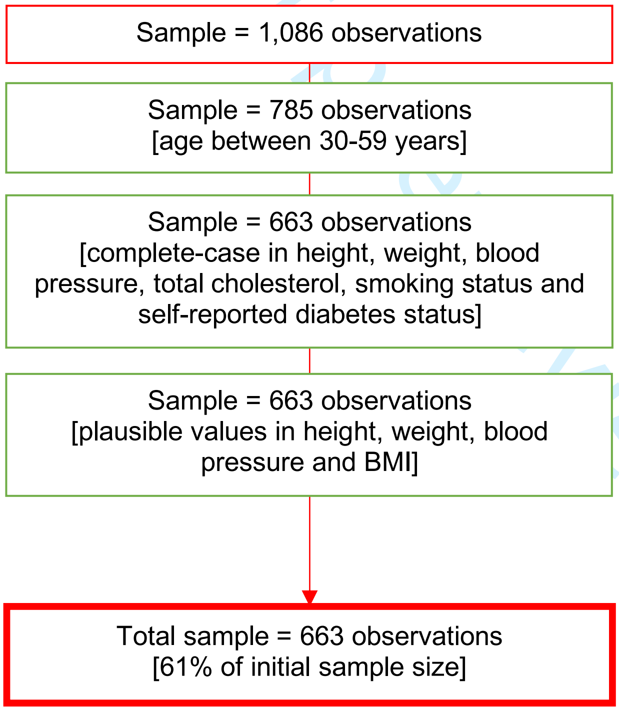

For peer review only

Supplementary Table 1. Summary statistics of the first and second systolic blood pressure (SBP) records in the overall sample

	First SBP record	Second SBP record
**Minimum value**	78	75
**1st quartile**	101	99
**Median**	109	108
**Mean**	110.33	109.54
**3rd quartile**	118	118
**Maximum value**	203	198

Supplementary Table 2. Sensitivity Analysis: Lin's concordance coefficient correlation showing agreement between laboratory and non-laboratory-based risk models according to the predictors in the 2019 WHO CVD risk models and urban/rural location

Variables	Categories	Lin's concordance coefficient correlation (95% CI)
**Sex**	**Men**	0.87 (0.84 - 0.89)
**Sex**	**Women**	0.85 (0.83 - 0.87)
**Age (years)**	**30-39**	0.87 (0.84 - 0.9)
**Age (years)**	**40-49**	0.74 (0.68 - 0.79)
**Age (years)**	**50-59**	0.83 (0.78 - 0.87)
**Body mass index category**	**Normal**	0.9 (0.87 - 0.92)
**Body mass index category**	**Overweight**	0.87 (0.85 - 0.9)
**Body mass index category**	**Obese**	0.86 (0.82 - 0.89)
**Smoking status**	**Smoker**	0.82 (0.73 - 0.88)
**Smoking status**	**Non-smoker**	0.86 (0.84 - 0.88)
**Diabetes status**	**With self−reported diabetes**	0.74 (0.63 - 0.82)
**Diabetes status**	**Not with self−reported diabetes**	0.91 (0.9 - 0.92)
**Urban or rural**	**Urban**	0.88 (0.86 - 0.9)
**Urban or rural**	**Rural**	0.86 (0.82 - 0.88)

For peer review only - http://bmjopen.bmj.com/site/about/guidelines.xhtml

Supplementary Table 3. Kappa statistics showing agreement between laboratory and non-laboratory-based risk scores in the overall sample

	Non-laboratory-based-risk category			
**Laboratory-based-risk category**	**0-5**	**5-9**	**10-19**	**kappa**
**0-5**	618	4	0	0.62
**5-9**	17	19	1	
**10-19**	0	3	1	

Supplementary Table 4. Kappa statistics showing agreement between laboratory and non-laboratory-based risk scores by sex

		Non-laboratory-based-risk category			
**Laboratory-based-risk category**	**Sex**	**0-5**	**5-9**	**10-19**	**kappa**
**0-5**	Men	251	4	0	0.7
**5-9**	Men	7	15	1	
**10-19**	Men	0	1	1	
**0-5**	Women	367	0	0	0.44
**5-9**	Women	10	4	0	
**10-19**	Women	0	2	0	

Supplementary Table 5. Kappa statistics showing agreement between laboratory and non-laboratory-based risk scores by age groups

		Non-laboratory-based-risk category			
**Laboratory-based-risk category**	**Age group**	**0-5**	**5-9**	**10-19**	**kappa**
**0-5**	30-39	236	0	0	0
**5-9**	30-39	1	0	0	
**10-19**	30-39	0	0	0	
**0-5**	40-49	216	1	0	0.45
**5-9**	40-49	4	2	1	
**10-19**	40-49	0	1	0	
**0-5**	50-59	166	3	0	0.65
**5-9**	50-59	12	17	0	
**10-19**	50-59	0	2	1	

For peer review only - http://bmjopen.bmj.com/site/about/guidelines.xhtml

Supplementary Table 6. Kappa statistics showing agreement between laboratory and non-laboratory-based risk scores by body mass index categories

		Non-laboratory-based-risk category			
**Laboratory-based-risk category**	**Body mass index category**	**0-5**	**5-9**	**10-19**	**kappa**
**0-5**	Normal	161	1	0	0.66
**5-9**	Normal	1	2	0	
**10-19**	Normal	0	0	0	
**0-5**	Obese	202	3	0	0.65
**5-9**	Obese	7	11	1	
**10-19**	Obese	0	2	1	
**0-5**	Overweight	255	0	0	0.55
**5-9**	Overweight	9	6	0	
**10-19**	Overweight	0	1	0	

Supplementary Table 7. Kappa statistics showing agreement between laboratory and non-laboratory-based risk scores by smoking status

		Non-laboratory-based-risk category			
**Laboratory-based-risk category**	**Smoking status**	**0-5**	**5-9**	**10-19**	**kappa**
**0-5**	Non-smoker	568	3	0	0.53
**5-9**	Non-smoker	12	9	0	
**10-19**	Non-smoker	0	1	0	
**0-5**	Smoker	50	1	0	0.67
**5-9**	Smoker	5	10	1	
**10-19**	Smoker	0	2	1	

Supplementary Table 8. Kappa statistics showing agreement between laboratory and non-laboratory-based risk scores by diabetes status

For peer review only - http://bmjopen.bmj.com/site/about/guidelines.xhtml

		Non-laboratory-based-risk category			
**Laboratory-based-risk category**	**Diabetes status**	**0-5**	**5-9**	**10-19**	**kappa**
**0-5**	Not with self−reported diabetes	592	4	0	0.71
**5-9**	Not with self−reported diabetes	7	15	1	
**10-19**	Not with self−reported diabetes	0	0	0	
**0-5**	With self−reported diabetes	26	0	0	0.36
**5-9**	With self−reported diabetes	10	4	0	
**10-19**	With self−reported diabetes	0	3	1	

Supplementary Table 9. Kappa statistics showing agreement between laboratory and non-laboratory-based risk scores by urban/rural location

		Non-laboratory-based-risk category			
**Laboratory-based-risk category**	**Urban/Rural**	**0-5**	**5-9**	**10-19**	**kappa**
**0-5**	Rural	224	1	0	0.53
**5-9**	Rural	4	3	0	
**10-19**	Rural	0	1	0	
**0-5**	Urban	394	3	0	0.64
**5-9**	Urban	13	16	1	
**10-19**	Urban	0	2	1	

Supplementary Figure 3. Sensitivity Analysis: Bland Altman plots showing agreement between laboratory and non-laboratory-based risk scores according to sex.

For peer review only - http://bmjopen.bmj.com/site/about/guidelines.xhtml

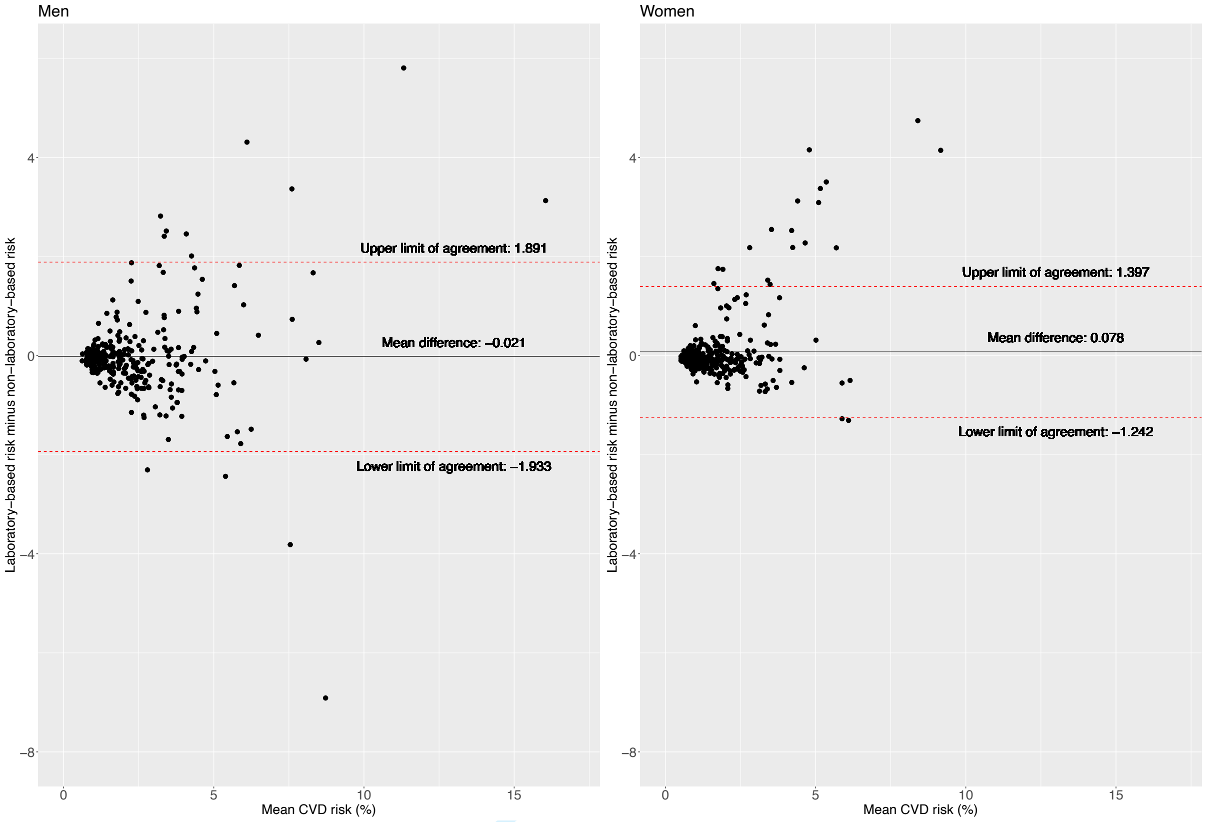

Supplementary Figure 4. Sensitivity Analysis: Bland Altman plots showing agreement between laboratory and non-laboratory-based risk scores according to age groups.

For peer review only - http://bmjopen.bmj.com/site/about/guidelines.xhtml

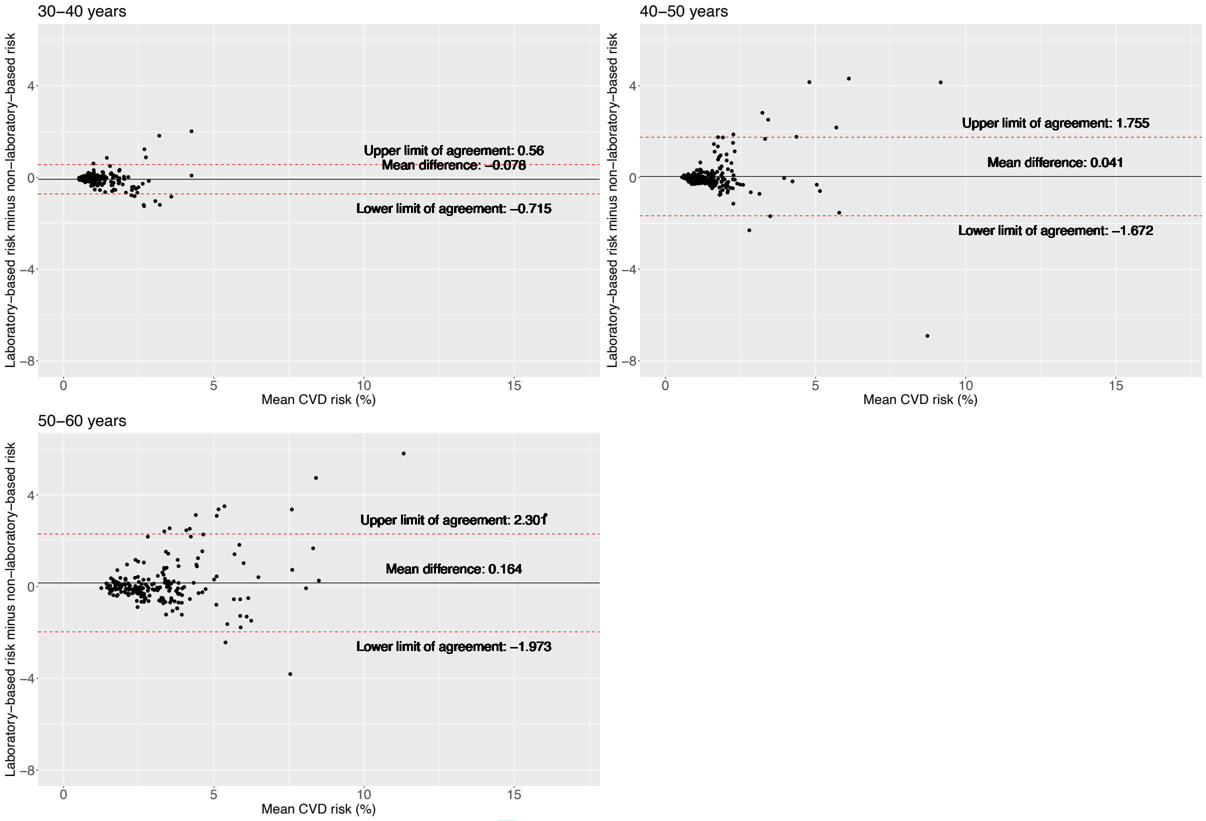

Supplementary Figure 5. Sensitivity Analysis: Bland Altman plots showing agreement between laboratory and non-laboratory-based risk scores according to body mass index categories.

For peer review only - http://bmjopen.bmj.com/site/about/guidelines.xhtml

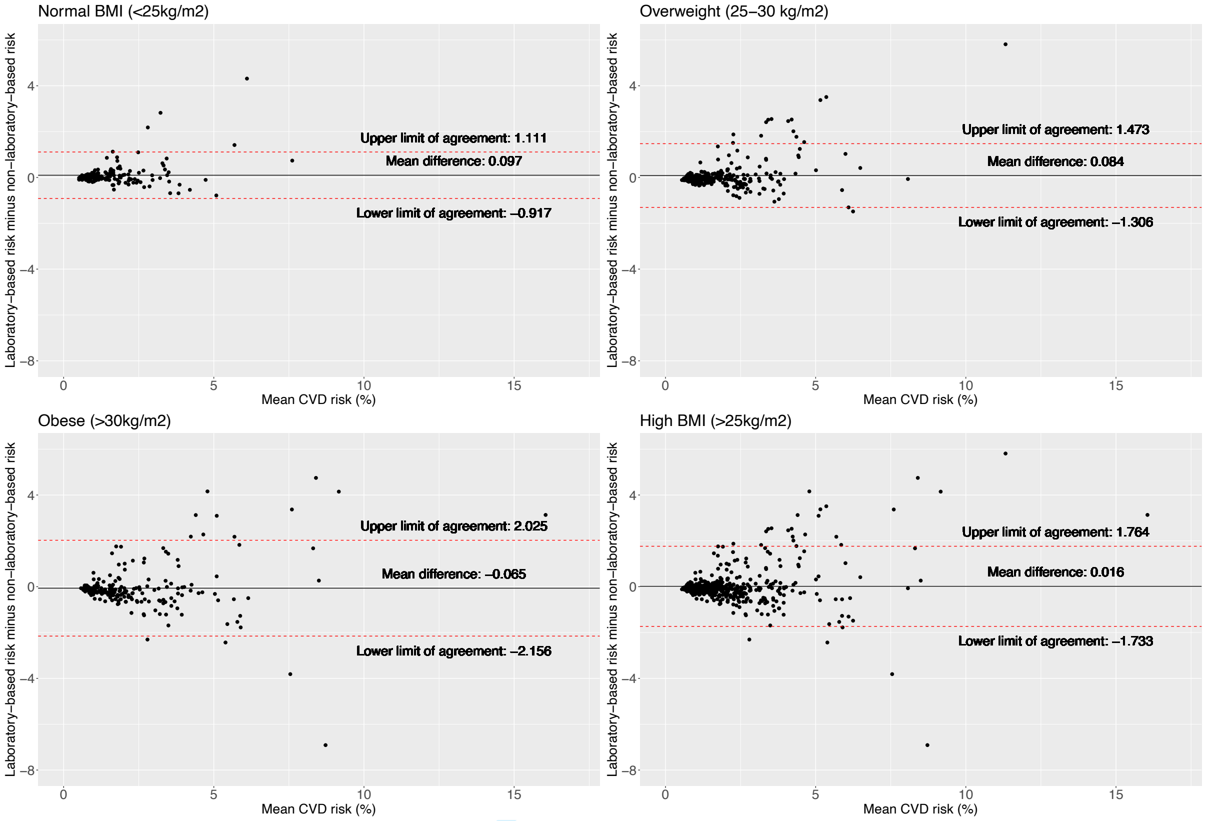

Supplementary Figure 6. Sensitivity Analysis: Bland Altman plots showing agreement between laboratory and non-laboratory-based risk scores according to smoking status.

For peer review only - http://bmjopen.bmj.com/site/about/guidelines.xhtml

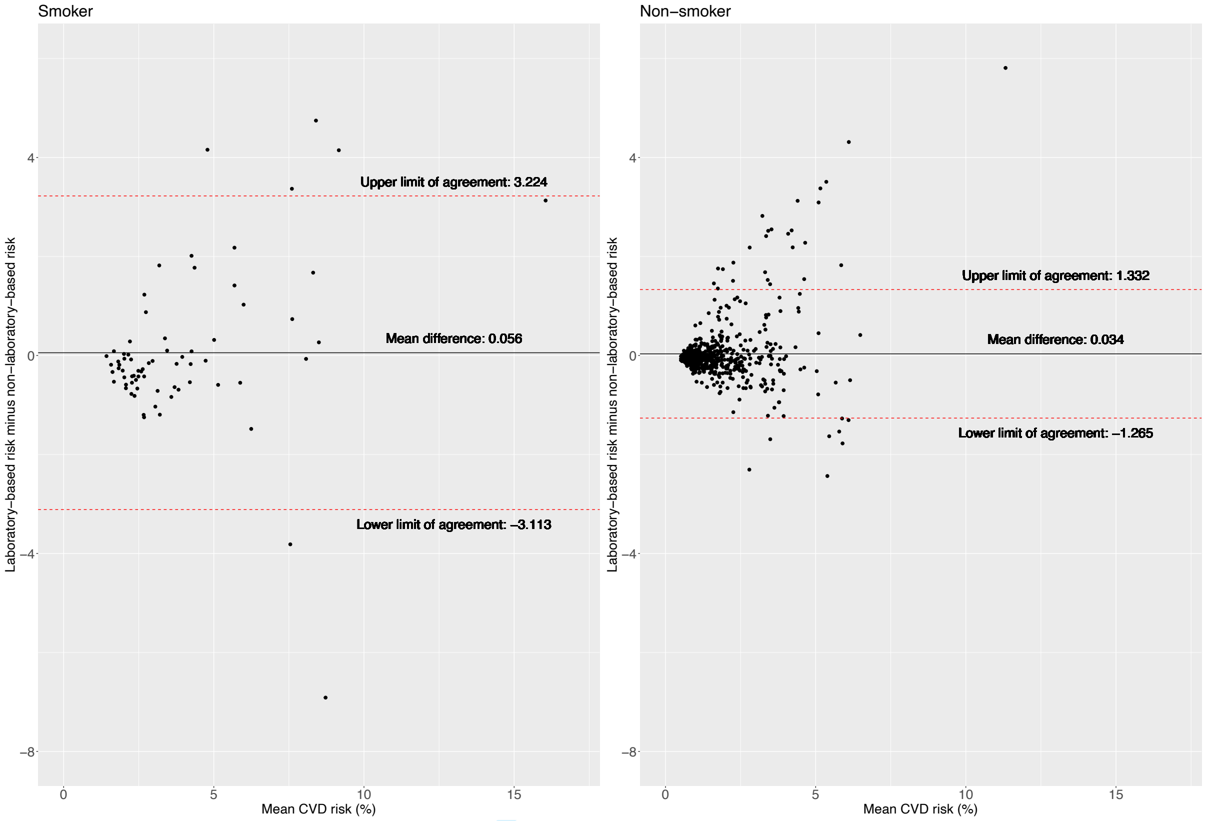

Supplementary Figure 7. Sensitivity Analysis: Bland Altman plots showing agreement between laboratory and non-laboratory-based risk scores according to self-reported diabetes status.

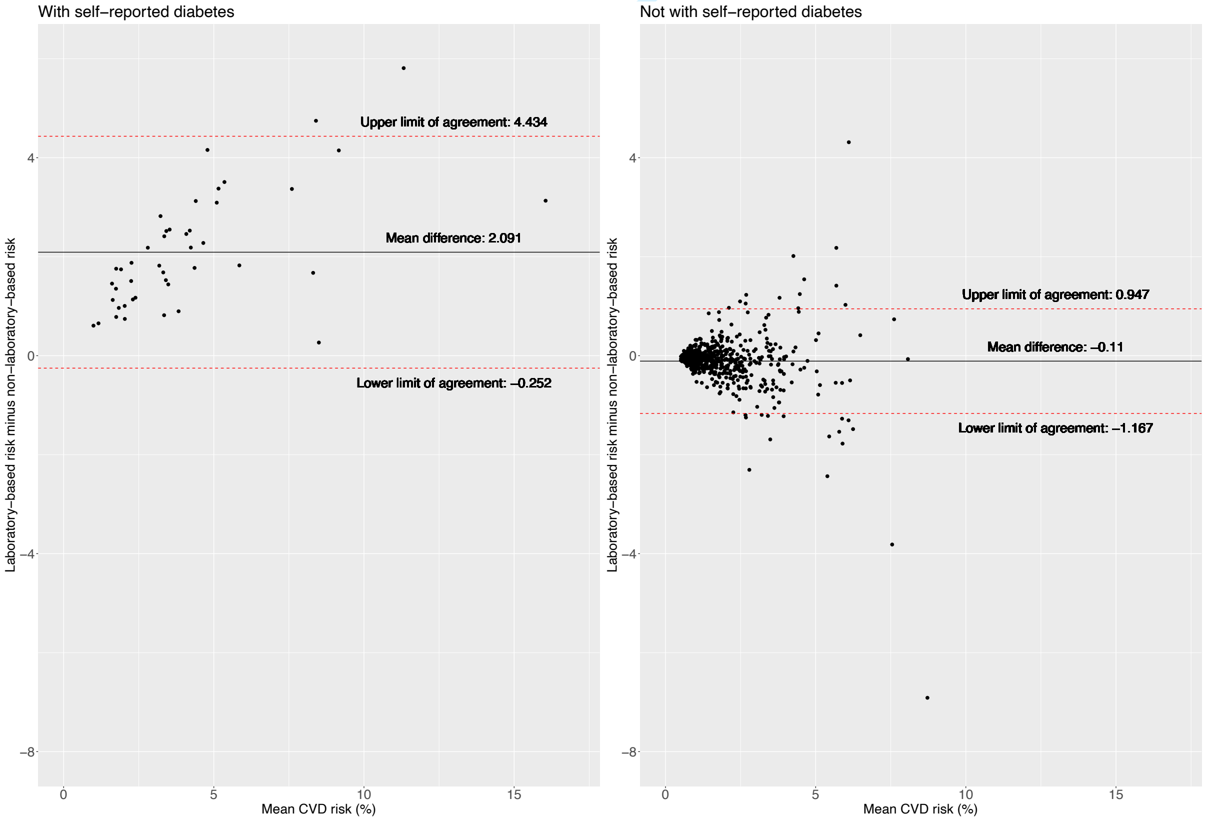

For peer review only

For peer review only - http://bmjopen.bmj.com/site/about/guidelines.xhtml

STROBE Statement—Checklist of items that should be included in reports of ***cross-sectional studies***

	Item No	Recommendation	Page No
**Title and abstract**	1	(*a*) Indicate the study's design with a commonly used term in the title or the abstract	3
		(*b*) Provide in the abstract an informative and balanced summary of what was done and what was found	3
**Introduction**			
Background/rationale	2	Explain the scientific background and rationale for the investigation being reported	4
Objectives	3	State specific objectives, including any prespecified hypotheses	5
**Methods**			
Study design	4	Present key elements of study design early in the paper	5
Setting	5	Describe the setting, locations, and relevant dates, including periods of recruitment, exposure, follow-up, and data collection	5-6
Participants	6	(*a*) Give the eligibility criteria, and the sources and methods of selection of participants	5-6
Variables	7	Clearly define all outcomes, exposures, predictors, potential confounders, and effect modifiers. Give diagnostic criteria, if applicable	6-7
Data sources/ measurement	8*	For each variable of interest, give sources of data and details of methods of assessment (measurement). Describe comparability of assessment methods if there is more than one group	*6-7*
Bias	9	Describe any efforts to address potential sources of bias	6
Study size	10	Explain how the study size was arrived at	5-6
Quantitative variables	11	Explain how quantitative variables were handled in the analyses. If applicable, describe which groupings were chosen and why	6-7
Statistical methods	12	(*a*) Describe all statistical methods, including those used to control for confounding	7-8
		(*b*) Describe any methods used to examine subgroups and interactions	7-8
		(*c*) Explain how missing data were addressed	5-6
		(*d*) If applicable, describe analytical methods taking account of sampling strategy	8
		(*e*) Describe any sensitivity analyses	6
**Results**			
Participants	13*	(a) Report numbers of individuals at each stage of study—eg numbers potentially eligible, examined for eligibility, confirmed eligible, included in the study, completing follow-up, and analysed	8
		(b) Give reasons for non-participation at each stage	Supp Fig 1
		(c) Consider use of a flow diagram	5
Descriptive data	14*	(a) Give characteristics of study participants (eg demographic, clinical, social) and information on exposures and potential confounders	8
		(b) Indicate number of participants with missing data for each variable of interest	Supp Fig 1
Outcome data	15*	Report numbers of outcome events or summary measures	

For peer review only

For peer review only - http://bmjopen.bmj.com/site/about/guidelines.xhtml

Main results	16	(*a*) Give unadjusted estimates and, if applicable, confounder-adjusted estimates and their precision (eg, 95% confidence interval). Make clear which confounders were adjusted for and why they were included	8-10
		(*b*) Report category boundaries when continuous variables were categorized	8-10
		(*c*) If relevant, consider translating estimates of relative risk into absolute risk for a meaningful time period	-
Other analyses	17	Report other analyses done—eg analyses of subgroups and interactions, and sensitivity analyses	10
**Discussion**			
Key results	18	Summarise key results with reference to study objectives	11
Limitations	19	Discuss limitations of the study, taking into account sources of potential bias or imprecision. Discuss both direction and magnitude of any potential bias	13
Interpretation	20	Give a cautious overall interpretation of results considering objectives, limitations, multiplicity of analyses, results from similar studies, and other relevant evidence	11-12
Generalisability	21	Discuss the generalisability (external validity) of the study results	11-12
**Other information**			
Funding	22	Give the source of funding and the role of the funders for the present study and, if applicable, for the original study on which the present article is based	2

*Give information separately for exposed and unexposed groups.

Note: An Explanation and Elaboration article discusses each checklist item and gives methodological background and published examples of transparent reporting. The STROBE checklist is best used in conjunction with this article (freely available on the Web sites of PLoS Medicine at http://www.plosmedicine.org/, Annals of Internal Medicine at http://www.annals.org/, and Epidemiology at http://www.epidem.com/). Information on the STROBE Initiative is available at www.strobe-statement.org.

For peer review only
